# *Lactobacillus acidophilus* Membrane Vesicles as a Vehicle of Bacteriocin Delivery

**DOI:** 10.3389/fmicb.2020.00710

**Published:** 2020-04-30

**Authors:** Scott N. Dean, Mary Ashley Rimmer, Kendrick B. Turner, Daniel A. Phillips, Julie C. Caruana, William Judson Hervey, Dagmar H. Leary, Scott A. Walper

**Affiliations:** ^1^National Research Council Associate, Washington, DC, United States; ^2^US Naval Research Laboratory, Center for Bio/Molecular Science & Engineering (Code 6900), Washington, DC, United States; ^3^American Society for Engineering Education Associate, Washington, DC, United States

**Keywords:** membrane vesicles, bacteriocins, lactic acid bacteria, *Lactobacillus acidophilus*, antimicrobial

## Abstract

Recent reports have shown that Gram-positive bacteria actively secrete spherical nanometer-sized proteoliposome membrane vesicles (MVs) into their surroundings. Though MVs are implicated in a broad range of biological functions, few studies have been conducted to examine their potential as delivery vehicles of antimicrobials. Here, we investigate the natural ability of *Lactobacillus acidophilus* MVs to carry and deliver bacteriocin peptides to the opportunistic pathogen, *Lactobacillus delbrueckii*. We demonstrate that upon treatment with lactacin B-inducing peptide, the proteome of the secreted MVs is enriched in putative bacteriocins encoded by the *lab* operon. Further, we show that purified MVs inhibit growth and compromise membrane integrity in *L. delbrueckii*, which is confirmed by confocal microscopy imaging and spectrophotometry. These results show that *L. acidophilus* MVs serve as conduits for antimicrobials to competing cells in the environment, suggesting a potential role for MVs in complex communities such as the gut microbiome. With the potential for controlling their payload through microbial engineering, MVs produced by *L. acidophilus* may be an interesting platform for effecting change in complex microbial communities or aiding in the development of new biomedical therapeutics.

## Introduction

Microbial communities such as the gut microbiota are comprised of numerous microorganisms that persist in a steady state of competition and cooperation. Chemical signals, metabolites, and other biomolecules constantly flood the community, effecting changes to the composition of the community as individual species struggle to remain viable. The human gastrointestinal (GI) tract contains at least 1000 species of bacteria, where they grow up to 10^11^ organisms per gram of intestinal material (Rajilic-Stojanovic and de Vos, 2014). Beyond contributing to digestion of foodstuffs, these GI bacteria perform many other functions for the host, including essential vitamin production, education of the immune system, communication with cells of the intestines, and altering behavior of the host (Backhed et al., 2005; Lathrop et al., 2011; Cryan and Dinan, 2012; Rajilic-Stojanovic, 2013). In the human GI tract, home to many diverse bacteria, both commensal and potentially pathogenic, lactic acid bacteria typically constitute a small portionl of the bacterial population. Detailed in a review by Walter, lactobacilli abundance within fecal samples ranges from 0.01 to 6.6% with the upper value including members of the *Enterococcus* genus (Walter, 2008; Yoneyama et al., 2009). To remain viable members of this diverse population of competing microorganisms, bacteria have evolved an arsenal of mechanisms to kill other species, including the production of lactic acid, reactive oxygen species, and bacteriocin peptides (Rajilic-Stojanovic and de Vos, 2014).

Bacteriocins are a subgroup of antimicrobial peptides that have bactericidal activity toward some closely related species, while the producer species exhibits specific immunity due to an inherent property of the bacterial physiology (Perez et al., 2014). There are an estimated 74 distinct bacteriocin-encoding clusters found in the GI tract, based on the human microbiome project reference genome database (Walsh et al., 2015). Many of the identified clusters were found in lactobacilli and have been reported to enable the killing of not only some pathogenic organisms but also other commensal, including members of the *Lactobacillus* genus that are in competition for the same community niche (Human Microbiome Project, 2012; Walsh et al., 2015). Bacteriocins are ribosomally synthesized antimicrobial peptides (AMPs). They typically range in size from 20 to 60 amino acids and most often have a net positive charge (Hammami et al., 2010). Despite these similarities, bacteriocins have heterogeneous amino acid compositions. There has been much debate as to a system of classification for bacteriocins with researchers arguing as to whether there should be two, three, or even four classifications based on structural features and mode of action (Klaenhammer, 1993; Diep and Nes, 2002; Cotter et al., 2005; Heng et al., 2007; Perez et al., 2014). Broadly, bacteriocins are classified into two groups based on whether they are post-translationally modified (class I) or remain unchanged (class II) following synthesis. Although some broad-spectrum bacteriocins exist and have the potential for use in combating infections of unknown cause, potent narrow-spectrum bacteriocins have also been identified, allowing for the targeted control of pathogens without disrupting commensal populations (Cotter et al., 2013; Kommineni et al., 2015).

The ever-rising number of sequenced genomes of *Lactobacillus* species has established the consistent presence of bacteriocins within the genus. The system of production for certain bacteriocins has been described at least in part in many lactic acid bacteria, and the consensus elements required for bacteriocin production have been elucidated for class II. These include auto-induction via a signal peptide, a structural gene encoding a prepeptide characterized by a conserved N-terminal double-glycine leader sequence, and activation of the peptide by cleavage at a conserved location (Alvarez-Sieiro et al., 2016). Reports investigating two strains of the important probiotic species *L. acidophilus* have shed light on its bacteriocin lactacin B and its cognate inducer signal peptide that is produced in the presence of target bacteria (Dobson et al., 2007; Tabasco et al., 2009). However, the details of lactacin B delivery to its bacterial target have not been described beyond its presence in culture supernatant.

One possible mechanism for lactacin B delivery to target bacteria is through membrane vesicles (MVs). Reports have demonstrated that outer membrane vesicles (OMVs) of Gram-negative bacteria can function as delivery vehicles for DNA, virulence factors, antimicrobial metabolites (Schulz et al., 2018), and other cargo to host cells and bacteria in the environment (Bomberger et al., 2009; Bitto et al., 2017), with MVs isolated from Gram-positives thus far exhibiting similar characteristics (Lee et al., 2009). Previous proteomic analysis of *L. acidophilus* ATCC 53544 cells and MVs revealed an enrichment (50-fold increase compared to the whole cell) of bacteriocin pathway proteins in the MVs (Dean et al., 2019). Tabasco et al. had previously described that in response to induction with an autoinducer peptide, the lactacin B operon (*lab*) produced and released antimicrobials into the culture media capable of bactericidal activity in the closely related species *Lactobacillus delbrueckii* (Dobson et al., 2007; Tabasco et al., 2009). Based on our previous proteomic analyses and those described by Tabasco et al., we investigated the ability of *L. acidophilus* MVs to carry and deliver bacteriocin peptides to its niche competitor and reported opportunistic pathogen, *L. delbrueckii* (Darbro et al., 2009; Duprey et al., 2012). The results described here suggest that MVs could play a role in the gut microbiome as potential mechanisms of regulating bacterial populations in complex communities through targeted bactericidal activity. With this in mind, and with the potential for engineering MVs to control protein composition (surface and cargo), MVs of probiotic bacteria have the potential to emerge as a new platform for therapeutic delivery of community-control peptides or other cargo to populations within the gut.

## Results

### Examination of the Lactacin B Operon in *L. acidophilus* Strain ATCC 53544

Recently, we reported the complete genome of *L. acidophilus* ATCC 53544 and comparative analysis with the genome of *L. acidophilus* NCFM (Dean et al., 2017b). Alignment of the *lab* operons of the two strains identified a single missense mutation in the hypothetical protein CGZ81_00680/LBA1794 which results in a Pro178Thr change. Within the confines of these studies, CGZ81_00680 was not shown to be specifically enriched in the MVs and therefore will not be discussed further.

**FIGURE 1:**
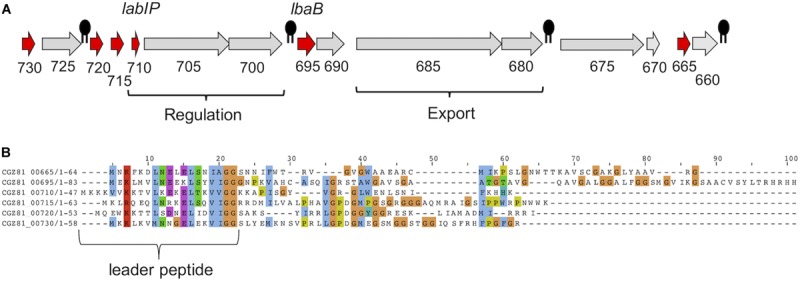
Putative bacteriocins in *L. acidophilus* ATCC 53544. **(A)** Gene organization of the lactacin B operon (*lab*) in *L. acidophilus* ATCC 53544. Abbreviated locus names are used for clarity; e.g., “730” is “CGZ81_00730.” Genes predicted to be bacteriocins are colored red. Putative intrinsic terminators are indicated by black symbols. Lactacin B regulation- and export-related genes are labeled; unlabeled genes are of unknown function. **(B)** Alignment of putative bacteriocin peptides [colored red in **(A)**] using Clustal Omega, with leader peptide indicated.

The *lab* operon is represented in Figure 1A, with putative bacteriocin-encoding genes [including the lactacin B inducer peptide (*LabIP*)] highlighted in red and with the location of *LabIP* and *lactacin B* labeled. For additional context, the location of Rho-independent transcription terminators predicted using ARNold^1^ (Naville et al., 2011) are also shown. Most of the unannotated genes within and surrounding the *lab* operon are predicted to encode cationic peptides or small proteins (<70 amino acids), consistent with the properties of many bacteriocins. To investigate whether the genes outside of those encoding peptides of known function (lactacin B and LabIP) were likely to produce bacteriocins, the amino acid sequences of these peptides were aligned against known antimicrobial peptides in the Antimicrobial Peptide Database (APD3)^2^ (Wang et al., 2016) and BActeriocin GEnome mining tooL (BAGEL4)^3^ (van Heel et al., 2018). This analysis identified genes outside of the *lab* operon (*LBA1791–LBA1803*) defined by Dobson et al. (2007) indicating an area of interest extending from *CGZ81_00665* to *CGZ81_00730*. According to our analysis, in addition to the *LabIP* and *lactacin B* genes of the previously defined operon, the products of the genes *CGZ81_00665*, *CGZ81_00715*, *CGZ81_00720*, and *CGZ81_00730* are predicted to encode bacteriocins. These results are consistent with the putative bacteriocin peptides produced by *L. acidophilus* NCFM listed by Kjos et al. (2010). Though there are no reports to date of LabIP having antimicrobial properties consistent with it being categorized as a bacteriocin, its identification as a putative bacteriocin by various models is not without precedent: Nisin, the well-studied auto-inducer peptide produced by *Lactococcus lactis*, also has known antimicrobial properties (Verbeke et al., 2017). For each of the genes examined, Table 1 reports the closest peptide in sequence similarity according to the APD3 along with relevant physicochemical characteristics calculated using the Peptides R package^4^ (Osorio et al., 2015).

**TABLE 1:** Putative *L. acidophilus* bacteriocin peptides.

**ATCC 53544 locus**	**NCFM locus**	**Propeptide sequence**	**MW (Da)**	**pI**	**ADP3**	**Percent identity**
CGZ81_00665	LBA1791	SNNIFWTRVGVGWAAEARCMIKPSLGNWTTKA VSCGAKGLYAAVRG	4900	10.6	Brevinin-2CG1	35%
CGZ81_00695	Lactacin B	GNPKVAHCASQIGRSTAWGAVSGAATGTAVGQA VGALGGALFGGSMGVIKGSAACVSYLTRHRHH	6275	10.9	Brochocin C	41%
CGZ81_00710	LabIP	KKAPISGYVGRGLWENLSNIFKHHK	2880	10.9	Dahlein 5.6	38%
CGZ81_00715	LBA1802	RRDMILVALPHAVGPDGMPGSGRGGGAQMRAI GSIPPWRPNWWK	4751	12.3	rtCATH_1	34%
CGZ81_00720	LBA1803	SAKSYIRRLGPDGGYGGRESKLIAMADMIRRRI	3695	11.4	Kenojeinin I	35%
CGZ81_00730	LBA1805	SLYEMKNSVPRLLGPDGMEGSMGGSTGGIQS FHFPGFGR	4259	9.3	rtCATH-1b	33%

To further investigate potential bacteriocins produced by ATCC 53544, computational analysis of the *lab* operon and the peptides it encodes was performed using Clustal Omega (Sievers and Higgins, 2018). Alignment of *lab* components from ATCC 53544 shows that each pre-peptide has relatively high sequence similarity of >30% identity (average = 42%) in the leader sequence, while the pro-peptide following cleavage at the double-glycine (GG) motif does not, with each at an identity <30% (average = 17%) (Figure 1B), where 30% is a generally cited threshold for identity (Pearson, 2013). This property of a conserved N-terminal leader sequence paired with a C-terminal sequence with very little, if any, sequence similarity is a common feature among bacteriocins (Drissi et al., 2015) and antimicrobial peptides in general.

### Identification of Bacteriocins Within MVs

Previously we reported that several *lab* operon members including CGZ81_00730 and LabIP were identified as being uniquely enriched in MVs relative to the cell (Dean et al., 2019). To expand upon this work, we sought to investigate the bactericidal activity related to the elevated concentration of *lab* operon products in the MVs of LabIP-induced cultures. As Tabasco et al. had reported previously, basal levels of bacteriocin were insufficient to induce cell-death in *L. delbrueckii*; we therefore chose to follow a similar protocol and induce over-expression of *lab* proteins through induction with the LabIP inducer (Tabasco et al., 2009). To ensure adequate activation and high levels of bacteriocin production we induced expression of all cultures with a 500 nM final concentration of the inducer peptide.

**FIGURE 2:**
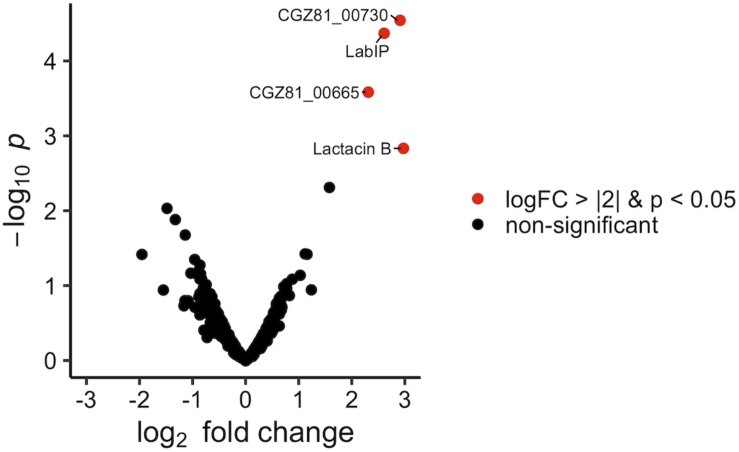
Protein enrichment within LabIP-induced MVs. Volcano plot of assigned MS spectra where each dot represents an identified protein. Fold change was calculated from average spectrum counts of LabIP-induced vs. uninduced and significance values were calculated. Red dots represent proteins with *p* < 0.05 and log2 fold change of < |2|. Black dots correspond to non-significant change between conditions by *p-*value or fold change.

The protein content of MVs purified from both LabIP-induced and non-induced *L. acidophilus* ATCC 53544 cultures was subjected to shotgun proteomic analysis. A total of 885 proteins were identified, among which 26 were unique to the non-induced MVs, 23 were unique to the LabIP-induced MVs, and 804 protein IDs were found in both samples (Supplementary Table S1). Calculation of the mean fold change in weighted spectral counts from non-induced and induced samples was used to identify those proteins that had the highest relative change in abundance in the MVs following induction with LabIP (Table 2). Out of the top ten most changed, six proteins are components of the *lab* operon: CGZ81_00665, LabIP, lactacin B, CGZ81_00715, CGZ81_00720, and CGZ81_00730. With the exception of CGZ81_00720, each had a mean fold change greater than 15. When fold change and *p*-values are visualized via volcano plot, four *lab* proteins stand out (Figure 2), and importantly, significantly more weighted spectral counts of the confirmed bacteriocin, lactacin B, were measured in the induced samples (*p* < 0.05; Student’s *t-*test).

**TABLE 2:** Identified proteins with the highest fold change between uninduced and induced samples.

**ATCC 53544** **locus**	**NCFM** **locus**	**Uninduced** **replicate 1**	**Uninduced** **replicate 2**	**Uninduced** **replicate 3**	**Induced** **replicate 1**	**Induced** **replicate 2**	**Induced** **replicate 3**	**Fold change** **replicate 1**	**Fold change** **replicate 2**	**Fold change** **replicate 2**	**Fold change** **average**
CGZ81_00695	Lactacin B	0.0	0.0	6.3	39.0	5.3	13.0	131.0	18.8	2.0	50.6
CGZ81_00710	LabIP	0.0	0.0	0.0	9.5	3.3	4.0	32.7	12.1	14.3	19.7
CGZ81_00665	LBA1791	0.0	0.0	0.0	9.0	2.7	2.3	31.0	9.9	8.8	16.6
CGZ81_00730	LBA1805	0.5	0.3	0.7	21.5	8.0	6.0	27.3	13.1	6.5	15.6
CGZ81_00715	LBA1802	0.0	0.3	0.3	13.0	0.0	0.7	44.3	0.5	1.5	15.4
CGZ81_03950	LBA0444	0.0	0.0	0.0	2.0	0.7	4.3	7.7	3.2	15.4	8.8
CGZ81_02665	LBA0222	0.0	0.0	0.7	0.5	4.7	0.7	2.7	16.6	1.0	6.7
CGZ81_00205	LBA1697	0.0	1.3	0.0	3.5	1.0	1.7	12.7	0.8	6.6	6.7
CGZ81_00720	LBA1803	0.0	0.0	0.0	4.0	0.3	0.7	14.3	2.1	3.2	6.6
CGZ81_07900	lepA	0.0	2.3	0.0	3.5	0.7	0.0	12.7	0.4	1.0	4.7

*Proteins sorted by highest mean fold change in MVs from cultures induced with LabIP, relative to non-induced. Values for each replicate represent weighted spectral counts. The complete list of proteins can be found in Supplementary Table S1.*

The presence of six *lab* proteins in the top ten most differentially loaded proteins in the MVs is significant when compared to the expected quantity resulting from a random process (*p* < 0.05; hypergeometric probability). This suggests that *lab* proteins are specifically enriched within MVs during conditions of *lab* upregulation. Overall, nine of the 15 *lab-*encoded proteins were not only identified in the MVs but also measured at a higher abundance in the induced samples compared to the non-induced control. None of the other proteins identified in the LabIP-induced samples were classified as bacteriocin or antimicrobial peptides allowing subsequent experiments to focus on the products of the *lab* operon.

**FIGURE 3:**
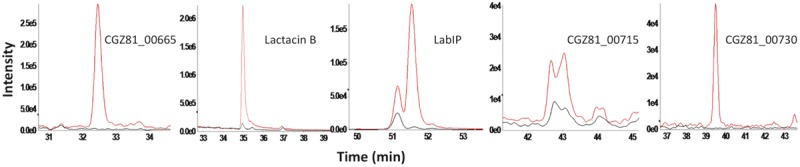
Targeted mass spectrometry of putative bacteriocins in MVs. Extracted ion chromatogram for each targeted precursor ion representing five most abundant proteins with from shotgun proteomics results. Peptide abundance was measured by the area of the peak. Chromatograms from LabIP-induced samples are colored red; uninduced are black.

For quantification that is more precise and to confirm the shotgun analysis, targeted mass spectrometry was performed on the five proteins with the highest mean fold change in the shotgun data (CGZ81_00665, lactacin B, LabIP, CGZ81_00715, and CGZ81_00730). Nine tryptic peptides from these five proteins of interest were selected for targeting. Peptide abundance was measured in the LabIP-induced and non-induced samples and is listed in Table 3 as area under the curve (AUC; shown in Figure 3). Out of the nine tryptic peptides targeted, six were detected in both samples (two distinct peptides from LabIP were detected). In the non-induced samples, the abundance of targeted peptides was substantially lower than in the LabIP induced samples or they were not detected at all. In contrast, clearly detectable peaks for each targeted peptide were found in LabIP-induced samples. Critically, in measuring the total ion abundance of the targeted peptides, these results confirm the significant increase in abundance of CGZ81_00665, lactacin B, LabIP, CGZ81_00715, and CGZ81_00730 in MVs following LabIP induction as determined via shotgun proteomics. The AUC of the peptide representative of lactacin B was 42-fold higher with LabIP-induction, a value similar to the shotgun results. Also of note, the LabIP itself was identified at increased levels following LabIP induction as would be expected for a Gram positive two-component quorum sense signaling system; however, the possibility of measuring exogenous LabIP bound to the purified MVs cannot be ruled out.

**TABLE 3:** Quantitative mass spectrometry of putative bacteriocins in MVs.

**ATCC 53544**	**NCFM**	**Targeted peptide**	**Precursor ion m/z (Da)**	**MW**	**Charge**	**Retention time**	**Uninduced**	**Induced**
**locus**	**locus**	**sequence**				**(min)**	**sample**	**sample**
CGZ81_00665	LBA1791	VGVGWAAEAR	508.27	1015.53	2	32.46	NA	4.64E+06
CGZ81_00695	Lactacin B	STAWGAVSGAATGTAVG QAVGALGGALFGGSMGVIK	1059.89	3176.63	3	32.97	5.07E+04	2.15E+06
CGZ81_00710	LabIP	APISGYVGR	460.25	918.48	2	27.67	NA	4.97E+06
		GLWENLSNIFK	660.88	1319.69	2	51.15	1.71E+06	3.40E+09
CGZ81_00715	LBA1802	RDMILVALPHAVGPDGM PGSGR	749.39	2245.15	3	NA	NA	NA
		DMILVALPHAVGPDGM PGSGR	697.36	2089.05	3	42.69	NA	5.57E+05
		AIGSIPPWRPNWWK	569.98	1706.89	3	NA	NA	NA
CGZ81_00730	LBA1805	LLGPDGMEGSMGGSTG GIQSFR	1077.5	2153.02	2	39.48	NA	8.14E+05
		RHFPGFGR	409.21	816.38	2	NA	NA	NA

*“NA” indicates that the tryptic peptide was not detected. Values for uninduced and induced samples are the calculated Area Under the Curve (AUC).*

### Growth Inhibition and Pore Formation in *L. delbrueckii* by Membrane Vesicles

The presence of bacteriocins within MVs does not necessarily ensure bactericidal activity as MV localization or packaging can often be the product of over-expression and passive encapsulation of abundant cytoplasmic proteins within nascent MVs. We therefore employed a number of conventional cell viability assays to assess the antimicrobial activity of our LabIP-induced MVs.

**FIGURE 4:**
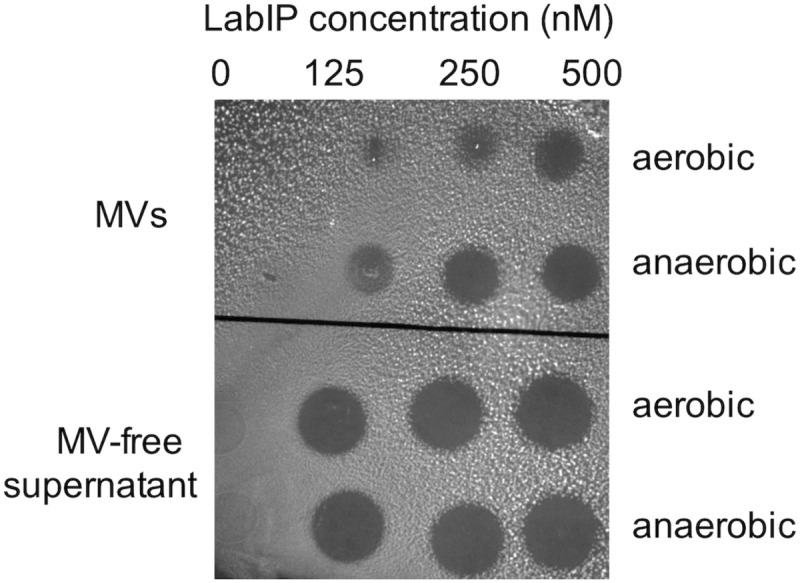
Bacteriocin production under different conditions. Spot-on-lawn assays for bacteriocin production associated with MVs (top) or free in the supernatant (bottom) from cultures grown under aerobic or anaerobic conditions and induced with a range of LabIP concentrations, from non-induced (“0” at the left) to a maximum concentration of 500 nM at the right. *L. delbrueckii* subsp. *lactis* ATCC 15808 was used as the bacteriocin indicator strain; lawns were grown on MRS agar under anaerobic conditions. A representative image is shown.

MVs were prepared from cultures treated with a range of LabIP concentrations and under both aerobic and anaerobic conditions. From these preparations, MVs were diluted to equivalent concentrations based on quantitation of particles/mL as determined using nanoparticle tracking instrumentation and software. Bacteriocin-containing MVs and non-induced control samples were spotted directly onto a lawn of the sensitive indicator strain, *L. delbrueckii* subsp. *lactis* ATCC 15808. Under the conditions in this study, 125 nM was the minimum LabIP concentration necessary for observable bacteriocin activity of the MVs (representative plate shown in Figure 4). As expected, the incubation with increasing LabIP concentrations corresponded to increased clearing of *L. delbrueckii* growth. Under the conditions of these studies, MVs isolated from LabIP-induced cultures grown under anaerobic conditions appeared to have slightly increased the bacteriocin activity compared to those isolated from aerobically grown cultures. No inhibitory activity was observed for MVs from cultures that were not induced with LabIP suggesting that the bacteriocin concentration within these particles was negligible and unable to inhibit growth of *L. delbrueckii.*

**FIGURE 5:**
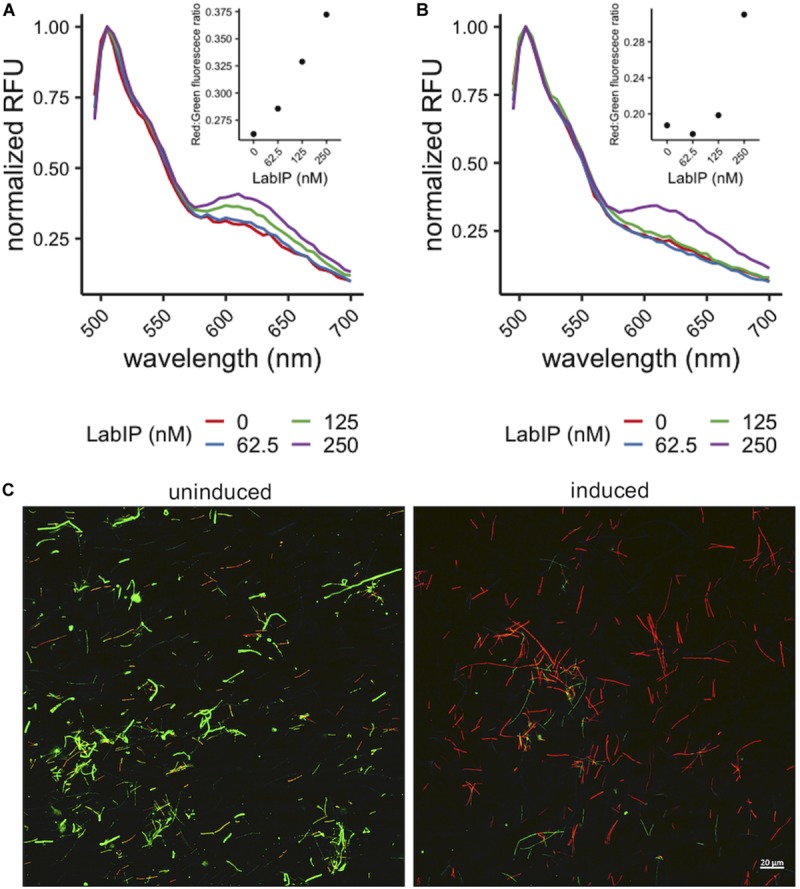
Pore formation caused by MV-associated bacteriocins. Increase in red wavelength fluorescence from LIVE/DEAD stain indicates peptides in the LabIP-induced *L. acidophilus* ATCC 53544 supernatant **(A)** and those associated with MVs **(B)** create pores in the *L. delbrueckii* cell wall and/or membrane. **(C)** Representative fluorescence microscopy images of LIVE/DEAD-stained *L. delbrueckii* show an increase in red-fluorescing cells after incubation with MVs from LabIP-induced *L. acidophilus*, while *L. delbrueckii* treated with MVs from non-induced cultures show a minimal number of red-fluorescing cells.

While many bacteriocins are believed to function through membrane pore formation, MV-mediated delivery of bacteriocins to target cells has not previously been described and we could not ensure that a similar mechanism of antimicrobial activity was occurring. To assess membrane integrity in *L. delbrueckii* subsequent to MV exposure we employed a conventional live/dead staining assay using propidium iodide and SYTO9 stains to quantitate the pore formation and potential bactericidal activity of LabIP-induced culture media and purified MVs. In these experiments, a fluorescence shift from green (live cells) to red (dead cells) can be monitored as a measure of the pore-forming activity of the *L. acidophilus* bacteriocins. In a fluorescent plate assay, we observed an increase in red wavelength fluorescence relative to green fluorescence indicating that peptides in both the culture media and the purified MVs from LabIP-induced cultures compromise the *L. delbrueckii* cell wall and/or membrane (Figures 5A,B). These samples were also examined using confocal microscopy to visualize the results of LabIP-induced MV exposure (Figure 5C). Images were taken using both green and red filters of *L. delbrueckii* treated with MVs from either non-induced or LabIP-induced *L. acidophilus* cultures. Red:green ratios for the two conditions differed by ∼19-fold, quantitatively confirming the significant increase of visible red cells (*p* < 0.05; Student’s *t*-test).

### Active Bacteriocin Is Stably Contained Within MVs

In previous work, Tabasco et al. (2009) found that a portion of the lactacin B produced remained adhered to the cell wall following secretion. As it pertains to MV localization, this leads to the question of whether lactacin B and others are externally facing or contained within the lumen of the proteoliposomes. As shown in Figure 1B, each of the putative bacteriocins of the *lab* operon contains several cleavage sites for either proteinase K and/or trypsin protease. As the MV lipid bilayer is impenetrable to proteases, only peptides displayed on the exterior of MVs are accessible, allowing for a simple loss of function assay to be developed. This method has been employed by others to assess the localization of nanoluciferase in *Escherichia coli* OMVs and was modified here to determine bacteriocin location in MVs (Chen et al., 2017).

**FIGURE 6:**
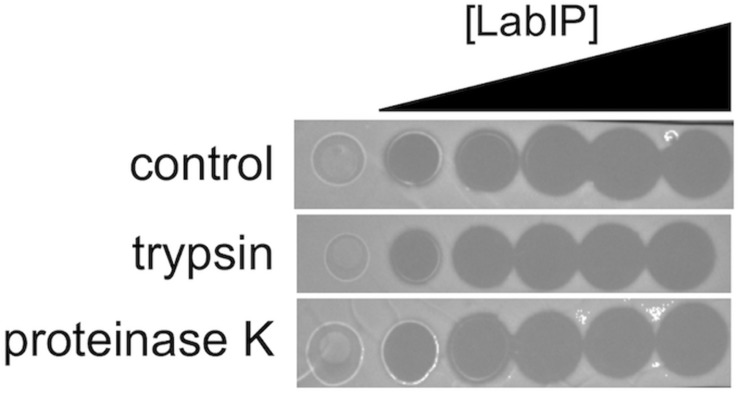
Localization and stability of MV-associated bacteriocins. A protease accessibility assay was used to evaluate the localization of bacteriocin. MVs were treated with trypsin or proteinase K and then evaluated by spot-on-lawn assay.

**FIGURE 7:**
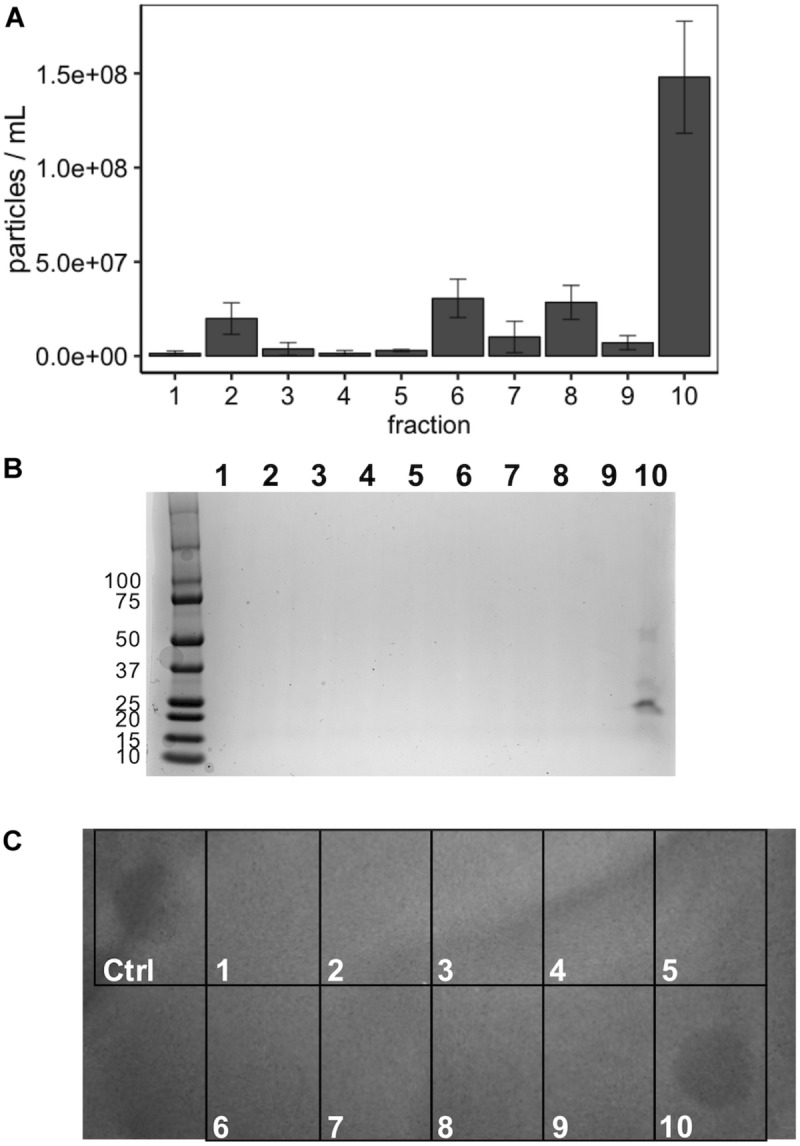
Optiprep purification of MVs. **(A)** Following fractionation MV concentration (particles/mL) was determined using NanoSight from 1:100 diluted fractions in PBS. **(B)** Coomassie stain of SDS-PAGE gel of the 10 Optiprep fractions. **(C)** Spot-on-lawn of *L. delbrueckii* following Optiprep fractionation; control (ctrl) is the input MVs prior to fractionation.

Incubation of MVs with 1 mg/mL of proteinase K and trypsin did not have an observable impact on the subsequent growth inhibition facilitated by the LabIP-induced MVs (Figure 6). This suggests that although some MV-associated bacteriocin may be externally localized and accessible to proteases, a sufficient amount is contained within the lumen or embedded deep in the membrane of the MV and retains bactericidal activity. To further examine whether free or loosely associated bacteriocin may be responsible for the growth inhibitory activity of the MVs, we used gradient ultracentrifugation to ensure the removal of peptides and proteins not associated with the MVs. Individual fractions collected post-centrifugation were tested in spot-lawn assays as previously described to assess antimicrobial activity. Activity was only observed in the fraction containing MVs, while no activity was observed in gradient fractions corresponding to free bacteriocin (Figure 7). The active fraction was examined using particle tracking analysis to ensure the presence of MVs.

**FIGURE 8:**
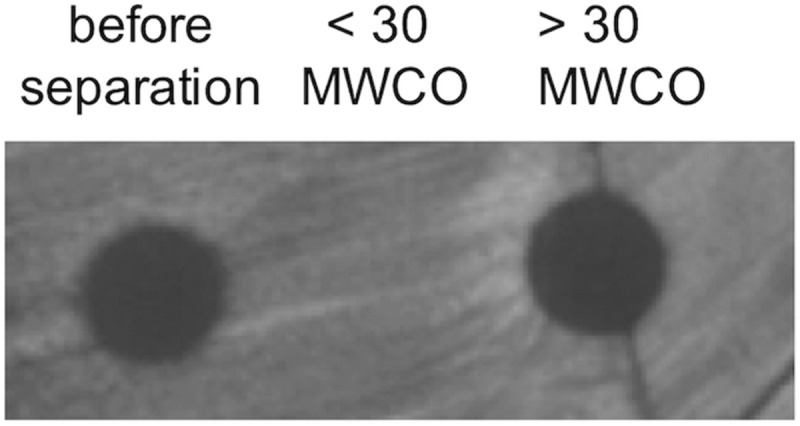
Sequestration of bacteriocins within MVs. The stable maintenance of bacteriocin peptide within MVs was determined by weeklong incubation followed by separation by MWCO filtration. Fractions were tested for bacteriocin activity using spot-on-lawn assays. Images are representative of triplicate experiments.

Given the protective barrier provided by the encapsulating lipid membrane, MVs have the potential to serve as long-range delivery vehicles shielding contents from environmental proteases or inactivation molecules that would be prevalent in a microbial community. As MVs contain membrane proteins, channels, and pores identical to those in the parental bacterium, it was necessary to assess whether bacteriocins remained within the MVs or gradually diffused across the membrane into the surrounding environment. Here we resuspended purified MVs in phosphate buffered saline (PBS) and stored them at 4°C for 7 days. MVs were then separated from released peptides and other biomolecules using 30,000 MWCO spin concentrators and the manufacturer’s protocol (Pierce). The retained MVs were recovered and the volume adjusted to 1 mL with PBS, and bacteriocin activity was assessed using spot-on-lawn assays targeting *L. delbrueckii*. The recovered MVs, filtrate, and solubilized filtride (material remaining on the filter) were compared in parallel (Figure 8). The results of these studies indicate that the bacteriocin and bactericidal activity remains associated with the MV fraction.

### Membrane Fusion of *L. acidophilus* MVs and *L. delbrueckii*

**FIGURE 9:**
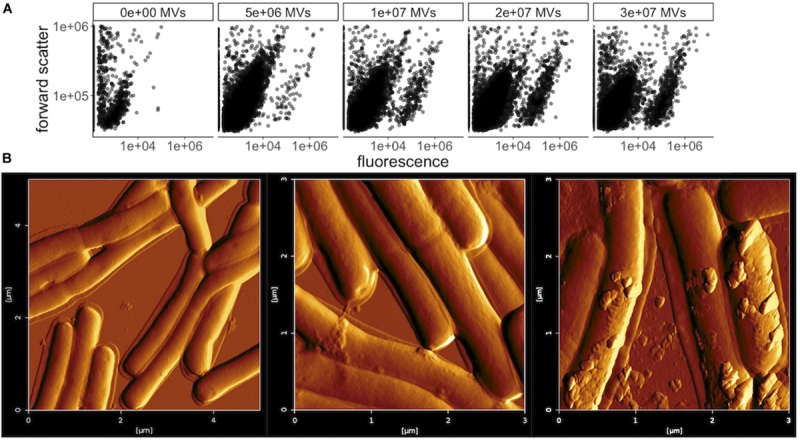
Membrane fusion of MVs with *L. delbrueckii*. **(A)** Flow cytometry plots of forward scatter over FL1 fluorescence (533/30 nm) showing *L. delbrueckii* incubated with increasing amounts of DiO-labeled MVs purified from LabIP-induced *L. acidophilus* cultures. **(B)** Atomic force microscopy (AFM) images of *L. delbrueckii* incubated with increasing amounts (from left to right: no MVs, 4 × 10^6^ or 8 × 10^6^) of MVs purified from LabIP-induced *L. acidophilus* cultures.

MVs secreted by several bacterial species, including Gram-positive *Bacillus subtilis*, have previously been shown to fuse with the cell membrane of other species, as a possible delivery mechanism for the contents of the MV lumen (Kim et al., 2016). To help determine the mechanism of the bacteriocin delivery that leads to growth inhibition of *L. delbrueckii* by *L. acidophilus* MVs, membrane fusion was investigated by DiO [DiOC_18_(3) (3,3-dioctadecyloxacarbocyanine perchlorate] staining. DiO was used previously for tracking of *E. coli* and *Pseudomonas aeruginosa* OMV internalization into cells (Perez-Cruz et al., 2016; Alves et al., 2017; Bitto et al., 2017), by binding specifically to MV membrane lipids, which are transferred to the target cell upon fusion. *L. acidophilus* MVs were labeled with DiO and incubated with *L. delbrueckii* or *E. coli* cells, and flow cytometry was used to measure shifts in cell size and fluorescence. Both *L. delbrueckii* and *E. coli* cells displayed a dose-dependent shift in fluorescence with addition of *L. acidophilus* MVs from LabIP-induced cultures (Figure 9A and Supplementary Figure S1), suggesting fusion and incorporation of DiO from *L. acidophilus* MVs into the membrane of *L. delbrueckii*, where the contents of the MVs are likely transferred into the cell.

MV association and fusion to *L. delbrueckii* was also visualized by atomic force microscopy (AFM). A range of concentrations of purified MVs were incubated with washed, glutaraldehyde-fixed *L. delbrueckii* cells overnight, after which the mixtures were analyzed with AFM. After incubation with either 0, 4 × 10^6^, or 8 × 10^6^ MVs as determined by NanoSight, the MVs were observed to adhere to the membrane of *L. delbrueckii* (Figure 9B). Interestingly, in the sample using 4 × 10^6^ MVs all observable vesicles were found to be cell-associated, supporting the MV-cell association indicated by the fluorescent shift seen in the membrane fusion results above. Altogether, these data support a possible mechanism of MV-cell fusion for the delivery of bacteriocin to *L. delbrueckii.*

## Discussion

In this study, a known bacteriocin was found to be contained within secreted MVs from *L. acidophilus* that are capable of killing a potential member of its GI niche-competition and opportunistic pathogen *L. delbrueckii.* Bacteriocins produced by lactic acid bacteria are of particular interest due to the safe use and FDA approval of Nisin (Mokoena, 2017) and the Generally Regarded As Safe (GRAS) and Qualified Presumption of Safety (QPS) statuses that several lactic acid bacteria possess (Alvarez-Sieiro et al., 2016). However, the role of bacteriocins in the function of commercial probiotics is unknown. One recent study investigating the composition of current commercial probiotics found that the only bacteriocin-producing organisms identified were lactacin B-producing *L. acidophilus* strains (Hegarty et al., 2017), highlighting the importance of understanding the function and delivery of this specific bacteriocin. Bacteriocins are also of interest clinically. In one recent report, Kommineni et al. (2015) demonstrated that bacteriocins produced by commensals that occupy a specific niche in the GI tract are capable of specifically eliminating intestinal colonization by MDR bacteria, killing target the pathogen without significant disruption of the commensal microbiota. Despite promising results, the introduction of bacteriocins into the biomedical industry and their further development in food applications have been impeded due to the lack of reliable delivery systems (Arthur et al., 2014).

The use of MVs to deliver cargo is a field of research that has recently reported some success. In one such study using MVs as a delivery device for an anti-cancer therapy, Gujrati et al. (2014) displayed an anti-HER2 antibody on an OMV and showed that the engineered OMV could specifically target and eliminate tumors. Beyond this example, other proteins and peptides have been engineered to be packaged or displayed on bacterial OMVs and demonstrate their potential in the development of future therapeutics (Dean et al., 2017a). Although lactobacilli have not yet been engineered to produce MVs containing a specific cargo, their widespread, ubiquitous use as probiotics and their GRAS status has increased interest in the biotechnological potential of lactobacilli including exploration through comparative genomics of 213 strains and associated genera (Sun et al., 2015) and success in large trials of probiotics (Panigrahi et al., 2017). In addition, the probiotic characteristics of the genera have led to continuous effort in engineering, where lactobacilli have been engineered to deliver factors other than bacteriocins, such as elafin, superoxide dismutase, and pro-insulin to target cells (Bermudez-Humaran et al., 2013).

In our previous efforts and those described here, we have shown that *L. acidophilus* is capable to enriching its MVs with bactericidal peptides able to inhibit the growth and proliferation of *L. delbrueckii* (Dean et al., 2019). Here we showed that this phenomenon was specifically associated with the induction of the *lab* operon via the LabIP peptide which was added exogenously. While several of the putative bacteriocins were detected in the non-induced samples, as shown here and also by Tabasco et al. (2009), basal levels of these peptides and proteins are insufficient to induce bactericidal activity. Therefore, MV packaging and concentrations of the *lab-*associated bacteriocins likely occurs in response to an over-expression of these proteins as we showed here using a number of different growth assays and pore formation studies. The studies performed here will be foundational for future efforts in the development of *L. acidophilus* MVs as potential delivery vehicles for natural and recombinant molecules to other cells within a mixed community.

In light of the recent discovery of MVs produced by lactic acid bacteria, the potential use of both natural and engineered MVs in food and biomedical applications has been proposed (Liu et al., 2018). However, certain research questions remain. Are currently used probiotic strains of lactacin B-producing *L. acidophilus* providing MVs that kill *L. delbrueckii* and other sensitive species *in vivo* in the GI tract? The results of Hegarty et al. (2017) suggest that only lactacin B-producing *L. acidophilus* strains are used in commercial probiotics. It is unclear whether this is coincidental or the result of lactacin B production being a favorable probiotic characteristic. Currently it is unknown whether bacteriocins of other *L. acidophilus* strains such as acidocin CH5 (Chumchalova et al., 2004) and acidocin J1132 (Tahara et al., 1996) are also naturally found within MVs when their production is initiated. Outside of the *lab* operon, no other putative bacteriocins were identified in the present study. Future work can determine whether any specific signal sequence or mechanism is required for peptide packaging into MVs in *L. acidophilus*. Although some sequence similarity was observed among the putative bacteriocins found in the MVs, which may suggest the involvement of that sequence in peptide packaging, it is possible that local concentration effects in the cytoplasm at the point of MV formation may be responsible for non-specific loading into the vesicles. Previous studies looking at Gram-negative OMVs have shown their ability to enter eukaryotic cells and deliver their protein contents, as well as LPS and other biomolecules, into host cells (Bomberger et al., 2009; Kaparakis-Liaskos and Ferrero, 2015; Bitto et al., 2017). It is unclear whether MVs of the Gram-positive *L. acidophilus* would behave similarly. Our results show that the purified *L. acidophilus* MVs fuse with both *L. delbrueckii* and *E. coli*, suggesting non-specific binding with bacterial cells.

The identification of bacteriocin pathway components as enriched proteins in the MVs produced by *L. acidophilus* (Dean et al., 2019) provided the impetus to explore the relationship between bacteriocins and MVs. In this study, we investigated the ability of *L. acidophilus* MVs to carry and deliver putative bacteriocin peptide to a target organism. While we did show bactericidal activity with purified, LabIP-induced MVs, due to the presence of other putative bacteriocins including CGZ81_00730, we cannot conclude that lactacin B was the only bacteriocin responsible for the observed activity targeting *L. delbrueckii.* In previous work by Dobson et al., synthetically produced LabIP, LBA1801, LBA1802, and LBA1803 failed to exert antimicrobial activity against *L. delbrueckii* (Dobson et al., 2007). Therefore, among the putative bacteriocins of the *lab* operon identified both computationally and by shotgun proteomics of MVs, only LBA1791 (CGZ81_00665) and LBA1805 (CGZ81_00730) remained possible candidates for antimicrobial peptides against *L. delbrueckii* aside from lactacin B. Analysis of the *lab* operon, however, does not identify the requisite immunity proteins that would indicate the presence of multiple bacteriocins within this operon. It is therefore possible that each of the other putative bacteriocins within this operon are non-functional. In subsequent studies, we anticipate examining each of these individually through the construction of deletion mutants for each of the bacteriocins of the *lab* operon. Our results do, however, suggest that MVs produced by probiotic bacteria may be an interesting platform for therapeutic delivery of community-control peptides or other cargo, and that MVs may play a role in the gut microbiome niche as a mechanism for elimination or inhibition of competing bacteria.

## Materials and Methods

### Bacterial Strains

*L. acidophilus* (ATCC 53544; Dean et al., 2017b) was grown in de Man, Rogosa, Sharpe (MRS) broth under both aerobic and anaerobic conditions. Anaerobic conditions were maintained using AnaeroGen anaerobic atmosphere generation bags (Fluka, St. Louis, MO, United States) in AnaeroJar jars (Thermo Fisher Scientific, Hampton, NH, United States). Oxygen level (< 1%) was monitored using anaerobic indicator strips (Thermo Fisher Scientific, Hampton, NH, United States). Lactacin B indicator strain *L. delbrueckii* subsp. *lactis* ATCC 15808 was grown in MRS under anaerobic conditions in AnaeroJar jars at 37°C. Agar media was prepared by adding 1.5% (w/v) agar to liquid broth. *E. coli* BL21 (DE3) were grown in LB broth with shaking at 37°C.

### Membrane Vesicle Purification

The procedure for purification of MVs from *L. acidophilus* culture supernatants was similar to methods previously described for OMV purification from *E. coli* (Alves et al., 2015), with some modifications. Briefly, 40 mL cultures were grown overnight statically at 37°C. After the cells were pelleted twice at 5000 × g for 30 min, the supernatant fraction was filtered at 0.45 μm. The filtrate was ultracentrifuged at 129,000 × g for 1.5 h in a Sorvall WX Ultra 90 centrifuge using an AH-629 rotor (Thermo Fisher Scientific, Rockford, IL, United States), the supernatant was decanted or collected for use as an MV-free control in experiments, and the MV pellet was incubated overnight at 4°C in PBS. The final solubilization step concentrated the MVs 36-fold in PBS.

OptiPrep purification (gradient ultracentrifugation) was performed as previously described by Kieselbach and Oscarsson (Chen et al., 2013). To separate the purified MVs from loosely associated peptides and proteins they were purified using OptiPrep medium (Progen Biotechnik GmbH, Heidelberg, Germany). MV pellets were resuspended in 150 μL PBS – OptiPrep mixture (45% v/v) placed at the bottom of a 5 mL ultracentrifuge tube and sequentially covered with decreasing density layers (35, 30, 25, 20, 15, and 10%). The gradient was ultracentrifuged at 236,000 × g for 3 h in a Sorvall WX Ultra 90 centrifuge using an AH-650 rotor (Thermo Fisher Scientific, Rockford, IL, United States), following which ten equal-volume (500 μL) were sequentially removed. The fractions were assessed for MV concentration using NanoSight, protein composition using Coomassie stained SDS-PAGE, and growth inhibitory activity by spot-on-lawn assay (described below).

### NanoSight

Vesicle count as well as size, volume, and surface area distributions were obtained on a NanoSight LM10 system (Malvern Instruments Ltd., Worcestershire, United Kingdom) using NTA 2.3 Nanoparticle Tracking and Analysis software. Samples were diluted 1:100 or 1:1000 in pH 7.4 PBS with camera shutter and gain optimized for data collection. Videos (90 s) were taken and frame sequences were analyzed under auto particle detection and tracking parameters: detection threshold, pixel blur, minimum track length, and minimum expected particle size. All samples were run at RT and allowed to equilibrate prior to analysis. NanoSight counting was utilized for normalization required for experiments in the study. Output concentration in particles/mL was used for all experiments in this study.

### Bacteriocin Production Assays

For assessing bacteriocin production, cultures of *L. acidophilus* were grown overnight with varying concentrations of LabIP peptide, sequence: KKAPISGYVGRGLWENLSNIFKHHK (GenScript, Piscataway, NJ, United States). OD 600 nm was checked to assure equal growth. From these cultures, MVs were purified (as described above). Bacteriocin growth inhibitory activity was assayed using an adaptation of the spot-on-lawn assay for detection of bacteriocins (Lewus and Montville, 1991). From an overnight culture of *L. delbrueckii*, 150 μL was spread on to MRS agar plates and allowed to air dry. After drying, 4 μL of solution (either buffer, purified MVs (10^10^ particles/mL), or supernatant) was spotted onto the plates. The plates were incubated at 37°C overnight (∼19–24 h) and any zones of inhibition indicating antagonistic activity of bacteriocin were recorded.

Protease accessibility assays on MVs was performed using trypsin and proteinase K. Three biological replicates of purified MVs solubilized in PBS were incubated with 1 mg/mL of trypsin and proteinase K at 37°C for 1 h as previously described (Richins et al., 1997). Following incubation, MVs were plated on a *L. delbrueckii* lawn and zones of inhibition of indicator strain growth were recorded.

Time-dependent stability of MVs in buffer was carried out using three biological replicates of purified MVs solubilized in PBS and incubated at 4°C for 7 days. Following incubation, MVs were centrifuged in Pierce Concentrators, PES, 30K MWCO (Thermo Fisher Scientific, Rockford, IL, United States). MVs prior to filtration, the filtrate, and solubilized filtride (material remaining on the filter) were plated on a *L. delbrueckii* lawn.

### Confocal Microscopy

LIVE/DEAD-stained *L. delbrueckii* was prepared by incubating an overnight culture of *L. delbrueckii* with SYTO9 and propidium iodide (LIVE/DEAD BacLight Bacterial Viability Kit, Thermo Scientific, Rockford, IL, United States) following centrifugation and resuspension in phosphate buffer to a concentration 10^8^ cells/mL. This preparation was incubated with MVs purified from *L. acidophilus* cultures induced with LabIP (described in section Materials and Methods sections above) overnight at RT. A suspension of 10 μL of labeled cells were pipetted onto 20 × 20 mm glass coverslips (VWR, Radnor, PA, United States). The cover glass was inverted onto precleaned microscope slides and sealed with acrylic nail polish. Confocal images were taken with a Zeiss AxioObserver.Z1/7 LSM 800 Airyscan confocal microscope with a Plan-Apochromat 20x/0.8 M27 objective. SYTO9 and propidium iodide fluorescence was excited at 488 nm: 0.20% laser power and 561 nm: 0.20% laser power, respectively. The emission spectra of SYTO9 was collected with 490–550 nm filters and detected with the LSM 800 Airyscan detector. PI emission spectra was collected with 550–700 nm filters and detected with the LSM 800 GaAsP-Pmt2 detector. Each image was taken with a 2.06 μs pixel dwell, 10.13 s scan time per frame with 4x averaging. Images are maximum intensity projections composed of 6 optical sections over a 4.7 μm Z-stack interval in a 319.45 μm × 319.45 μm field of view. Images were collected using the Zeiss Zen Blue imaging software (Carl Zeiss, LLC, Thornwood, NY, United States).

To analyze the collected images, the ratio of red:green fluorescing cells was calculated using ImageJ (Schneider et al., 2012), where objects larger than 50 × 50 pixels were counted following conversion of green and red-channel images to binary 16-bit grayscale images with background subtraction, and the red:green ratio of cells was calculated.

### Atomic Force Microscopy Measurements

*L. delbrueckii* used for AFM were grown overnight and fixed for 10 min with 0.25% glutaraldehyde. The cells were then pelleted and washed three times with water. The fixed cells were then incubated overnight without or with ∼4 × 10^6^ or ∼8 × 10^6^ *L. acidophilus* MVs. Samples were dried onto mica discs mounted on standard glass microscope slides and rinsed by immersion in deionized water to remove salts, then dried again. Samples were imaged using a JPK NanoWizard 4a AFM equipped with a Tap300Al-G tip in AC mode, recording height, lock-in phase, and lock-in amplitude channels. Image processing was performed using JPK Data Processing Software.

### Flow Cytometry

*L. acidophilus* MVs were stained by incubation with 5 μM DiO [DiOC_18_(3) (3,3-Dioctadecyloxacarbocyanine Perchlorate], Thermo Fisher Scientific, Waltham, MA, United States) for 30 min, subsequently washed on a 30K MWCO membrane with phosphate buffer to remove unbound dye and resuspended to equal the starting volume. Dyed MVs were then incubated in a range of concentrations with either *L. delbrueckii* or *E. coli* cells and incubated overnight. Cell-MV mixtures were run on an Accuri C6 Cytometer (BD Biosciences, Franklin Lakes, NJ, United States) and analyzed using forward scatter and the FL1 detector (533/30 nm).

### Mass SPECTROMETRY

#### Sample Preparation

Following incubation with 500 nM LabIP, MVs were purified from cultures of *L. acidophilus* and uninduced control. Triplicate biological samples of MV pellets were solubilized in 10% n-propanol in 50 mM ammonium bicarbonate in a 10 mL suspension. Samples were normalized by total protein content prior to digestion using the Pierce BCA Protein Assay Kit (Thermo Fisher Scientific, Rockford, IL, United States). Fifty micrograms of protein was digested with sequencing-grade modified trypsin (Promega, Madison, WI, United States) at a ratio of 50:1 in a Barocycler (HUB 440-SW16, Pressure Biosciences Inc., Easton, MA, United States) at 45 kpsi for 50 s, then no pressure for 10 s, for 60 cycles at room temperature. Digested samples (150 μL) were evaporated via speed-vac then reconstituted in 0.1% formic acid in water (solvent A).

#### Liquid Chromatography Tandem Mass Spectrometry (LC-MS/MS)

Shotgun LC-MS/MS was performed on a Thermo Scientific Orbitrap Fusion Lumos equipped with a Nanospray Flex Ion Source in data-dependent acquisition mode. Peptides (3 μg) were loaded onto a reversed-phase C18 column by a Dionex Ultimate WPS3000 autosampler. A Dionex Ultimate 3000 RSLCnano system separated peptides across a 90 min gradient of 2–60% solvent B (0.1% formic acid in acetonitrile) at a flow rate of 300 nL/min. Peptide samples were further analyzed using a Sciex Triple TOF 5600 coupled to an eksigent HPLC chromatography system, essentially as described previously (Schultzhaus et al., 2019). To target peptides from specific proteins of interest, the AnalystTF software (version 1.7.1) was set to preferentially fragment peptide ions that eluted across a 40-min gradient from 5% to 40% acetonitrile. Mass spectrometry data were searched using Mascot (version 2.6.1, Matrix Science, London, United Kingdom) and X!Tandem. Peptide-spectrum matches were validated in Scaffold (version 4.8.2, Proteome Science Inc., Portland, OR, United States). The fragment ion mass tolerance was ±0.60 Da and the parent ion tolerance was ±0.60 Da. Proteins identified by ≥2 peptides (protein probability 80%, peptide probability 95%) were used.

Mass spectrometry data were deposited in the ProteomeXchange Consortium via the PRIDE partner repository with the dataset identifier PXD012967 and 10.6019/PXD012967.

### Computational Analysis

Clustal Omega was used to align predicted protein and DNA sequences (Sievers and Higgins, 2018). Alignment images were generated using JalView (Waterhouse et al., 2009). Rho-independent transcription terminators were predicted using ARNold (Naville et al., 2011). For peptide analysis, cleaved sequences were aligned against confirmed antimicrobial peptides found in the Antimicrobial Peptide Database 3 (APD3) (Wang et al., 2016) and physicochemical characteristics of the peptides were determined using the Peptides R package (Osorio et al., 2015). The limma R package was used to perform the two-sample *t*-tests using an empirical Bayes method to adjust the estimate of variance of each protein according the protocol described by Kammers et al. (2015) and Ritchie et al. (2015), the output of which was each protein with its log_2_ fold-change and moderated *p-*value corresponding to the moderated *t*-statistic, which were used to create the volcano plots.

## Data Availability Statement

The datasets generated for this study can be found in the mass spectrometry proteomics data have been deposited to the ProteomeXchange Consortium via the PRIDE partner repository with the dataset identifier PXD012967 and 10.6019/PXD012967.

## Author Contributions

SD, JC, and SW designed experiments and were responsible for microbial handling, plate-based assays, growth inhibition studies, and all steps of MV preparation and characterization. DP was responsible for confocal microscopy and analysis. MR, DL, and WH were responsible for mass spectrometry analysis and interpretation of results for all of MV materials. All authors contributed equally to the preparation and editing of this manuscript.

## Conflict of Interest

The authors declare that the research was conducted in the absence of any commercial or financial relationships that could be construed as a potential conflict of interest.

## Footnotes

1 http://rna.igmors.u-psud.fr/toolbox/arnold/

2 http://aps.unmc.edu/AP/

3 bagel4.molgenrug.nl

4 https://cran.r-project.org/package=Peptides
