# Supplementary material for: Lactobacillus acidophilus Membrane Vesicles as a Vehicle of Bacteriocin Delivery
Source: Front Microbiol. 2020 Apr 30;11:710. doi: 10.3389/fmicb.2020.00710 (PMC7203471; doi:10.3389/fmicb.2020.00710)
Supplement: Supplementary file 1 [file Table_1.DOCX]

**Table S1. Shotgun proteomics.** Proteins are sorted by highest mean fold change in MVs from cultures induced with LabIP, relative to non-induced. Values for each replicate represent weighted spectral counts.

| ATCC 53544 locus | NCFM locus | Uninduced replicate 1 | Uninduced replicate 2 | Uninduced replicate 3 | Induced replicate 1 | Induced replicate 2 | Induced replicate 3 | Fold change replicate 1 | Fold change replicate 2 | Fold change replicate 3 | Fold change average |
| --- | --- | --- | --- | --- | --- | --- | --- | --- | --- | --- | --- |
| CGZ81_00695 | Lactacin B | 0.0 | 0.0 | 6.3 | 39.0 | 5.3 | 13.0 | 131.0 | 18.8 | 2.0 | 50.6 |
| CGZ81_00710 | LabIP | 0.0 | 0.0 | 0.0 | 9.5 | 3.3 | 4.0 | 32.7 | 12.1 | 14.3 | 19.7 |
| CGZ81_00665 | LBA1791 | 0.0 | 0.0 | 0.0 | 9.0 | 2.7 | 2.3 | 31.0 | 9.9 | 8.8 | 16.6 |
| CGZ81_00730 | LBA1805 | 0.5 | 0.3 | 0.7 | 21.5 | 8.0 | 6.0 | 27.3 | 13.1 | 6.5 | 15.6 |
| CGZ81_00715 | LBA1802 | 0.0 | 0.3 | 0.3 | 13.0 | 0.0 | 0.7 | 44.3 | 0.5 | 1.5 | 15.4 |
| CGZ81_03950 | LBA0444 | 0.0 | 0.0 | 0.0 | 2.0 | 0.7 | 4.3 | 7.7 | 3.2 | 15.4 | 8.8 |
| CGZ81_02665 | LBA0222 | 0.0 | 0.0 | 0.7 | 0.5 | 4.7 | 0.7 | 2.7 | 16.6 | 1.0 | 6.7 |
| CGZ81_00205 | LBA1697 | 0.0 | 1.3 | 0.0 | 3.5 | 1.0 | 1.7 | 12.7 | 0.8 | 6.6 | 6.7 |
| CGZ81_00720 | LBA1803 | 0.0 | 0.0 | 0.0 | 4.0 | 0.3 | 0.7 | 14.3 | 2.1 | 3.2 | 6.6 |
| CGZ81_07900 | lepA | 0.0 | 2.3 | 0.0 | 3.5 | 0.7 | 0.0 | 12.7 | 0.4 | 1.0 | 4.7 |
| CGZ81_08900 | galM | 0.0 | 0.0 | 0.0 | 2.5 | 0.3 | 0.3 | 9.3 | 2.1 | 2.1 | 4.5 |
| CGZ81_07890 | recJ | 0.0 | 1.7 | 0.3 | 3.0 | 0.7 | 0.3 | 11.0 | 0.5 | 1.0 | 4.2 |
| CGZ81_06650 | hslU | 3.0 | 2.0 | 0.0 | 4.0 | 3.0 | 2.3 | 1.3 | 1.4 | 8.8 | 3.8 |
| CGZ81_05935 | LBA0837 | 0.0 | 0.0 | 0.0 | 1.5 | 1.0 | 0.0 | 6.0 | 4.3 | 1.0 | 3.8 |
| CGZ81_02620 | metG | 0.0 | 0.0 | 0.3 | 1.5 | 1.0 | 0.3 | 6.0 | 4.3 | 1.0 | 3.8 |
| CGZ81_08040 | LBA1276 | 0.0 | 0.0 | 0.0 | 1.0 | 1.3 | 0.0 | 4.3 | 5.4 | 1.0 | 3.6 |
| CGZ81_03475 | recR | 0.0 | 0.0 | 0.0 | 2.0 | 0.0 | 0.3 | 7.7 | 1.0 | 2.1 | 3.6 |
| CGZ81_02375 | LBA0160 | 0.0 | 1.0 | 0.3 | 2.0 | 0.7 | 0.3 | 7.7 | 0.7 | 1.0 | 3.1 |
| CGZ81_03305 | radA | 2.0 | 0.0 | 0.0 | 1.0 | 1.3 | 0.7 | 0.6 | 5.4 | 3.2 | 3.1 |
| CGZ81_01510 | LBA1970 | 0.0 | 0.0 | 0.0 | 1.5 | 0.3 | 0.0 | 6.0 | 2.1 | 1.0 | 3.0 |
| CGZ81_09600 | pbpX | 0.0 | 0.0 | 0.3 | 0.5 | 1.3 | 0.3 | 2.7 | 5.4 | 1.0 | 3.0 |
| CGZ81_06545 | rluB | 0.0 | 1.3 | 0.0 | 2.0 | 0.3 | 0.0 | 7.7 | 0.4 | 1.0 | 3.0 |
| CGZ81_07830 | citX | 0.0 | 1.3 | 0.7 | 2.0 | 0.7 | 0.3 | 7.7 | 0.6 | 0.7 | 3.0 |
| CGZ81_01035 | LBA1872 | 0.0 | 0.0 | 0.7 | 1.0 | 0.7 | 1.0 | 4.3 | 3.2 | 1.3 | 3.0 |
| CGZ81_03645 | dinB | 0.0 | 1.0 | 0.0 | 1.0 | 0.0 | 1.0 | 4.3 | 0.2 | 4.3 | 3.0 |
| CGZ81_06000 | LBA0850 | 1.0 | 0.0 | 1.0 | 0.5 | 1.2 | 4.0 | 0.6 | 4.9 | 3.3 | 2.9 |
| CGZ81_04395 | gatB | 1.5 | 2.0 | 0.0 | 0.0 | 1.7 | 2.0 | 0.2 | 0.9 | 7.7 | 2.9 |
| CGZ81_04470 | LBA0547 | 0.5 | 0.0 | 0.0 | 0.5 | 1.0 | 0.7 | 1.0 | 4.3 | 3.2 | 2.9 |
| CGZ81_04785 | LBA0608 | 0.0 | 0.0 | 0.0 | 1.0 | 0.7 | 0.0 | 4.3 | 3.2 | 1.0 | 2.9 |
| CGZ81_06755 | LBA1007 | 0.0 | 0.3 | 0.0 | 0.0 | 0.3 | 1.7 | 1.0 | 1.0 | 6.6 | 2.9 |
| CGZ81_08300 | folD | 0.0 | 0.0 | 0.0 | 1.0 | 0.3 | 0.3 | 4.3 | 2.1 | 2.1 | 2.9 |
| CGZ81_02480 | pcp | 0.0 | 0.0 | 0.0 | 1.0 | 0.3 | 0.3 | 4.3 | 2.1 | 2.1 | 2.9 |
| CGZ81_07640 | pepT | 0.0 | 0.0 | 0.0 | 1.0 | 0.3 | 0.3 | 4.3 | 2.1 | 2.1 | 2.9 |
| CGZ81_07705 | LBA1204 | 0.0 | 0.7 | 0.3 | 1.5 | 0.7 | 0.7 | 6.0 | 1.0 | 1.5 | 2.8 |
| CGZ81_06015 | LBA0853 | 0.0 | 0.3 | 0.0 | 0.5 | 0.7 | 1.0 | 2.7 | 1.5 | 4.3 | 2.8 |
| CGZ81_04965 | ptsI | 0.0 | 2.0 | 0.0 | 1.0 | 1.7 | 0.7 | 4.3 | 0.9 | 3.2 | 2.8 |
| CGZ81_01020 | pgmB | 0.0 | 1.3 | 0.0 | 1.0 | 1.0 | 0.7 | 4.3 | 0.8 | 3.2 | 2.8 |
| CGZ81_05060 | LBA0661 | 0.0 | 1.0 | 0.0 | 0.5 | 0.0 | 1.3 | 2.7 | 0.2 | 5.4 | 2.8 |
| CGZ81_06130 | LBA0876 | 0.0 | 0.3 | 0.7 | 1.5 | 0.7 | 0.3 | 6.0 | 1.5 | 0.7 | 2.7 |
| CGZ81_02775 | LBA0246 | 0.0 | 0.0 | 0.0 | 1.5 | 0.0 | 0.0 | 6.0 | 1.0 | 1.0 | 2.7 |
| CGZ81_05200 | LBA0691 | 0.0 | 0.0 | 0.0 | 0.5 | 0.3 | 0.7 | 2.7 | 2.1 | 3.2 | 2.7 |
| CGZ81_07685 | recO | 0.0 | 0.0 | 0.0 | 0.5 | 1.0 | 0.0 | 2.7 | 4.3 | 1.0 | 2.7 |
| CGZ81_04345 | epsU | 0.0 | 0.0 | 0.3 | 1.0 | 0.3 | 0.7 | 4.3 | 2.1 | 1.5 | 2.7 |
| CGZ81_06020 | dapA | 0.5 | 0.0 | 0.0 | 0.0 | 0.3 | 1.3 | 0.4 | 2.1 | 5.4 | 2.6 |
| CGZ81_03705 | LBA0426 | 0.0 | 0.7 | 0.0 | 1.0 | 0.0 | 0.7 | 4.3 | 0.3 | 3.2 | 2.6 |
| CGZ81_07790 | LBA1220 | 1.0 | 0.7 | 0.0 | 1.5 | 1.7 | 1.0 | 1.4 | 2.0 | 4.3 | 2.6 |
| CGZ81_09095 | LBA1498 | 1.0 | 0.7 | 0.0 | 0.5 | 1.3 | 1.3 | 0.6 | 1.7 | 5.4 | 2.6 |
| CGZ81_02500 | LBA0190 | 1.0 | 0.0 | 0.3 | 2.0 | 1.3 | 0.0 | 1.8 | 5.4 | 0.5 | 2.6 |
| CGZ81_06120 | LBA0874 | 0.5 | 0.3 | 0.0 | 2.0 | 0.0 | 1.0 | 2.9 | 0.5 | 4.3 | 2.6 |
| CGZ81_09120 | miaA | 2.5 | 0.0 | 0.0 | 0.0 | 1.0 | 0.7 | 0.1 | 4.3 | 3.2 | 2.6 |
| CGZ81_01010 | msmK | 0.5 | 0.3 | 0.0 | 2.0 | 0.7 | 0.7 | 2.9 | 1.5 | 3.2 | 2.5 |
| CGZ81_09655 | leuS | 0.5 | 0.0 | 0.3 | 1.5 | 1.0 | 0.3 | 2.3 | 4.3 | 1.0 | 2.5 |
| CGZ81_09260 | LBA1531 | 0.0 | 1.0 | 0.0 | 1.5 | 0.3 | 0.0 | 6.0 | 0.5 | 1.0 | 2.5 |
| CGZ81_00385 | LBA1731 | 0.0 | 1.0 | 0.0 | 1.5 | 0.3 | 0.0 | 6.0 | 0.5 | 1.0 | 2.5 |
| CGZ81_03155 | rplQ | 0.0 | 0.3 | 0.3 | 1.5 | 0.0 | 0.3 | 6.0 | 0.5 | 1.0 | 2.5 |
| CGZ81_06610 | LBA0977 | 0.5 | 0.0 | 0.0 | 0.5 | 0.3 | 1.0 | 1.0 | 2.1 | 4.3 | 2.5 |
| CGZ81_09530 | LBA1591 | 0.5 | 0.0 | 0.3 | 0.5 | 1.0 | 1.0 | 1.0 | 4.3 | 2.1 | 2.5 |
| CGZ81_06080 | LBA0867 | 0.0 | 0.7 | 0.0 | 1.5 | 0.0 | 0.0 | 6.0 | 0.3 | 1.0 | 2.4 |
| CGZ81_00770 | LBA1816 | 1.0 | 1.3 | 0.0 | 2.5 | 1.0 | 1.0 | 2.2 | 0.8 | 4.3 | 2.4 |
| CGZ81_08070 | LBA1282 | 0.0 | 1.0 | 1.0 | 1.5 | 0.7 | 0.3 | 6.0 | 0.7 | 0.5 | 2.4 |
| CGZ81_04715 | LBA0594 | 0.5 | 0.7 | 0.0 | 1.0 | 2.0 | 0.7 | 1.6 | 2.4 | 3.2 | 2.4 |
| CGZ81_09350 | purH | 0.0 | 1.0 | 0.0 | 1.0 | 0.7 | 0.3 | 4.3 | 0.7 | 2.1 | 2.4 |
| CGZ81_04320 | LBA0520 | 1.5 | 0.0 | 0.0 | 3.0 | 1.0 | 0.0 | 1.8 | 4.3 | 1.0 | 2.4 |
| CGZ81_09340 | polA | 1.0 | 0.3 | 0.3 | 1.5 | 2.3 | 0.7 | 1.4 | 4.2 | 1.5 | 2.4 |
| CGZ81_03965 | LBA0447 | 1.0 | 0.0 | 0.0 | 0.5 | 0.7 | 0.7 | 0.6 | 3.2 | 3.2 | 2.4 |
| CGZ81_07815 | LBA1229 | 1.0 | 0.0 | 0.0 | 0.5 | 0.0 | 1.3 | 0.6 | 1.0 | 5.4 | 2.4 |
| CGZ81_09000 | LBA1478 | 1.0 | 0.0 | 0.0 | 0.5 | 1.3 | 0.0 | 0.6 | 5.4 | 1.0 | 2.4 |
| CGZ81_01475 | atkY | 0.5 | 0.3 | 0.0 | 1.5 | 0.7 | 0.7 | 2.3 | 1.5 | 3.2 | 2.3 |
| CGZ81_09280 | rplT | 0.5 | 0.0 | 0.0 | 1.0 | 0.3 | 0.7 | 1.6 | 2.1 | 3.2 | 2.3 |
| CGZ81_00605 | LBA1780 | 0.0 | 0.0 | 0.3 | 0.0 | 1.3 | 0.0 | 1.0 | 5.4 | 0.5 | 2.3 |
| CGZ81_01300 | dltA | 0.0 | 0.0 | 0.3 | 0.5 | 0.7 | 0.3 | 2.7 | 3.2 | 1.0 | 2.3 |
| CGZ81_03385 | LBA0361 | 1.5 | 0.0 | 0.3 | 0.5 | 1.3 | 0.3 | 0.4 | 5.4 | 1.0 | 2.3 |
| CGZ81_09800 | LBA1639 | 3.5 | 0.3 | 0.0 | 1.5 | 1.0 | 1.0 | 0.5 | 2.1 | 4.3 | 2.3 |
| CGZ81_02010 | rlmH | 0.0 | 0.3 | 0.0 | 0.5 | 1.0 | 0.3 | 2.7 | 2.1 | 2.1 | 2.3 |
| CGZ81_00600 | fruR | 0.5 | 0.0 | 0.0 | 0.0 | 1.3 | 0.0 | 0.4 | 5.4 | 1.0 | 2.3 |
| CGZ81_01230 | LBA1911 | 1.0 | 0.3 | 0.0 | 1.0 | 1.3 | 0.7 | 1.0 | 2.6 | 3.2 | 2.3 |
| CGZ81_00425 | LBA1739 | 0.5 | 1.0 | 1.0 | 2.5 | 3.0 | 0.7 | 3.5 | 2.5 | 0.7 | 2.3 |
| CGZ81_08225 | LBA1315 | 1.0 | 0.0 | 0.0 | 1.5 | 0.7 | 0.3 | 1.4 | 3.2 | 2.1 | 2.2 |
| CGZ81_04780 | LBA0607 | 0.0 | 0.7 | 0.3 | 1.0 | 0.0 | 1.0 | 4.3 | 0.3 | 2.1 | 2.2 |
| CGZ81_06845 | LBA1025 | 0.0 | 0.0 | 1.0 | 1.0 | 0.3 | 0.0 | 4.3 | 2.1 | 0.2 | 2.2 |
| CGZ81_04745 | LBA0600 | 2.5 | 0.3 | 0.0 | 3.5 | 1.0 | 0.7 | 1.4 | 2.1 | 3.2 | 2.2 |
| CGZ81_02700 | LBA0229 | 0.5 | 0.7 | 0.0 | 2.5 | 0.7 | 0.3 | 3.5 | 1.0 | 2.1 | 2.2 |
| CGZ81_06675 | xerD | 4.0 | 0.0 | 0.7 | 7.0 | 0.7 | 1.3 | 1.7 | 3.2 | 1.7 | 2.2 |
| CGZ81_07865 | LBA1239 | 2.5 | 6.3 | 0.0 | 3.5 | 5.7 | 1.0 | 1.4 | 0.9 | 4.3 | 2.2 |
| CGZ81_03355 | LBA0356 | 0.5 | 1.0 | 0.0 | 2.0 | 0.3 | 0.7 | 2.9 | 0.5 | 3.2 | 2.2 |
| CGZ81_06300 | LBA0909 | 2.0 | 0.0 | 0.0 | 0.0 | 1.0 | 0.3 | 0.1 | 4.3 | 2.1 | 2.2 |
| CGZ81_01490 | copA | 0.5 | 0.3 | 0.0 | 2.0 | 0.0 | 0.7 | 2.9 | 0.5 | 3.2 | 2.2 |
| CGZ81_07860 | LBA1238 | 0.0 | 1.0 | 1.7 | 1.5 | 0.0 | 0.3 | 6.0 | 0.2 | 0.3 | 2.2 |
| CGZ81_04915 | LBA0632 | 2.0 | 0.3 | 0.0 | 2.5 | 0.3 | 1.0 | 1.2 | 1.0 | 4.3 | 2.2 |
| CGZ81_07545 | mvaD | 0.0 | 0.7 | 0.0 | 0.5 | 0.3 | 0.7 | 2.7 | 0.7 | 3.2 | 2.2 |
| CGZ81_01825 | LBA0054 | 2.0 | 0.7 | 0.0 | 1.5 | 0.0 | 1.3 | 0.8 | 0.3 | 5.4 | 2.2 |
| CGZ81_05500 | LBA0754 | 0.5 | 0.3 | 0.0 | 2.0 | 0.7 | 0.3 | 2.9 | 1.5 | 2.1 | 2.2 |
| CGZ81_05070 | LBA0663 | 1.5 | 0.0 | 0.7 | 1.0 | 1.3 | 0.0 | 0.7 | 5.4 | 0.3 | 2.2 |
| CGZ81_02570 | pepE | 0.5 | 0.3 | 0.0 | 1.0 | 0.0 | 1.0 | 1.6 | 0.5 | 4.3 | 2.1 |
| CGZ81_07850 | LBA1236 | 1.0 | 2.0 | 0.0 | 0.5 | 3.0 | 1.0 | 0.6 | 1.4 | 4.3 | 2.1 |
| CGZ81_03140 | rpsM | 0.0 | 0.0 | 1.7 | 0.5 | 0.7 | 0.7 | 2.7 | 3.2 | 0.5 | 2.1 |
| CGZ81_01240 | LBA1913 | 3.5 | 0.0 | 0.3 | 2.0 | 0.3 | 2.0 | 0.6 | 2.1 | 3.6 | 2.1 |
| CGZ81_04790 | LBA0609 | 0.0 | 0.3 | 0.3 | 1.0 | 0.0 | 0.7 | 4.3 | 0.5 | 1.5 | 2.1 |
| CGZ81_04685 | rpiA | 0.0 | 0.3 | 0.3 | 1.0 | 0.7 | 0.0 | 4.3 | 1.5 | 0.5 | 2.1 |
| CGZ81_03270 | LBA0340 | 0.0 | 0.0 | 0.0 | 1.0 | 0.0 | 0.0 | 4.3 | 1.0 | 1.0 | 2.1 |
| CGZ81_03670 | LBA0419 | 0.0 | 0.0 | 0.0 | 1.0 | 0.0 | 0.0 | 4.3 | 1.0 | 1.0 | 2.1 |
| CGZ81_06790 | LBA1015 | 0.0 | 0.0 | 0.3 | 1.0 | 0.0 | 0.3 | 4.3 | 1.0 | 1.0 | 2.1 |
| CGZ81_07590 | LBA1178 | 1.0 | 0.7 | 0.0 | 1.0 | 0.7 | 1.0 | 1.0 | 1.0 | 4.3 | 2.1 |
| CGZ81_09140 | LBA1507 | 0.0 | 0.0 | 0.0 | 0.0 | 1.0 | 0.0 | 1.0 | 4.3 | 1.0 | 2.1 |
| CGZ81_00080 | LBA1671 | 0.0 | 0.3 | 0.0 | 1.0 | 0.3 | 0.0 | 4.3 | 1.0 | 1.0 | 2.1 |
| CGZ81_00255 | LBA1706 | 0.0 | 0.3 | 0.0 | 1.0 | 0.3 | 0.0 | 4.3 | 1.0 | 1.0 | 2.1 |
| CGZ81_09255 | nadD | 1.5 | 0.0 | 0.0 | 1.5 | 0.3 | 0.7 | 1.0 | 2.1 | 3.2 | 2.1 |
| CGZ81_05820 | sepF | 0.0 | 0.3 | 0.0 | 1.0 | 0.3 | 0.0 | 4.3 | 1.0 | 1.0 | 2.1 |
| CGZ81_03450 | tadA | 0.0 | 0.0 | 0.0 | 1.0 | 0.0 | 0.0 | 4.3 | 1.0 | 1.0 | 2.1 |
| CGZ81_05780 | LBA0805 | 0.5 | 1.0 | 0.7 | 1.5 | 2.3 | 1.7 | 2.3 | 2.0 | 2.0 | 2.1 |
| CGZ81_08935 | LBA1464 | 0.0 | 0.0 | 0.0 | 1.0 | 0.0 | 0.0 | 4.3 | 1.0 | 1.0 | 2.1 |
| CGZ81_00010 | LBA1657 | 0.0 | 1.0 | 0.0 | 0.5 | 1.7 | 0.3 | 2.7 | 1.5 | 2.1 | 2.1 |
| CGZ81_05925 | LBA0835 | 0.0 | 0.0 | 0.3 | 0.5 | 0.0 | 1.3 | 2.7 | 1.0 | 2.6 | 2.1 |
| CGZ81_02000 | LBA0082 | 2.0 | 0.3 | 0.0 | 3.0 | 1.3 | 0.3 | 1.4 | 2.6 | 2.1 | 2.0 |
| CGZ81_09180 | pepT | 0.5 | 0.0 | 1.0 | 1.0 | 0.7 | 1.3 | 1.6 | 3.2 | 1.3 | 2.0 |
| CGZ81_01455 | oppA | 0.5 | 0.3 | 0.7 | 2.0 | 0.7 | 1.3 | 2.9 | 1.5 | 1.7 | 2.0 |
| CGZ81_02555 | oppC | 0.0 | 1.0 | 1.0 | 1.0 | 0.7 | 1.0 | 4.3 | 0.7 | 1.0 | 2.0 |
| CGZ81_04340 | rodC | 0.0 | 1.0 | 0.0 | 1.0 | 0.7 | 0.0 | 4.3 | 0.7 | 1.0 | 2.0 |
| CGZ81_02465 | LBA0183 | 1.5 | 0.3 | 0.0 | 3.0 | 0.3 | 0.7 | 1.8 | 1.0 | 3.2 | 2.0 |
| CGZ81_03530 | tsaD | 0.5 | 0.0 | 4.3 | 1.0 | 1.0 | 0.0 | 1.6 | 4.3 | 0.1 | 2.0 |
| CGZ81_02250 | glnQ | 4.5 | 1.3 | 0.0 | 2.0 | 1.7 | 1.0 | 0.5 | 1.2 | 4.3 | 2.0 |
| CGZ81_06560 | cmk | 1.0 | 0.7 | 0.0 | 1.0 | 0.3 | 1.0 | 1.0 | 0.7 | 4.3 | 2.0 |
| CGZ81_06870 | LBA1031 | 0.0 | 0.7 | 0.0 | 1.0 | 0.3 | 0.0 | 4.3 | 0.7 | 1.0 | 2.0 |
| CGZ81_01380 | LBA1942 | 1.5 | 1.0 | 0.0 | 1.0 | 2.3 | 0.7 | 0.7 | 2.0 | 3.2 | 2.0 |
| CGZ81_07185 | LBA1094 | 0.5 | 1.0 | 0.0 | 0.0 | 1.3 | 1.0 | 0.4 | 1.3 | 4.3 | 2.0 |
| CGZ81_08540 | pyrB | 1.0 | 0.0 | 0.0 | 0.5 | 0.0 | 1.0 | 0.6 | 1.0 | 4.3 | 2.0 |
| CGZ81_09540 | pbpF | 3.5 | 0.7 | 0.0 | 2.0 | 0.7 | 1.0 | 0.6 | 1.0 | 4.3 | 2.0 |
| CGZ81_06525 | xerD | 0.5 | 0.7 | 0.0 | 2.5 | 0.0 | 0.3 | 3.5 | 0.3 | 2.1 | 2.0 |
| CGZ81_06320 | LBA0913 | 0.0 | 0.7 | 0.0 | 0.0 | 1.3 | 0.7 | 1.0 | 1.7 | 3.2 | 2.0 |
| CGZ81_05350 | licT | 0.0 | 0.7 | 0.0 | 0.0 | 1.3 | 0.7 | 1.0 | 1.7 | 3.2 | 2.0 |
| CGZ81_02335 | phnC | 1.5 | 2.0 | 0.0 | 1.0 | 1.7 | 1.0 | 0.7 | 0.9 | 4.3 | 2.0 |
| CGZ81_07220 | LBA1101 | 0.5 | 0.3 | 0.0 | 1.5 | 0.7 | 0.3 | 2.3 | 1.5 | 2.1 | 2.0 |
| CGZ81_04940 | prfC | 0.5 | 0.0 | 0.3 | 1.5 | 0.3 | 0.7 | 2.3 | 2.1 | 1.5 | 2.0 |
| CGZ81_01195 | LBA1903 | 3.0 | 0.7 | 0.3 | 1.0 | 2.0 | 1.7 | 0.4 | 2.4 | 3.1 | 2.0 |
| CGZ81_09150 | LBA1509 | 9.0 | 2.0 | 4.3 | 16.0 | 8.3 | 1.3 | 1.8 | 3.8 | 0.4 | 2.0 |
| CGZ81_07555 | fni | 1.5 | 1.0 | 0.3 | 1.0 | 2.3 | 1.7 | 0.7 | 2.0 | 3.1 | 2.0 |
| CGZ81_09105 | LBA1500 | 0.5 | 0.0 | 0.3 | 1.0 | 0.7 | 0.3 | 1.6 | 3.2 | 1.0 | 1.9 |
| CGZ81_04280 | pfoR | 0.5 | 0.0 | 0.3 | 1.0 | 0.7 | 0.3 | 1.6 | 3.2 | 1.0 | 1.9 |
| CGZ81_09440 | LBA1570 | 0.0 | 0.0 | 0.0 | 0.9 | 0.0 | 0.0 | 3.8 | 1.0 | 1.0 | 1.9 |
| CGZ81_02140 | LBA0113 | 0.0 | 1.0 | 0.3 | 1.0 | 0.3 | 0.3 | 4.3 | 0.5 | 1.0 | 1.9 |
| CGZ81_05480 | LBA0750 | 0.0 | 1.0 | 0.0 | 1.0 | 0.3 | 0.0 | 4.3 | 0.5 | 1.0 | 1.9 |
| CGZ81_01920 | arbZ | 0.0 | 0.3 | 0.0 | 0.0 | 0.0 | 1.0 | 1.0 | 0.5 | 4.3 | 1.9 |
| CGZ81_00215 | exoA | 0.0 | 0.0 | 0.3 | 1.0 | 0.0 | 0.0 | 4.3 | 1.0 | 0.5 | 1.9 |
| CGZ81_01135 | guaC | 0.5 | 0.3 | 0.0 | 0.5 | 0.0 | 1.0 | 1.0 | 0.5 | 4.3 | 1.9 |
| CGZ81_02625 | LBA0214 | 0.0 | 0.0 | 0.3 | 0.0 | 1.0 | 0.0 | 1.0 | 4.3 | 0.5 | 1.9 |
| CGZ81_01410 | nagB | 0.0 | 0.3 | 0.0 | 1.0 | 0.0 | 0.0 | 4.3 | 0.5 | 1.0 | 1.9 |
| CGZ81_09580 | nnrD | 0.0 | 0.3 | 0.0 | 1.0 | 0.0 | 0.0 | 4.3 | 0.5 | 1.0 | 1.9 |
| CGZ81_08090 | rplS | 0.0 | 0.3 | 0.0 | 1.0 | 0.0 | 0.0 | 4.3 | 0.5 | 1.0 | 1.9 |
| CGZ81_05995 | dapF | 0.5 | 0.3 | 0.0 | 1.0 | 1.0 | 0.3 | 1.6 | 2.1 | 2.1 | 1.9 |
| CGZ81_03615 | mutS | 2.0 | 0.7 | 0.0 | 1.5 | 0.3 | 1.0 | 0.8 | 0.7 | 4.3 | 1.9 |
| CGZ81_03610 | groL | 3.0 | 3.0 | 2.3 | 10.5 | 6.7 | 0.7 | 3.3 | 2.1 | 0.4 | 1.9 |
| CGZ81_03735 | LBA0432 | 0.0 | 1.3 | 0.0 | 1.0 | 0.3 | 0.0 | 4.3 | 0.4 | 1.0 | 1.9 |
| CGZ81_05420 | LBA0740 | 2.0 | 2.3 | 0.0 | 0.0 | 0.0 | 1.3 | 0.1 | 0.1 | 5.4 | 1.9 |
| CGZ81_07905 | dnaJ | 1.0 | 1.7 | 0.0 | 1.0 | 0.3 | 1.0 | 1.0 | 0.3 | 4.3 | 1.9 |
| CGZ81_09425 | LBA1567 | 6.0 | 1.7 | 3.0 | 10.5 | 5.7 | 2.7 | 1.7 | 3.0 | 0.9 | 1.9 |
| CGZ81_06945 | LBA1048 | 0.0 | 0.7 | 0.0 | 1.0 | 0.0 | 0.0 | 4.3 | 0.3 | 1.0 | 1.9 |
| CGZ81_04100 | LBA0474 | 1.0 | 0.0 | 0.7 | 2.5 | 0.3 | 1.0 | 2.2 | 2.1 | 1.3 | 1.9 |
| CGZ81_05580 | hemK | 1.0 | 0.0 | 0.0 | 1.5 | 0.7 | 0.0 | 1.4 | 3.2 | 1.0 | 1.9 |
| CGZ81_04380 | LBA0530 | 1.0 | 0.0 | 0.0 | 1.5 | 0.7 | 0.0 | 1.4 | 3.2 | 1.0 | 1.9 |
| CGZ81_08030 | LBA1274 | 1.0 | 0.7 | 0.0 | 1.5 | 0.7 | 0.7 | 1.4 | 1.0 | 3.2 | 1.9 |
| CGZ81_08335 | LBA1339 | 1.0 | 0.0 | 0.0 | 1.5 | 0.3 | 0.3 | 1.4 | 2.1 | 2.1 | 1.9 |
| CGZ81_05740 | mreB | 1.0 | 0.0 | 0.0 | 1.5 | 0.3 | 0.3 | 1.4 | 2.1 | 2.1 | 1.9 |
| CGZ81_04420 | LBA0539 | 1.5 | 0.7 | 0.3 | 1.5 | 2.7 | 0.7 | 1.0 | 3.1 | 1.5 | 1.9 |
| CGZ81_07650 | LBA1192 | 0.0 | 1.0 | 0.0 | 0.0 | 0.0 | 1.0 | 1.0 | 0.2 | 4.3 | 1.9 |
| CGZ81_07665 | rpoD | 1.0 | 0.0 | 0.7 | 0.0 | 1.0 | 0.7 | 0.2 | 4.3 | 1.0 | 1.9 |
| CGZ81_03080 | rplE | 5.0 | 0.3 | 7.3 | 6.5 | 2.3 | 0.3 | 1.3 | 4.2 | 0.1 | 1.8 |
| CGZ81_03225 | LBA0332 | 1.5 | 0.3 | 0.0 | 2.0 | 0.3 | 0.7 | 1.3 | 1.0 | 3.2 | 1.8 |
| CGZ81_09470 | LBA1578 | 3.5 | 9.0 | 4.7 | 10.0 | 16.0 | 4.7 | 2.7 | 1.8 | 1.0 | 1.8 |
| CGZ81_05385 | LBA0733 | 2.0 | 0.0 | 0.0 | 2.5 | 0.7 | 0.0 | 1.2 | 3.2 | 1.0 | 1.8 |
| CGZ81_09025 | rbsA | 1.5 | 0.0 | 1.7 | 2.5 | 0.7 | 1.0 | 1.6 | 3.2 | 0.7 | 1.8 |
| CGZ81_09110 | glnA | 1.0 | 0.0 | 0.3 | 0.5 | 0.7 | 0.7 | 0.6 | 3.2 | 1.5 | 1.8 |
| CGZ81_09810 | LBA1641 | 8.0 | 2.7 | 6.3 | 18.0 | 8.3 | 1.3 | 2.2 | 2.9 | 0.2 | 1.8 |
| CGZ81_07720 | LBA1207 | 0.5 | 0.0 | 0.0 | 1.5 | 0.0 | 0.3 | 2.3 | 1.0 | 2.1 | 1.8 |
| CGZ81_01025 | LBA1870 | 0.5 | 0.0 | 0.0 | 1.5 | 0.3 | 0.0 | 2.3 | 2.1 | 1.0 | 1.8 |
| CGZ81_08050 | LBA1278 | 3.5 | 0.0 | 1.0 | 4.0 | 0.7 | 1.0 | 1.1 | 3.2 | 1.0 | 1.8 |
| CGZ81_04605 | LBA0571 | 0.5 | 0.0 | 0.3 | 1.0 | 0.7 | 0.0 | 1.6 | 3.2 | 0.5 | 1.8 |
| CGZ81_01245 | LBA1914 | 3.0 | 1.0 | 0.3 | 2.0 | 1.7 | 1.7 | 0.7 | 1.5 | 3.1 | 1.8 |
| CGZ81_09610 | LBA1607 | 1.5 | 0.0 | 0.7 | 1.0 | 0.7 | 1.0 | 0.7 | 3.2 | 1.3 | 1.8 |
| CGZ81_05085 | rny | 0.5 | 1.3 | 0.3 | 1.5 | 3.0 | 0.3 | 2.3 | 2.0 | 1.0 | 1.8 |
| CGZ81_02865 | LBA0263 | 0.5 | 0.0 | 0.3 | 1.0 | 0.3 | 0.7 | 1.6 | 2.1 | 1.5 | 1.8 |
| CGZ81_07330 | parC | 1.5 | 0.0 | 0.3 | 0.5 | 1.0 | 0.0 | 0.4 | 4.3 | 0.5 | 1.8 |
| CGZ81_02255 | LBA0136 | 2.5 | 0.3 | 1.3 | 4.0 | 1.7 | 0.7 | 1.5 | 3.1 | 0.6 | 1.7 |
| CGZ81_09560 | LBA1598 | 2.0 | 0.0 | 0.3 | 2.0 | 0.7 | 0.3 | 1.0 | 3.2 | 1.0 | 1.7 |
| CGZ81_07940 | truB | 1.0 | 0.0 | 0.0 | 1.0 | 0.7 | 0.0 | 1.0 | 3.2 | 1.0 | 1.7 |
| CGZ81_08295 | xseA | 1.0 | 0.0 | 0.0 | 1.0 | 0.3 | 0.3 | 1.0 | 2.1 | 2.1 | 1.7 |
| CGZ81_07840 | LBA1234 | 0.5 | 1.0 | 0.0 | 2.0 | 0.0 | 0.3 | 2.9 | 0.2 | 2.1 | 1.7 |
| CGZ81_02940 | ftsH | 1.0 | 1.7 | 0.7 | 3.0 | 3.0 | 0.7 | 2.5 | 1.7 | 1.0 | 1.7 |
| CGZ81_05990 | engB | 0.5 | 3.0 | 0.0 | 0.0 | 1.3 | 1.0 | 0.4 | 0.5 | 4.3 | 1.7 |
| CGZ81_06060 | LBA0864 | 8.5 | 5.3 | 4.3 | 16.5 | 15.0 | 2.3 | 1.9 | 2.7 | 0.6 | 1.7 |
| CGZ81_04020 | manN | 1.5 | 0.0 | 0.3 | 0.5 | 0.7 | 0.7 | 0.4 | 3.2 | 1.5 | 1.7 |
| CGZ81_00405 | epsC | 0.0 | 0.3 | 0.0 | 0.5 | 0.7 | 0.0 | 2.7 | 1.5 | 1.0 | 1.7 |
| CGZ81_02245 | glnP | 1.0 | 1.3 | 0.3 | 3.0 | 0.7 | 1.0 | 2.5 | 0.6 | 2.1 | 1.7 |
| CGZ81_08515 | LBA1377 | 1.5 | 1.0 | 0.0 | 2.0 | 2.0 | 0.3 | 1.3 | 1.8 | 2.1 | 1.7 |
| CGZ81_05100 | LBA0670 | 2.0 | 1.0 | 0.3 | 1.5 | 1.3 | 1.7 | 0.8 | 1.3 | 3.1 | 1.7 |
| CGZ81_02475 | gpmA | 9.5 | 0.0 | 26.3 | 18.0 | 0.7 | 1.0 | 1.9 | 3.2 | 0.0 | 1.7 |
| CGZ81_03465 | tnpB | 0.0 | 0.0 | 0.0 | 0.6 | 0.0 | 0.0 | 3.1 | 1.0 | 1.0 | 1.7 |
| CGZ81_08390 | LBA1350 | 0.5 | 2.0 | 0.3 | 0.5 | 2.0 | 1.7 | 1.0 | 1.0 | 3.1 | 1.7 |
| CGZ81_01130 | purA | 0.0 | 1.0 | 2.0 | 1.0 | 0.3 | 0.3 | 4.3 | 0.5 | 0.3 | 1.7 |
| CGZ81_06435 | LBA0940 | 0.0 | 0.0 | 0.7 | 0.5 | 0.3 | 0.0 | 2.7 | 2.1 | 0.3 | 1.7 |
| CGZ81_02955 | lysS | 1.0 | 0.0 | 0.3 | 1.5 | 0.7 | 0.0 | 1.4 | 3.2 | 0.5 | 1.7 |
| CGZ81_08620 | sdhA | 1.0 | 0.7 | 0.7 | 1.0 | 1.0 | 2.3 | 1.0 | 1.3 | 2.7 | 1.7 |
| CGZ81_08140 | smc | 6.5 | 2.0 | 2.7 | 13.0 | 5.3 | 1.7 | 2.0 | 2.4 | 0.7 | 1.7 |
| CGZ81_06025 | dapB | 0.5 | 0.3 | 0.0 | 0.0 | 1.3 | 0.3 | 0.4 | 2.6 | 2.1 | 1.7 |
| CGZ81_09015 | rbsB | 0.5 | 0.3 | 0.3 | 0.0 | 1.7 | 0.7 | 0.4 | 3.1 | 1.5 | 1.7 |
| CGZ81_06180 | LBA0886 | 0.0 | 1.7 | 0.3 | 0.5 | 2.3 | 0.3 | 2.7 | 1.3 | 1.0 | 1.7 |
| CGZ81_02675 | prsA | 0.0 | 0.3 | 1.3 | 0.5 | 0.7 | 1.0 | 2.7 | 1.5 | 0.8 | 1.7 |
| CGZ81_03245 | LBA0335 | 1.0 | 0.0 | 0.3 | 0.0 | 0.7 | 0.7 | 0.2 | 3.2 | 1.5 | 1.7 |
| CGZ81_04070 | LBA0466 | 0.5 | 1.0 | 0.0 | 0.5 | 0.7 | 0.7 | 1.0 | 0.7 | 3.2 | 1.7 |
| CGZ81_01525 | LBA1973 | 1.0 | 0.3 | 0.0 | 1.5 | 1.3 | 0.0 | 1.4 | 2.6 | 1.0 | 1.7 |
| CGZ81_01295 | dltB | 0.0 | 1.3 | 0.0 | 0.5 | 0.0 | 0.3 | 2.7 | 0.2 | 2.1 | 1.7 |
| CGZ81_06465 | obg | 3.5 | 1.0 | 0.0 | 2.5 | 1.0 | 0.7 | 0.7 | 1.0 | 3.2 | 1.7 |
| CGZ81_05425 | ackB | 1.5 | 0.3 | 0.0 | 1.0 | 0.3 | 0.7 | 0.7 | 1.0 | 3.2 | 1.6 |
| CGZ81_08550 | pyrD | 1.0 | 0.0 | 0.7 | 1.5 | 0.7 | 0.0 | 1.4 | 3.2 | 0.3 | 1.6 |
| CGZ81_05975 | tuf | 14.5 | 2.3 | 15.7 | 11.5 | 9.7 | 5.0 | 0.8 | 3.8 | 0.3 | 1.6 |
| CGZ81_03025 | rplD | 1.5 | 0.3 | 0.7 | 3.0 | 1.0 | 0.7 | 1.8 | 2.1 | 1.0 | 1.6 |
| CGZ81_05590 | upp | 1.0 | 0.0 | 0.0 | 2.0 | 0.0 | 0.3 | 1.8 | 1.0 | 2.1 | 1.6 |
| CGZ81_05890 | LBA0827 | 0.0 | 0.7 | 0.0 | 0.0 | 0.3 | 0.7 | 1.0 | 0.7 | 3.2 | 1.6 |
| CGZ81_09460 | LBA1575 | 1.0 | 0.7 | 0.0 | 1.0 | 0.3 | 0.7 | 1.0 | 0.7 | 3.2 | 1.6 |
| CGZ81_01605 | LBA0011 | 0.5 | 0.3 | 1.0 | 2.0 | 0.3 | 1.0 | 2.9 | 1.0 | 1.0 | 1.6 |
| CGZ81_08780 | LBA1429 | 0.5 | 0.0 | 0.3 | 2.0 | 0.0 | 0.3 | 2.9 | 1.0 | 1.0 | 1.6 |
| CGZ81_05115 | secA | 3.5 | 1.0 | 0.7 | 2.0 | 0.3 | 3.3 | 0.6 | 0.5 | 3.8 | 1.6 |
| CGZ81_00700 | LBA1798 | 1.0 | 0.0 | 0.3 | 0.5 | 0.7 | 0.3 | 0.6 | 3.2 | 1.0 | 1.6 |
| CGZ81_04325 | nadC | 1.0 | 0.3 | 0.0 | 0.5 | 0.3 | 0.7 | 0.6 | 1.0 | 3.2 | 1.6 |
| CGZ81_08825 | melA | 3.0 | 2.7 | 0.7 | 3.0 | 3.0 | 2.3 | 1.0 | 1.1 | 2.7 | 1.6 |
| CGZ81_07580 | LBA1176 | 0.5 | 0.3 | 0.0 | 1.5 | 0.0 | 0.3 | 2.3 | 0.5 | 2.1 | 1.6 |
| CGZ81_02985 | rpoB | 1.5 | 2.0 | 0.7 | 5.0 | 1.7 | 0.7 | 2.9 | 0.9 | 1.0 | 1.6 |
| NA | slpA | 0.3 | 0.0 | 0.0 | 0.5 | 0.0 | 0.4 | 1.4 | 1.0 | 2.4 | 1.6 |
| CGZ81_05330 | LBA0718 | 0.2 | 0.3 | 0.3 | 1.5 | 0.0 | 0.3 | 3.3 | 0.5 | 1.0 | 1.6 |
| CGZ81_07325 | parE | 1.0 | 1.7 | 0.7 | 3.5 | 0.7 | 1.0 | 2.9 | 0.5 | 1.3 | 1.6 |
| CGZ81_05910 | bipA | 3.0 | 0.3 | 9.0 | 5.0 | 1.7 | 0.0 | 1.6 | 3.1 | 0.0 | 1.6 |
| CGZ81_04620 | LBA0574 | 0.5 | 0.0 | 0.0 | 1.0 | 0.3 | 0.0 | 1.6 | 2.1 | 1.0 | 1.6 |
| CGZ81_07745 | LBA1212 | 0.5 | 0.0 | 0.3 | 1.0 | 0.3 | 0.3 | 1.6 | 2.1 | 1.0 | 1.6 |
| CGZ81_00615 | LBA1783 | 0.5 | 0.0 | 0.0 | 1.0 | 0.0 | 0.3 | 1.6 | 1.0 | 2.1 | 1.6 |
| CGZ81_00905 | LBA1845 | 0.5 | 0.0 | 0.0 | 1.0 | 0.0 | 0.3 | 1.6 | 1.0 | 2.1 | 1.6 |
| CGZ81_05220 | LBA0695 | 26.5 | 45.7 | 24.0 | 48.0 | 79.7 | 28.7 | 1.8 | 1.7 | 1.2 | 1.6 |
| CGZ81_02905 | ldh1 | 5.0 | 0.3 | 0.0 | 5.5 | 0.7 | 0.3 | 1.1 | 1.5 | 2.1 | 1.6 |
| CGZ81_09570 | argS | 0.5 | 0.7 | 1.7 | 0.5 | 2.7 | 1.0 | 1.0 | 3.1 | 0.7 | 1.6 |
| CGZ81_04370 | pcrA | 2.5 | 1.3 | 1.0 | 4.0 | 2.0 | 2.0 | 1.5 | 1.4 | 1.8 | 1.6 |
| CGZ81_03490 | LBA0382 | 0.0 | 1.0 | 0.0 | 0.0 | 0.3 | 0.7 | 1.0 | 0.5 | 3.2 | 1.6 |
| CGZ81_01695 | LBA0029 | 1.0 | 1.0 | 0.0 | 0.0 | 1.3 | 0.7 | 0.2 | 1.3 | 3.2 | 1.6 |
| CGZ81_02540 | oppA | 3.0 | 2.3 | 1.3 | 4.5 | 1.3 | 4.0 | 1.5 | 0.6 | 2.6 | 1.6 |
| CGZ81_08275 | recN | 1.0 | 1.3 | 0.0 | 1.5 | 1.7 | 0.3 | 1.4 | 1.2 | 2.1 | 1.6 |
| CGZ81_06800 | LBA1017 | 1.0 | 0.3 | 0.0 | 1.0 | 0.0 | 0.7 | 1.0 | 0.5 | 3.2 | 1.6 |
| CGZ81_09650 | LBA1616 | 0.0 | 0.3 | 0.0 | 0.0 | 0.0 | 0.7 | 1.0 | 0.5 | 3.2 | 1.6 |
| CGZ81_08035 | pepO | 2.0 | 0.0 | 0.3 | 2.0 | 0.7 | 0.0 | 1.0 | 3.2 | 0.5 | 1.6 |
| CGZ81_06960 | LBA1050 | 1.5 | 0.3 | 0.0 | 3.5 | 0.0 | 0.3 | 2.1 | 0.5 | 2.1 | 1.6 |
| CGZ81_00135 | pacL | 2.5 | 2.0 | 0.3 | 4.0 | 1.0 | 1.3 | 1.5 | 0.6 | 2.6 | 1.6 |
| CGZ81_03655 | LBA0416 | 0.5 | 0.3 | 0.3 | 1.0 | 1.0 | 0.3 | 1.6 | 2.1 | 1.0 | 1.6 |
| CGZ81_09520 | LBA1589 | 1.5 | 0.0 | 0.0 | 0.5 | 0.7 | 0.0 | 0.4 | 3.2 | 1.0 | 1.6 |
| CGZ81_07870 | citG | 0.0 | 0.7 | 0.3 | 0.5 | 0.7 | 0.3 | 2.7 | 1.0 | 1.0 | 1.6 |
| CGZ81_08995 | LBA1477 | 0.0 | 0.7 | 0.3 | 0.5 | 0.7 | 0.3 | 2.7 | 1.0 | 1.0 | 1.6 |
| CGZ81_00865 | LBA1837 | 0.0 | 0.7 | 0.0 | 0.5 | 0.7 | 0.0 | 2.7 | 1.0 | 1.0 | 1.6 |
| CGZ81_05720 | valS | 2.0 | 1.7 | 1.3 | 4.5 | 2.0 | 2.0 | 2.1 | 1.2 | 1.4 | 1.6 |
| CGZ81_00910 | LBA1846 | 2.0 | 0.7 | 0.0 | 1.5 | 0.3 | 0.7 | 0.8 | 0.7 | 3.2 | 1.6 |
| CGZ81_03180 | rplM | 3.5 | 1.3 | 0.0 | 4.0 | 2.0 | 0.3 | 1.1 | 1.4 | 2.1 | 1.6 |
| CGZ81_06365 | LBA0923 | 1.0 | 0.7 | 0.3 | 2.0 | 1.0 | 0.7 | 1.8 | 1.3 | 1.5 | 1.5 |
| CGZ81_06720 | recQ | 2.0 | 2.0 | 1.0 | 4.0 | 2.0 | 2.0 | 1.9 | 1.0 | 1.8 | 1.5 |
| CGZ81_06930 | LBA1045 | 1.5 | 0.3 | 0.0 | 1.5 | 0.7 | 0.3 | 1.0 | 1.5 | 2.1 | 1.5 |
| CGZ81_00235 | rpiR | 2.5 | 1.3 | 0.7 | 3.0 | 0.3 | 2.7 | 1.2 | 0.4 | 3.1 | 1.5 |
| CGZ81_05170 | LBA0685 | 1.5 | 1.0 | 0.3 | 3.0 | 0.7 | 1.0 | 1.8 | 0.7 | 2.1 | 1.5 |
| CGZ81_06295 | LBA0908 | 1.0 | 1.3 | 0.7 | 2.5 | 2.7 | 0.3 | 2.2 | 1.8 | 0.7 | 1.5 |
| CGZ81_09115 | LBA1502 | 1.5 | 0.3 | 0.0 | 0.5 | 1.0 | 0.3 | 0.4 | 2.1 | 2.1 | 1.5 |
| CGZ81_08575 | LBA1389 | 2.5 | 2.3 | 0.3 | 2.5 | 1.0 | 1.7 | 1.0 | 0.5 | 3.1 | 1.5 |
| CGZ81_02305 | LBA0145 | 0.5 | 0.0 | 0.0 | 0.0 | 0.7 | 0.0 | 0.4 | 3.2 | 1.0 | 1.5 |
| CGZ81_06615 | LBA0978 | 0.5 | 0.7 | 0.0 | 0.0 | 0.7 | 0.7 | 0.4 | 1.0 | 3.2 | 1.5 |
| CGZ81_07050 | LBA1068 | 0.5 | 0.0 | 0.0 | 0.0 | 0.7 | 0.0 | 0.4 | 3.2 | 1.0 | 1.5 |
| CGZ81_02615 | LBA0212 | 2.0 | 1.3 | 0.0 | 1.5 | 0.7 | 0.7 | 0.8 | 0.6 | 3.2 | 1.5 |
| CGZ81_06620 | rnhB | 0.5 | 1.0 | 0.0 | 1.5 | 0.0 | 0.3 | 2.3 | 0.2 | 2.1 | 1.5 |
| CGZ81_09100 | LBA1499 | 0.5 | 2.0 | 0.0 | 1.0 | 1.7 | 0.3 | 1.6 | 0.9 | 2.1 | 1.5 |
| CGZ81_01800 | LBA0049 | 1.0 | 0.3 | 0.3 | 1.0 | 0.0 | 1.7 | 1.0 | 0.5 | 3.1 | 1.5 |
| CGZ81_02265 | LBA0138 | 1.0 | 0.3 | 0.3 | 1.0 | 1.0 | 0.7 | 1.0 | 2.1 | 1.5 | 1.5 |
| CGZ81_07715 | LBA1206 | 0.5 | 1.3 | 0.3 | 1.0 | 2.0 | 0.7 | 1.6 | 1.4 | 1.5 | 1.5 |
| CGZ81_07440 | LBA1149 | 2.0 | 0.3 | 0.7 | 1.5 | 1.7 | 0.3 | 0.8 | 3.1 | 0.7 | 1.5 |
| CGZ81_02950 | LBA0280 | 1.0 | 0.3 | 0.7 | 0.5 | 1.3 | 1.0 | 0.6 | 2.6 | 1.3 | 1.5 |
| CGZ81_02280 | LBA0141 | 0.5 | 1.3 | 0.0 | 1.0 | 1.0 | 0.3 | 1.6 | 0.8 | 2.1 | 1.5 |
| CGZ81_04255 | bfrA | 0.5 | 0.7 | 0.3 | 2.0 | 0.3 | 0.3 | 2.9 | 0.7 | 1.0 | 1.5 |
| CGZ81_02360 | LBA0157 | 0.5 | 3.3 | 0.0 | 2.0 | 2.0 | 0.0 | 2.9 | 0.6 | 1.0 | 1.5 |
| CGZ81_02295 | LBA0143 | 1.0 | 0.3 | 0.0 | 1.5 | 0.3 | 0.3 | 1.4 | 1.0 | 2.1 | 1.5 |
| CGZ81_05855 | LBA0820 | 1.0 | 0.0 | 0.0 | 1.5 | 0.0 | 0.3 | 1.4 | 1.0 | 2.1 | 1.5 |
| CGZ81_07780 | LBA1218 | 2.0 | 0.3 | 0.3 | 3.0 | 1.3 | 0.0 | 1.4 | 2.6 | 0.5 | 1.5 |
| CGZ81_01625 | LBA0015 | 1.0 | 0.0 | 0.0 | 0.0 | 0.0 | 0.7 | 0.2 | 1.0 | 3.2 | 1.5 |
| CGZ81_05235 | LBA0697 | 1.0 | 0.0 | 0.0 | 0.0 | 0.0 | 0.7 | 0.2 | 1.0 | 3.2 | 1.5 |
| CGZ81_09835 | LBA1646 | 1.0 | 0.0 | 0.0 | 0.0 | 0.7 | 0.0 | 0.2 | 3.2 | 1.0 | 1.5 |
| CGZ81_00225 | LBA1701 | 1.0 | 0.0 | 0.0 | 0.0 | 0.7 | 0.0 | 0.2 | 3.2 | 1.0 | 1.5 |
| CGZ81_06380 | LBA0927 | 1.0 | 0.0 | 0.0 | 0.0 | 0.3 | 0.3 | 0.2 | 2.1 | 2.1 | 1.5 |
| CGZ81_09845 | LBA1648 | 2.0 | 0.7 | 0.3 | 2.5 | 0.3 | 1.3 | 1.2 | 0.7 | 2.6 | 1.5 |
| CGZ81_09630 | LBA1612 | 2.0 | 1.7 | 1.0 | 2.0 | 3.0 | 2.0 | 1.0 | 1.7 | 1.8 | 1.5 |
| CGZ81_01735 | LBA0036 | 1.0 | 2.0 | 0.0 | 2.0 | 1.0 | 0.3 | 1.8 | 0.6 | 2.1 | 1.5 |
| CGZ81_02705 | LBA0230 | 1.0 | 0.3 | 0.0 | 1.5 | 1.0 | 0.0 | 1.4 | 2.1 | 1.0 | 1.5 |
| CGZ81_04895 | hmcS | 2.0 | 0.3 | 0.7 | 0.5 | 1.0 | 1.7 | 0.3 | 2.1 | 2.0 | 1.5 |
| CGZ81_03405 | pstB2 | 0.5 | 0.3 | 0.7 | 1.5 | 0.7 | 0.3 | 2.3 | 1.5 | 0.7 | 1.5 |
| CGZ81_02980 | clpC | 2.5 | 1.7 | 0.7 | 4.5 | 1.0 | 1.7 | 1.7 | 0.7 | 2.0 | 1.5 |
| CGZ81_07675 | glyS | 2.5 | 2.0 | 0.0 | 1.0 | 1.3 | 0.7 | 0.5 | 0.7 | 3.2 | 1.5 |
| CGZ81_01760 | rtpR | 1.0 | 2.0 | 1.0 | 3.0 | 1.7 | 1.0 | 2.5 | 0.9 | 1.0 | 1.5 |
| CGZ81_08445 | lacA | 1.5 | 0.0 | 0.0 | 0.0 | 0.0 | 0.7 | 0.2 | 1.0 | 3.2 | 1.5 |
| CGZ81_05355 | LBA0725 | 1.5 | 0.7 | 0.7 | 1.5 | 1.0 | 1.7 | 1.0 | 1.3 | 2.0 | 1.5 |
| CGZ81_06860 | LBA1029 | 1.5 | 6.7 | 2.7 | 3.0 | 14.3 | 1.0 | 1.8 | 2.1 | 0.4 | 1.5 |
| CGZ81_06155 | LBA0881 | 2.0 | 0.3 | 0.0 | 1.5 | 1.3 | 0.0 | 0.8 | 2.6 | 1.0 | 1.5 |
| CGZ81_09165 | LBA1512 | 2.0 | 1.0 | 0.7 | 4.5 | 1.3 | 0.7 | 2.1 | 1.3 | 1.0 | 1.4 |
| CGZ81_05585 | LBA0769 | 1.0 | 0.3 | 0.0 | 0.0 | 1.7 | 0.0 | 0.2 | 3.1 | 1.0 | 1.4 |
| CGZ81_01985 | LBA0079 | 1.5 | 1.3 | 0.0 | 1.0 | 0.3 | 0.7 | 0.7 | 0.4 | 3.2 | 1.4 |
| CGZ81_05525 | LBA0757 | 1.5 | 1.7 | 0.0 | 0.5 | 1.0 | 0.7 | 0.4 | 0.7 | 3.2 | 1.4 |
| CGZ81_02915 | trcF | 0.5 | 1.3 | 0.7 | 1.5 | 0.3 | 1.3 | 2.3 | 0.4 | 1.7 | 1.4 |
| CGZ81_06770 | LBA1011 | 0.5 | 2.7 | 0.3 | 1.5 | 1.3 | 0.7 | 2.3 | 0.6 | 1.5 | 1.4 |
| CGZ81_03620 | mutL | 3.5 | 1.7 | 0.0 | 2.0 | 0.7 | 0.7 | 0.6 | 0.5 | 3.2 | 1.4 |
| CGZ81_01860 | LBA0060 | 1.5 | 0.7 | 0.7 | 2.0 | 1.7 | 0.7 | 1.3 | 2.0 | 1.0 | 1.4 |
| CGZ81_05620 | atpG | 1.0 | 1.3 | 0.0 | 1.5 | 1.0 | 0.3 | 1.4 | 0.8 | 2.1 | 1.4 |
| CGZ81_02190 | epsV | 2.0 | 1.0 | 0.0 | 3.0 | 0.7 | 0.3 | 1.4 | 0.7 | 2.1 | 1.4 |
| CGZ81_09090 | LBA1497 | 3.0 | 0.3 | 0.0 | 2.0 | 1.3 | 0.0 | 0.7 | 2.6 | 1.0 | 1.4 |
| CGZ81_01280 | LBA1922 | 2.0 | 0.7 | 0.3 | 2.5 | 0.7 | 1.0 | 1.2 | 1.0 | 2.1 | 1.4 |
| CGZ81_00170 | LBA1690 | 43.0 | 3.0 | 11.3 | 37.0 | 10.0 | 3.0 | 0.9 | 3.1 | 0.3 | 1.4 |
| CGZ81_08400 | LBA1352 | 2.0 | 1.3 | 0.3 | 3.5 | 2.3 | 0.3 | 1.7 | 1.6 | 1.0 | 1.4 |
| CGZ81_06690 | pepD | 0.0 | 1.3 | 0.3 | 0.5 | 0.7 | 0.3 | 2.7 | 0.6 | 1.0 | 1.4 |
| CGZ81_04740 | LBA0599 | 1.0 | 0.7 | 0.3 | 1.5 | 1.0 | 0.7 | 1.4 | 1.3 | 1.5 | 1.4 |
| CGZ81_00920 | dtpT | 1.0 | 0.3 | 0.0 | 0.5 | 0.7 | 0.3 | 0.6 | 1.5 | 2.1 | 1.4 |
| CGZ81_08715 | LBA1416 | 0.5 | 0.3 | 0.3 | 1.5 | 0.7 | 0.0 | 2.3 | 1.5 | 0.5 | 1.4 |
| CGZ81_07755 | LBA1215 | 0.0 | 1.7 | 0.7 | 0.5 | 1.5 | 0.3 | 2.7 | 0.9 | 0.7 | 1.4 |
| CGZ81_08470 | celB | 1.0 | 1.0 | 0.0 | 1.5 | 0.7 | 0.3 | 1.4 | 0.7 | 2.1 | 1.4 |
| CGZ81_07895 | srtA | 2.0 | 0.0 | 1.0 | 0.5 | 0.3 | 2.0 | 0.3 | 2.1 | 1.8 | 1.4 |
| CGZ81_00870 | LBA1838 | 0.0 | 0.7 | 0.3 | 0.0 | 1.3 | 0.7 | 1.0 | 1.7 | 1.5 | 1.4 |
| CGZ81_04950 | clpE | 3.5 | 1.3 | 0.0 | 2.0 | 0.3 | 0.7 | 0.6 | 0.4 | 3.2 | 1.4 |
| CGZ81_04195 | LBA0492 | 0.5 | 0.0 | 0.3 | 1.0 | 0.3 | 0.0 | 1.6 | 2.1 | 0.5 | 1.4 |
| CGZ81_07800 | LBA1225 | 0.5 | 0.0 | 0.3 | 1.0 | 0.3 | 0.0 | 1.6 | 2.1 | 0.5 | 1.4 |
| CGZ81_09465 | LBA1577 | 1.0 | 0.3 | 0.0 | 0.5 | 1.3 | 0.0 | 0.6 | 2.6 | 1.0 | 1.4 |
| CGZ81_07175 | ppc | 1.5 | 0.3 | 0.7 | 3.0 | 0.3 | 1.0 | 1.8 | 1.0 | 1.3 | 1.4 |
| CGZ81_05985 | clpX | 1.5 | 0.7 | 0.0 | 1.0 | 1.0 | 0.3 | 0.7 | 1.3 | 2.1 | 1.4 |
| CGZ81_01335 | LBA1932 | 1.5 | 1.3 | 0.0 | 0.5 | 2.3 | 0.3 | 0.4 | 1.6 | 2.1 | 1.4 |
| CGZ81_02895 | alr | 0.5 | 1.0 | 0.3 | 1.0 | 1.0 | 0.7 | 1.6 | 1.0 | 1.5 | 1.4 |
| CGZ81_07750 | thrC | 2.0 | 0.3 | 0.7 | 1.0 | 1.3 | 0.7 | 0.6 | 2.6 | 1.0 | 1.4 |
| CGZ81_08635 | oppA | 4.0 | 2.3 | 0.7 | 2.5 | 2.8 | 2.0 | 0.7 | 1.2 | 2.3 | 1.4 |
| CGZ81_00565 | LBA1772 | 1.5 | 0.3 | 0.0 | 0.5 | 0.0 | 0.7 | 0.4 | 0.5 | 3.2 | 1.4 |
| CGZ81_01935 | arbx | 1.0 | 0.3 | 0.0 | 1.0 | 0.3 | 0.3 | 1.0 | 1.0 | 2.1 | 1.4 |
| CGZ81_03685 | LBA0422 | 1.0 | 0.3 | 0.0 | 1.0 | 0.3 | 0.3 | 1.0 | 1.0 | 2.1 | 1.4 |
| CGZ81_03725 | LBA0430 | 1.0 | 0.0 | 0.0 | 1.0 | 0.3 | 0.0 | 1.0 | 2.1 | 1.0 | 1.4 |
| CGZ81_05225 | LBA0696 | 0.0 | 0.0 | 0.7 | 0.0 | 0.3 | 0.7 | 1.0 | 2.1 | 1.0 | 1.4 |
| CGZ81_07825 | LBA1231 | 0.0 | 0.7 | 0.0 | 0.0 | 0.7 | 0.3 | 1.0 | 1.0 | 2.1 | 1.4 |
| CGZ81_07340 | ppaC | 0.0 | 0.7 | 0.0 | 0.0 | 0.7 | 0.3 | 1.0 | 1.0 | 2.1 | 1.4 |
| CGZ81_05030 | pthA | 1.0 | 0.0 | 0.0 | 1.0 | 0.0 | 0.3 | 1.0 | 1.0 | 2.1 | 1.4 |
| CGZ81_01165 | frnE | 4.5 | 1.0 | 2.7 | 8.5 | 0.3 | 5.0 | 1.8 | 0.5 | 1.8 | 1.4 |
| CGZ81_02505 | LBA0191 | 69.0 | 24.0 | 45.7 | 79.5 | 58.7 | 24.0 | 1.2 | 2.4 | 0.5 | 1.4 |
| CGZ81_04760 | LBA0603 | 0.0 | 2.0 | 0.0 | 0.5 | 0.7 | 0.0 | 2.7 | 0.4 | 1.0 | 1.4 |
| CGZ81_08565 | pyrE | 2.0 | 0.3 | 0.0 | 4.5 | 0.3 | 0.0 | 2.1 | 1.0 | 1.0 | 1.4 |
| CGZ81_03095 | rplF | 1.5 | 0.0 | 0.3 | 2.5 | 0.0 | 0.7 | 1.6 | 1.0 | 1.5 | 1.4 |
| CGZ81_08340 | LBA1340 | 1.5 | 0.3 | 0.0 | 0.5 | 0.7 | 0.3 | 0.4 | 1.5 | 2.1 | 1.4 |
| CGZ81_05305 | potD | 1.0 | 1.0 | 0.0 | 0.5 | 0.0 | 0.7 | 0.6 | 0.2 | 3.2 | 1.4 |
| CGZ81_06505 | LBA0955 | 1.5 | 1.0 | 1.0 | 2.5 | 1.7 | 1.0 | 1.6 | 1.5 | 1.0 | 1.4 |
| CGZ81_06705 | LBA0997 | 3.0 | 3.0 | 0.0 | 1.5 | 0.7 | 0.7 | 0.5 | 0.3 | 3.2 | 1.4 |
| CGZ81_08450 | LBA1365 | 0.5 | 1.7 | 0.0 | 1.0 | 0.3 | 0.3 | 1.6 | 0.3 | 2.1 | 1.4 |
| CGZ81_04465 | LBA0546 | 0.0 | 1.3 | 0.3 | 0.5 | 0.3 | 0.3 | 2.7 | 0.4 | 1.0 | 1.4 |
| NA | gltX | 1.5 | 0.3 | 0.3 | 1.5 | 0.7 | 0.7 | 1.0 | 1.5 | 1.5 | 1.4 |
| CGZ81_03955 | LBA0445 | 1.5 | 0.3 | 0.3 | 1.5 | 0.3 | 1.0 | 1.0 | 1.0 | 2.1 | 1.4 |
| CGZ81_06445 | LBA0942 | 1.5 | 0.3 | 0.3 | 1.5 | 0.3 | 1.0 | 1.0 | 1.0 | 2.1 | 1.4 |
| CGZ81_02840 | LBA0258 | 0.5 | 2.0 | 0.3 | 1.5 | 0.3 | 0.7 | 2.3 | 0.3 | 1.5 | 1.4 |
| CGZ81_00800 | LBA1822 | 2.0 | 0.0 | 0.3 | 0.5 | 0.7 | 0.0 | 0.3 | 3.2 | 0.5 | 1.3 |
| CGZ81_05185 | uvrB | 1.0 | 0.7 | 0.3 | 1.0 | 1.7 | 0.3 | 1.0 | 2.0 | 1.0 | 1.3 |
| CGZ81_08525 | carB | 3.5 | 2.0 | 0.3 | 0.5 | 1.3 | 1.7 | 0.2 | 0.7 | 3.1 | 1.3 |
| CGZ81_06115 | LBA0873 | 1.5 | 0.3 | 0.3 | 0.5 | 1.7 | 0.0 | 0.4 | 3.1 | 0.5 | 1.3 |
| CGZ81_05690 | ezrA | 2.0 | 0.0 | 0.7 | 1.0 | 0.3 | 1.0 | 0.6 | 2.1 | 1.3 | 1.3 |
| CGZ81_04055 | ackA | 2.5 | 0.0 | 1.7 | 1.5 | 0.7 | 0.0 | 0.6 | 3.2 | 0.2 | 1.3 |
| CGZ81_09325 | nrdR | 0.5 | 0.3 | 0.0 | 0.0 | 0.7 | 0.3 | 0.4 | 1.5 | 2.1 | 1.3 |
| CGZ81_05025 | pacL | 2.0 | 1.3 | 1.0 | 2.5 | 1.3 | 2.0 | 1.2 | 1.0 | 1.8 | 1.3 |
| CGZ81_06415 | hisS | 1.0 | 1.0 | 0.0 | 1.5 | 0.3 | 0.3 | 1.4 | 0.5 | 2.1 | 1.3 |
| CGZ81_05680 | LBA0785 | 0.0 | 0.7 | 0.0 | 0.5 | 0.0 | 0.0 | 2.7 | 0.3 | 1.0 | 1.3 |
| CGZ81_07420 | LBA1145 | 1.0 | 0.3 | 0.0 | 1.5 | 0.0 | 0.3 | 1.4 | 0.5 | 2.1 | 1.3 |
| CGZ81_03680 | mutS2 | 4.5 | 0.3 | 0.0 | 1.5 | 1.3 | 0.0 | 0.4 | 2.6 | 1.0 | 1.3 |
| CGZ81_04770 | LBA0605 | 0.0 | 1.3 | 0.3 | 0.5 | 1.0 | 0.0 | 2.7 | 0.8 | 0.5 | 1.3 |
| CGZ81_00820 | parB | 1.0 | 0.3 | 0.0 | 0.0 | 0.0 | 0.7 | 0.2 | 0.5 | 3.2 | 1.3 |
| CGZ81_04775 | LBA0606 | 1.0 | 0.3 | 0.3 | 1.5 | 0.7 | 0.3 | 1.4 | 1.5 | 1.0 | 1.3 |
| CGZ81_09155 | LBA1510 | 1.0 | 1.0 | 0.0 | 1.5 | 1.7 | 0.0 | 1.4 | 1.5 | 1.0 | 1.3 |
| CGZ81_06355 | LBA0921 | 2.0 | 2.0 | 0.3 | 4.0 | 2.0 | 0.3 | 1.9 | 1.0 | 1.0 | 1.3 |
| CGZ81_01495 | gdmH | 1.5 | 0.0 | 0.3 | 0.0 | 0.7 | 0.0 | 0.2 | 3.2 | 0.5 | 1.3 |
| CGZ81_05365 | LBA0728 | 3.5 | 2.0 | 0.7 | 2.5 | 3.0 | 1.3 | 0.7 | 1.4 | 1.7 | 1.3 |
| CGZ81_03105 | rpsE | 1.0 | 0.7 | 1.0 | 2.0 | 1.0 | 0.7 | 1.8 | 1.3 | 0.7 | 1.3 |
| CGZ81_09315 | dnaI | 0.5 | 1.0 | 0.0 | 0.5 | 0.7 | 0.3 | 1.0 | 0.7 | 2.1 | 1.3 |
| CGZ81_09585 | vanY | 2.0 | 1.7 | 0.3 | 1.5 | 1.7 | 1.0 | 0.8 | 1.0 | 2.1 | 1.3 |
| CGZ81_00675 | LBA1793 | 3.5 | 1.3 | 0.7 | 2.5 | 2.0 | 1.3 | 0.7 | 1.4 | 1.7 | 1.3 |
| CGZ81_02340 | LBA0153 | 1.5 | 0.0 | 0.0 | 1.0 | 0.3 | 0.0 | 0.7 | 2.1 | 1.0 | 1.3 |
| CGZ81_05180 | LBA0687 | 3.0 | 0.3 | 0.3 | 0.5 | 0.7 | 1.0 | 0.2 | 1.5 | 2.1 | 1.3 |
| CGZ81_06420 | aspS | 1.0 | 1.3 | 0.0 | 1.0 | 2.7 | 0.0 | 1.0 | 1.8 | 1.0 | 1.3 |
| CGZ81_02325 | LBA0150 | 0.0 | 1.3 | 0.0 | 0.0 | 2.7 | 0.0 | 1.0 | 1.8 | 1.0 | 1.3 |
| CGZ81_06580 | LBA0971 | 1.5 | 0.0 | 0.3 | 0.0 | 0.3 | 0.7 | 0.2 | 2.1 | 1.5 | 1.3 |
| CGZ81_08425 | LBA1357 | 2.0 | 0.0 | 0.7 | 0.5 | 0.3 | 1.0 | 0.3 | 2.1 | 1.3 | 1.3 |
| CGZ81_07910 | dnaK | 3.5 | 0.7 | 0.7 | 0.0 | 2.0 | 1.0 | 0.1 | 2.4 | 1.3 | 1.3 |
| CGZ81_02385 | LBA0162 | 0.0 | 0.7 | 0.3 | 0.5 | 0.3 | 0.0 | 2.7 | 0.7 | 0.5 | 1.3 |
| CGZ81_02135 | LBA0112 | 4.0 | 0.3 | 1.3 | 1.5 | 1.3 | 1.0 | 0.4 | 2.6 | 0.8 | 1.3 |
| CGZ81_06510 | pfkA | 3.0 | 1.0 | 0.3 | 3.0 | 1.3 | 0.7 | 1.0 | 1.3 | 1.5 | 1.3 |
| CGZ81_06775 | treB | 1.5 | 0.0 | 1.3 | 2.0 | 0.3 | 0.3 | 1.3 | 2.1 | 0.4 | 1.3 |
| CGZ81_09515 | prtM | 3.5 | 0.7 | 2.0 | 1.0 | 2.3 | 1.3 | 0.3 | 2.7 | 0.7 | 1.3 |
| CGZ81_03730 | ccpA | 2.5 | 1.7 | 0.0 | 2.5 | 1.0 | 0.3 | 1.0 | 0.7 | 2.1 | 1.3 |
| CGZ81_02710 | LBA0231 | 1.0 | 0.0 | 0.0 | 2.0 | 0.0 | 0.0 | 1.8 | 1.0 | 1.0 | 1.3 |
| CGZ81_07180 | LBA1093 | 1.0 | 1.0 | 0.7 | 1.0 | 2.0 | 0.7 | 1.0 | 1.8 | 1.0 | 1.3 |
| CGZ81_01530 | poxB | 1.0 | 0.3 | 1.0 | 3.0 | 0.3 | 0.0 | 2.5 | 1.0 | 0.2 | 1.3 |
| CGZ81_04110 | LBA0475 | 1.0 | 1.0 | 1.0 | 2.0 | 1.3 | 0.7 | 1.8 | 1.3 | 0.7 | 1.3 |
| CGZ81_02515 | LBA0193 | 0.0 | 0.0 | 0.7 | 0.0 | 0.3 | 0.3 | 1.0 | 2.1 | 0.7 | 1.3 |
| CGZ81_04595 | LBA0569 | 0.0 | 0.0 | 0.7 | 0.0 | 0.3 | 0.3 | 1.0 | 2.1 | 0.7 | 1.3 |
| CGZ81_09310 | thrS | 0.0 | 0.7 | 0.0 | 0.0 | 0.3 | 0.3 | 1.0 | 0.7 | 2.1 | 1.3 |
| CGZ81_00990 | LBA1863 | 1.0 | 0.7 | 0.0 | 0.0 | 0.0 | 0.7 | 0.2 | 0.3 | 3.2 | 1.3 |
| CGZ81_01505 | flpA | 4.0 | 0.0 | 0.0 | 2.5 | 0.0 | 0.3 | 0.7 | 1.0 | 2.1 | 1.3 |
| CGZ81_09240 | LBA1527 | 2.5 | 0.3 | 0.0 | 1.5 | 0.3 | 0.3 | 0.6 | 1.0 | 2.1 | 1.3 |
| CGZ81_02765 | guaA | 1.0 | 1.0 | 1.3 | 2.5 | 1.0 | 0.7 | 2.2 | 1.0 | 0.6 | 1.2 |
| CGZ81_07760 | LBA1216 | 2.0 | 0.3 | 0.3 | 2.5 | 0.0 | 1.0 | 1.2 | 0.5 | 2.1 | 1.2 |
| CGZ81_09435 | LBA1569 | 0.0 | 0.3 | 0.0 | 0.4 | 0.0 | 0.0 | 2.3 | 0.5 | 1.0 | 1.2 |
| CGZ81_09855 | LBA1650 | 0.5 | 1.0 | 0.0 | 1.5 | 0.3 | 0.0 | 2.3 | 0.5 | 1.0 | 1.2 |
| CGZ81_01085 | LBA1881 | 0.5 | 1.0 | 0.0 | 1.5 | 0.3 | 0.0 | 2.3 | 0.5 | 1.0 | 1.2 |
| CGZ81_05865 | mnmA | 1.0 | 0.7 | 0.0 | 0.5 | 0.7 | 0.3 | 0.6 | 1.0 | 2.1 | 1.2 |
| CGZ81_05460 | LBA0746 | 0.5 | 0.3 | 0.3 | 1.5 | 0.0 | 0.3 | 2.3 | 0.5 | 1.0 | 1.2 |
| CGZ81_00485 | LBA1752 | 0.5 | 0.0 | 0.3 | 1.5 | 0.0 | 0.0 | 2.3 | 1.0 | 0.5 | 1.2 |
| CGZ81_01265 | LBA1919 | 0.5 | 0.3 | 0.3 | 1.5 | 0.3 | 0.0 | 2.3 | 1.0 | 0.5 | 1.2 |
| CGZ81_08910 | galK | 4.0 | 1.0 | 0.3 | 2.5 | 0.3 | 1.3 | 0.7 | 0.5 | 2.6 | 1.2 |
| CGZ81_09445 | LBA1572 | 1.0 | 0.7 | 0.7 | 1.0 | 0.7 | 1.3 | 1.0 | 1.0 | 1.7 | 1.2 |
| CGZ81_03120 | secY | 1.0 | 0.0 | 1.0 | 0.0 | 0.7 | 0.0 | 0.2 | 3.2 | 0.2 | 1.2 |
| CGZ81_06830 | LBA1022 | 2.5 | 0.7 | 0.3 | 0.5 | 1.0 | 1.0 | 0.3 | 1.3 | 2.1 | 1.2 |
| CGZ81_00510 | LBA1758 | 2.0 | 0.0 | 0.0 | 1.0 | 0.3 | 0.0 | 0.6 | 2.1 | 1.0 | 1.2 |
| CGZ81_06285 | LBA0906 | 1.0 | 0.3 | 0.3 | 0.5 | 0.7 | 0.7 | 0.6 | 1.5 | 1.5 | 1.2 |
| CGZ81_08955 | galE | 1.0 | 0.7 | 0.3 | 0.5 | 0.7 | 1.0 | 0.6 | 1.0 | 2.1 | 1.2 |
| CGZ81_05835 | ileS | 1.0 | 0.7 | 0.3 | 0.5 | 0.7 | 1.0 | 0.6 | 1.0 | 2.1 | 1.2 |
| CGZ81_03345 | LBA0354 | 1.5 | 0.3 | 0.7 | 0.5 | 0.0 | 2.3 | 0.4 | 0.5 | 2.7 | 1.2 |
| CGZ81_04390 | gatA | 1.0 | 1.0 | 0.0 | 0.5 | 2.3 | 0.0 | 0.6 | 2.0 | 1.0 | 1.2 |
| CGZ81_04905 | LBA0630 | 4.5 | 4.0 | 1.0 | 6.0 | 1.0 | 2.3 | 1.3 | 0.3 | 2.0 | 1.2 |
| CGZ81_02390 | LBA0163 | 0.5 | 0.3 | 0.3 | 1.0 | 0.0 | 0.7 | 1.6 | 0.5 | 1.5 | 1.2 |
| CGZ81_07470 | cpdA | 0.5 | 0.0 | 0.0 | 1.0 | 0.0 | 0.0 | 1.6 | 1.0 | 1.0 | 1.2 |
| CGZ81_09680 | LBA1621 | 0.5 | 0.0 | 0.3 | 1.0 | 0.0 | 0.3 | 1.6 | 1.0 | 1.0 | 1.2 |
| CGZ81_04375 | ligA | 1.0 | 0.3 | 0.7 | 2.0 | 0.7 | 0.0 | 1.8 | 1.5 | 0.3 | 1.2 |
| CGZ81_05055 | LBA0660 | 1.0 | 1.0 | 0.0 | 0.0 | 1.3 | 0.3 | 0.2 | 1.3 | 2.1 | 1.2 |
| CGZ81_06635 | trmFO | 3.0 | 2.3 | 0.3 | 1.0 | 1.3 | 1.3 | 0.4 | 0.6 | 2.6 | 1.2 |
| CGZ81_03560 | LBA0396 | 4.0 | 2.7 | 0.0 | 7.5 | 2.0 | 0.0 | 1.8 | 0.8 | 1.0 | 1.2 |
| CGZ81_07130 | LBA1082 | 1.0 | 0.3 | 0.0 | 1.0 | 0.0 | 0.3 | 1.0 | 0.5 | 2.1 | 1.2 |
| CGZ81_08735 | LBA1420 | 1.5 | 0.3 | 0.0 | 1.5 | 0.0 | 0.3 | 1.0 | 0.5 | 2.1 | 1.2 |
| CGZ81_09635 | LBA1613 | 1.0 | 0.3 | 0.0 | 1.0 | 0.0 | 0.3 | 1.0 | 0.5 | 2.1 | 1.2 |
| CGZ81_03885 | LBA0442 | 3.5 | 0.0 | 0.3 | 1.5 | 0.3 | 0.3 | 0.5 | 2.1 | 1.0 | 1.2 |
| CGZ81_04525 | LBA0554 | 0.5 | 0.7 | 0.0 | 1.5 | 0.0 | 0.0 | 2.3 | 0.3 | 1.0 | 1.2 |
| CGZ81_06670 | LBA0990 | 1.5 | 0.3 | 0.0 | 0.5 | 0.3 | 0.3 | 0.4 | 1.0 | 2.1 | 1.2 |
| CGZ81_07565 | LBA1173 | 1.5 | 0.0 | 0.0 | 0.5 | 0.3 | 0.0 | 0.4 | 2.1 | 1.0 | 1.2 |
| CGZ81_00460 | LBA1747 | 1.5 | 0.0 | 0.0 | 0.5 | 0.3 | 0.0 | 0.4 | 2.1 | 1.0 | 1.2 |
| CGZ81_03580 | scrB | 1.5 | 0.3 | 0.0 | 0.5 | 0.3 | 0.3 | 0.4 | 1.0 | 2.1 | 1.2 |
| CGZ81_05260 | LBA0702 | 5.0 | 1.0 | 0.3 | 1.0 | 1.3 | 1.0 | 0.2 | 1.3 | 2.1 | 1.2 |
| CGZ81_06765 | LBA1010 | 1.5 | 0.3 | 0.0 | 1.5 | 0.7 | 0.0 | 1.0 | 1.5 | 1.0 | 1.2 |
| CGZ81_07925 | LBA1250 | 1.0 | 1.3 | 0.3 | 1.0 | 1.3 | 0.7 | 1.0 | 1.0 | 1.5 | 1.2 |
| CGZ81_09010 | LBA1480 | 0.5 | 0.0 | 0.3 | 0.5 | 0.0 | 0.7 | 1.0 | 1.0 | 1.5 | 1.2 |
| CGZ81_01450 | pepL | 1.0 | 1.3 | 0.0 | 0.5 | 1.0 | 0.3 | 0.6 | 0.8 | 2.1 | 1.2 |
| CGZ81_01385 | LBA1943 | 5.0 | 0.3 | 0.7 | 4.0 | 1.0 | 0.3 | 0.8 | 2.1 | 0.7 | 1.2 |
| CGZ81_09045 | LBA1487 | 0.0 | 0.0 | 0.0 | 0.1 | 0.0 | 0.0 | 1.5 | 1.0 | 1.0 | 1.2 |
| CGZ81_06425 | LBA0937 | 1.5 | 0.7 | 0.0 | 1.0 | 0.3 | 0.3 | 0.7 | 0.7 | 2.1 | 1.2 |
| CGZ81_03460 | LBA0376 | 1.0 | 0.7 | 0.3 | 0.5 | 1.0 | 0.7 | 0.6 | 1.3 | 1.5 | 1.2 |
| CGZ81_01470 | oppA | 1.3 | 0.7 | 0.7 | 1.0 | 2.0 | 0.0 | 0.8 | 2.4 | 0.3 | 1.2 |
| CGZ81_07530 | addA | 2.5 | 1.3 | 0.3 | 2.0 | 0.7 | 1.0 | 0.8 | 0.6 | 2.1 | 1.2 |
| CGZ81_02420 | slpB | 7.2 | 3.7 | 0.3 | 8.5 | 3.3 | 0.6 | 1.2 | 0.9 | 1.4 | 1.2 |
| CGZ81_04330 | nadE | 2.0 | 0.0 | 0.0 | 0.5 | 0.0 | 0.3 | 0.3 | 1.0 | 2.1 | 1.2 |
| CGZ81_09770 | ssdh | 3.0 | 0.3 | 0.0 | 1.0 | 1.0 | 0.0 | 0.4 | 2.1 | 1.0 | 1.1 |
| CGZ81_07915 | LBA1360 | 1.5 | 1.7 | 0.0 | 1.5 | 0.3 | 0.3 | 1.0 | 0.3 | 2.1 | 1.1 |
| CGZ81_01785 | LBA0046 | 0.0 | 0.0 | 0.7 | 0.0 | 0.3 | 0.0 | 1.0 | 2.1 | 0.3 | 1.1 |
| CGZ81_04560 | LBA0562 | 0.0 | 0.0 | 0.7 | 0.0 | 0.3 | 0.0 | 1.0 | 2.1 | 0.3 | 1.1 |
| CGZ81_01755 | LBA0040 | 19.5 | 3.0 | 16.3 | 18.5 | 5.7 | 10.7 | 0.9 | 1.8 | 0.7 | 1.1 |
| CGZ81_08130 | LBA1908 | 2.5 | 0.0 | 0.0 | 0.5 | 0.3 | 0.0 | 0.3 | 2.1 | 1.0 | 1.1 |
| CGZ81_02055 | LBA0094 | 1.0 | 0.0 | 0.0 | 1.5 | 0.0 | 0.0 | 1.4 | 1.0 | 1.0 | 1.1 |
| CGZ81_05450 | LBA0745 | 1.0 | 0.0 | 0.0 | 1.5 | 0.0 | 0.0 | 1.4 | 1.0 | 1.0 | 1.1 |
| CGZ81_06985 | mutT | 1.0 | 0.0 | 0.3 | 1.5 | 0.0 | 0.3 | 1.4 | 1.0 | 1.0 | 1.1 |
| CGZ81_00050 | oppA | 20.5 | 3.0 | 1.7 | 29.0 | 2.3 | 2.0 | 1.4 | 0.8 | 1.2 | 1.1 |
| NA | fmtB | 18.0 | 10.0 | 5.0 | 21.0 | 6.3 | 8.0 | 1.2 | 0.6 | 1.6 | 1.1 |
| CGZ81_02490 | LBA0188 | 2.0 | 0.3 | 0.0 | 1.5 | 0.0 | 0.3 | 0.8 | 0.5 | 2.1 | 1.1 |
| CGZ81_07965 | nusA | 1.0 | 0.3 | 0.7 | 1.0 | 1.0 | 0.0 | 1.0 | 2.1 | 0.3 | 1.1 |
| NA | ydaM | 0.5 | 1.0 | 0.3 | 1.0 | 1.3 | 0.0 | 1.6 | 1.3 | 0.5 | 1.1 |
| CGZ81_05745 | mreC | 1.0 | 0.7 | 0.0 | 1.0 | 1.0 | 0.0 | 1.0 | 1.3 | 1.0 | 1.1 |
| CGZ81_04645 | glnH | 1.0 | 0.0 | 0.0 | 0.0 | 0.3 | 0.0 | 0.2 | 2.1 | 1.0 | 1.1 |
| CGZ81_00090 | glnQ | 1.0 | 0.0 | 0.0 | 0.0 | 0.3 | 0.0 | 0.2 | 2.1 | 1.0 | 1.1 |
| CGZ81_01740 | LBA0037 | 1.0 | 0.0 | 0.0 | 0.0 | 0.3 | 0.0 | 0.2 | 2.1 | 1.0 | 1.1 |
| CGZ81_08420 | LBA1356 | 1.0 | 0.0 | 0.0 | 0.0 | 0.0 | 0.3 | 0.2 | 1.0 | 2.1 | 1.1 |
| CGZ81_00890 | LBA1842 | 1.0 | 0.0 | 0.0 | 0.0 | 0.3 | 0.0 | 0.2 | 2.1 | 1.0 | 1.1 |
| CGZ81_01190 | LBA1902 | 0.0 | 1.0 | 0.0 | 0.0 | 0.0 | 0.3 | 1.0 | 0.2 | 2.1 | 1.1 |
| CGZ81_00470 | nrdI | 1.0 | 0.0 | 0.0 | 0.0 | 0.0 | 0.3 | 0.2 | 1.0 | 2.1 | 1.1 |
| CGZ81_00275 | amyX | 2.5 | 1.0 | 0.7 | 2.0 | 1.7 | 0.7 | 0.8 | 1.5 | 1.0 | 1.1 |
| CGZ81_04240 | msmE | 2.0 | 3.0 | 0.3 | 2.0 | 2.3 | 0.7 | 1.0 | 0.8 | 1.5 | 1.1 |
| CGZ81_02990 | rpoC | 4.0 | 2.0 | 0.3 | 2.5 | 2.3 | 0.7 | 0.7 | 1.1 | 1.5 | 1.1 |
| CGZ81_02120 | LBA0109 | 1.5 | 0.7 | 0.3 | 0.5 | 1.0 | 0.7 | 0.4 | 1.3 | 1.5 | 1.1 |
| CGZ81_07435 | LBA1148 | 1.5 | 0.7 | 0.3 | 0.5 | 1.0 | 0.7 | 0.4 | 1.3 | 1.5 | 1.1 |
| CGZ81_05140 | gpsA | 0.5 | 0.3 | 4.3 | 1.5 | 0.3 | 0.0 | 2.3 | 1.0 | 0.1 | 1.1 |
| CGZ81_05150 | glgB | 2.0 | 0.3 | 0.0 | 1.5 | 0.7 | 0.0 | 0.8 | 1.5 | 1.0 | 1.1 |
| CGZ81_00165 | malH | 1.5 | 0.3 | 0.0 | 1.0 | 0.0 | 0.3 | 0.7 | 0.5 | 2.1 | 1.1 |
| CGZ81_01075 | thiD | 1.0 | 0.7 | 0.3 | 0.5 | 1.3 | 0.3 | 0.6 | 1.7 | 1.0 | 1.1 |
| CGZ81_01015 | LBA1868 | 1.7 | 0.4 | 0.1 | 0.7 | 0.0 | 0.7 | 0.5 | 0.4 | 2.4 | 1.1 |
| CGZ81_05970 | LBA0844 | 0.0 | 1.3 | 0.0 | 0.0 | 0.0 | 0.3 | 1.0 | 0.2 | 2.1 | 1.1 |
| CGZ81_09790 | LBA1637 | 5.5 | 5.0 | 0.7 | 3.0 | 3.3 | 1.7 | 0.6 | 0.7 | 2.0 | 1.1 |
| CGZ81_01925 | LBA0068 | 0.5 | 2.7 | 0.7 | 1.0 | 1.7 | 0.7 | 1.6 | 0.7 | 1.0 | 1.1 |
| CGZ81_05215 | clpP | 0.5 | 0.3 | 1.0 | 0.5 | 1.0 | 0.0 | 1.0 | 2.1 | 0.2 | 1.1 |
| CGZ81_08110 | ffh | 10.0 | 1.3 | 0.7 | 10.0 | 0.7 | 1.3 | 1.0 | 0.6 | 1.7 | 1.1 |
| CGZ81_07585 | LBA1177 | 1.5 | 0.3 | 0.0 | 2.0 | 0.3 | 0.0 | 1.3 | 1.0 | 1.0 | 1.1 |
| CGZ81_04750 | LBA0601 | 1.5 | 0.0 | 0.0 | 0.0 | 0.3 | 0.0 | 0.2 | 2.1 | 1.0 | 1.1 |
| CGZ81_06900 | LBA1037 | 1.5 | 0.0 | 0.0 | 0.0 | 0.3 | 0.0 | 0.2 | 2.1 | 1.0 | 1.1 |
| CGZ81_08710 | LBA1415 | 1.5 | 0.3 | 0.0 | 0.0 | 0.3 | 0.3 | 0.2 | 1.0 | 2.1 | 1.1 |
| CGZ81_04810 | hprT | 1.0 | 0.0 | 1.7 | 1.0 | 0.3 | 0.0 | 1.0 | 2.1 | 0.2 | 1.1 |
| CGZ81_07525 | dinG | 2.5 | 1.0 | 0.7 | 2.5 | 1.3 | 0.7 | 1.0 | 1.3 | 1.0 | 1.1 |
| CGZ81_09035 | rbsK | 1.0 | 1.0 | 0.0 | 2.0 | 0.3 | 0.0 | 1.8 | 0.5 | 1.0 | 1.1 |
| CGZ81_07115 | LBA1079 | 1.5 | 0.0 | 0.3 | 1.0 | 0.0 | 0.7 | 0.7 | 1.0 | 1.5 | 1.1 |
| CGZ81_01005 | LBA1866 | 7.5 | 1.0 | 1.7 | 6.0 | 1.3 | 2.0 | 0.8 | 1.3 | 1.2 | 1.1 |
| CGZ81_08195 | recG | 2.0 | 0.7 | 0.3 | 2.5 | 0.7 | 0.3 | 1.2 | 1.0 | 1.0 | 1.1 |
| CGZ81_01200 | yifK | 2.0 | 0.0 | 0.3 | 2.5 | 0.0 | 0.3 | 1.2 | 1.0 | 1.0 | 1.1 |
| CGZ81_04215 | LBA0496 | 0.5 | 1.3 | 0.0 | 1.0 | 0.7 | 0.0 | 1.6 | 0.6 | 1.0 | 1.1 |
| CGZ81_02210 | LBA0127 | 0.2 | 0.4 | 0.1 | 0.7 | 0.0 | 0.0 | 2.1 | 0.4 | 0.7 | 1.1 |
| CGZ81_01315 | LBA1927 | 1.0 | 1.7 | 0.3 | 1.5 | 1.3 | 0.3 | 1.4 | 0.8 | 1.0 | 1.1 |
| CGZ81_08560 | pyrF | 1.0 | 1.0 | 0.0 | 0.5 | 0.3 | 0.3 | 0.6 | 0.5 | 2.1 | 1.1 |
| CGZ81_06815 | LBA1019 | 2.0 | 3.0 | 2.3 | 4.0 | 1.7 | 1.7 | 1.9 | 0.6 | 0.7 | 1.1 |
| CGZ81_09475 | murC | 0.5 | 1.0 | 0.3 | 1.5 | 0.3 | 0.0 | 2.3 | 0.5 | 0.5 | 1.1 |
| CGZ81_01405 | LBA1947 | 2.0 | 0.3 | 1.0 | 0.0 | 1.0 | 1.0 | 0.1 | 2.1 | 1.0 | 1.1 |
| CGZ81_02450 | LBA0177 | 2.0 | 0.7 | 0.3 | 2.0 | 0.3 | 0.7 | 1.0 | 0.7 | 1.5 | 1.1 |
| CGZ81_08200 | LBA1310 | 2.5 | 0.0 | 0.7 | 2.0 | 0.0 | 1.0 | 0.8 | 1.0 | 1.3 | 1.1 |
| CGZ81_09780 | LBA1634 | 7.0 | 4.0 | 1.0 | 5.5 | 3.3 | 1.7 | 0.8 | 0.8 | 1.5 | 1.1 |
| CGZ81_06450 | LBA0943 | 0.5 | 1.0 | 0.3 | 0.0 | 2.0 | 0.3 | 0.4 | 1.8 | 1.0 | 1.0 |
| CGZ81_09400 | fhs2 | 2.0 | 1.0 | 0.3 | 0.5 | 0.7 | 1.0 | 0.3 | 0.7 | 2.1 | 1.0 |
| CGZ81_04730 | LBA0597 | 3.0 | 2.0 | 1.3 | 2.5 | 2.7 | 1.3 | 0.8 | 1.3 | 1.0 | 1.0 |
| CGZ81_00120 | LBA1680 | 7.0 | 0.7 | 0.7 | 8.0 | 0.3 | 1.0 | 1.1 | 0.7 | 1.3 | 1.0 |
| CGZ81_09225 | lisK | 4.0 | 1.7 | 0.3 | 4.5 | 0.7 | 0.7 | 1.1 | 0.5 | 1.5 | 1.0 |
| CGZ81_00315 | LBA1716 | 0.5 | 1.3 | 0.3 | 0.5 | 0.7 | 0.7 | 1.0 | 0.6 | 1.5 | 1.0 |
| CGZ81_07950 | infB | 3.5 | 1.7 | 1.3 | 2.5 | 2.0 | 1.7 | 0.7 | 1.2 | 1.2 | 1.0 |
| CGZ81_05125 | LBA0675 | 0.5 | 0.3 | 0.0 | 1.0 | 0.0 | 0.0 | 1.6 | 0.5 | 1.0 | 1.0 |
| CGZ81_05290 | potA | 0.5 | 0.3 | 0.3 | 1.0 | 0.0 | 0.3 | 1.6 | 0.5 | 1.0 | 1.0 |
| CGZ81_00410 | epsB | 2.0 | 1.0 | 0.7 | 1.0 | 0.3 | 1.7 | 0.6 | 0.5 | 2.0 | 1.0 |
| CGZ81_00005 | LBA1656 | 1.0 | 0.7 | 0.0 | 2.0 | 0.0 | 0.0 | 1.8 | 0.3 | 1.0 | 1.0 |
| CGZ81_05080 | recA | 1.5 | 0.3 | 0.7 | 1.0 | 0.3 | 1.0 | 0.7 | 1.0 | 1.3 | 1.0 |
| CGZ81_07030 | LBA1064 | 1.5 | 1.0 | 0.0 | 1.0 | 0.0 | 0.3 | 0.7 | 0.2 | 2.1 | 1.0 |
| CGZ81_00445 | LBA1744 | 59.5 | 14.0 | 39.0 | 57.5 | 24.0 | 15.3 | 1.0 | 1.7 | 0.4 | 1.0 |
| CGZ81_06460 | uvrC | 2.5 | 2.0 | 0.3 | 2.0 | 1.3 | 0.7 | 0.8 | 0.7 | 1.5 | 1.0 |
| CGZ81_05810 | ftsZ | 2.5 | 1.0 | 1.0 | 0.5 | 0.7 | 2.3 | 0.3 | 0.7 | 2.0 | 1.0 |
| CGZ81_01045 | LBA1874 | 1.5 | 0.7 | 0.3 | 2.5 | 0.7 | 0.0 | 1.6 | 1.0 | 0.5 | 1.0 |
| CGZ81_01655 | cadB | 1.5 | 0.0 | 0.3 | 0.5 | 0.3 | 0.0 | 0.4 | 2.1 | 0.5 | 1.0 |
| CGZ81_01725 | LBA0034 | 1.5 | 0.3 | 0.0 | 0.5 | 0.0 | 0.3 | 0.4 | 0.5 | 2.1 | 1.0 |
| CGZ81_05145 | trxB | 1.0 | 1.0 | 0.3 | 1.0 | 0.3 | 0.7 | 1.0 | 0.5 | 1.5 | 1.0 |
| CGZ81_04640 | glnQ | 1.5 | 1.3 | 0.0 | 1.5 | 1.3 | 0.0 | 1.0 | 1.0 | 1.0 | 1.0 |
| CGZ81_01780 | LBA0045 | 0.0 | 0.3 | 1.0 | 0.0 | 0.3 | 1.0 | 1.0 | 1.0 | 1.0 | 1.0 |
| CGZ81_02185 | LBA0122 | 3.5 | 0.7 | 0.7 | 3.5 | 0.7 | 0.7 | 1.0 | 1.0 | 1.0 | 1.0 |
| CGZ81_03250 | LBA0336 | 1.0 | 0.7 | 0.0 | 1.0 | 0.7 | 0.0 | 1.0 | 1.0 | 1.0 | 1.0 |
| CGZ81_03290 | LBA0343 | 0.5 | 0.0 | 0.7 | 0.5 | 0.0 | 0.7 | 1.0 | 1.0 | 1.0 | 1.0 |
| CGZ81_05885 | LBA0826 | 1.0 | 0.3 | 0.3 | 1.0 | 0.3 | 0.3 | 1.0 | 1.0 | 1.0 | 1.0 |
| CGZ81_07135 | LBA1083 | 1.5 | 0.0 | 0.0 | 1.5 | 0.0 | 0.0 | 1.0 | 1.0 | 1.0 | 1.0 |
| CGZ81_07295 | LBA1116 | 1.0 | 0.0 | 0.0 | 1.0 | 0.0 | 0.0 | 1.0 | 1.0 | 1.0 | 1.0 |
| CGZ81_07600 | LBA1181 | 0.0 | 0.3 | 0.7 | 0.0 | 0.3 | 0.7 | 1.0 | 1.0 | 1.0 | 1.0 |
| CGZ81_08895 | LBA1455 | 1.0 | 0.3 | 0.3 | 1.0 | 0.3 | 0.3 | 1.0 | 1.0 | 1.0 | 1.0 |
| CGZ81_09270 | LBA1534 | 0.0 | 0.0 | 0.0 | 0.0 | 0.0 | 0.0 | 1.0 | 1.0 | 1.0 | 1.0 |
| CGZ81_09575 | LBA1601 | 1.0 | 0.3 | 0.0 | 1.0 | 0.3 | 0.0 | 1.0 | 1.0 | 1.0 | 1.0 |
| CGZ81_04245 | msmF | 1.5 | 0.0 | 0.0 | 1.5 | 0.0 | 0.0 | 1.0 | 1.0 | 1.0 | 1.0 |
| CGZ81_05190 | uvrA | 2.5 | 1.3 | 0.7 | 2.5 | 1.3 | 0.7 | 1.0 | 1.0 | 1.0 | 1.0 |
| CGZ81_02650 | glmU | 1.0 | 0.0 | 0.7 | 0.0 | 0.3 | 0.3 | 0.2 | 2.1 | 0.7 | 1.0 |
| CGZ81_02935 | tilS | 2.5 | 1.7 | 0.7 | 2.0 | 2.0 | 0.7 | 0.8 | 1.2 | 1.0 | 1.0 |
| CGZ81_00245 | murQ | 2.5 | 0.3 | 0.7 | 1.0 | 0.7 | 0.7 | 0.5 | 1.5 | 1.0 | 1.0 |
| CGZ81_05160 | glgD | 2.5 | 0.7 | 0.0 | 1.5 | 1.0 | 0.0 | 0.6 | 1.3 | 1.0 | 1.0 |
| CGZ81_04555 | LBA0560 | 2.5 | 0.7 | 0.7 | 1.5 | 0.7 | 1.0 | 0.6 | 1.0 | 1.3 | 1.0 |
| CGZ81_00900 | LBA1844 | 1.0 | 1.0 | 0.3 | 2.0 | 0.7 | 0.0 | 1.8 | 0.7 | 0.5 | 1.0 |
| NA | cdpA | 4.0 | 6.3 | 1.0 | 5.0 | 4.7 | 1.0 | 1.2 | 0.7 | 1.0 | 1.0 |
| CGZ81_06035 | asd | 0.5 | 1.0 | 0.0 | 0.0 | 0.3 | 0.3 | 0.4 | 0.5 | 2.1 | 1.0 |
| CGZ81_01160 | asnA | 1.5 | 0.0 | 0.3 | 0.5 | 0.0 | 0.7 | 0.4 | 1.0 | 1.5 | 1.0 |
| CGZ81_03715 | LBA0428 | 1.5 | 0.0 | 0.3 | 0.5 | 0.0 | 0.7 | 0.4 | 1.0 | 1.5 | 1.0 |
| CGZ81_09220 | LBA1523 | 1.0 | 0.7 | 0.0 | 0.5 | 1.0 | 0.0 | 0.6 | 1.3 | 1.0 | 1.0 |
| CGZ81_09525 | LBA1590 | 2.0 | 1.7 | 0.0 | 0.5 | 0.7 | 0.3 | 0.3 | 0.5 | 2.1 | 1.0 |
| CGZ81_01225 | clpE | 3.0 | 3.0 | 2.3 | 4.0 | 2.7 | 1.7 | 1.3 | 0.9 | 0.7 | 1.0 |
| CGZ81_08005 | pyrH | 1.5 | 0.0 | 1.3 | 0.5 | 0.3 | 0.3 | 0.4 | 2.1 | 0.4 | 1.0 |
| CGZ81_07345 | LBA1126 | 4.5 | 2.0 | 0.0 | 7.0 | 0.7 | 0.0 | 1.5 | 0.4 | 1.0 | 1.0 |
| CGZ81_09070 | ackB | 1.5 | 1.0 | 0.7 | 2.0 | 1.0 | 0.3 | 1.3 | 1.0 | 0.7 | 1.0 |
| CGZ81_00270 | LBA1709 | 2.5 | 0.3 | 1.0 | 1.5 | 1.0 | 0.0 | 0.6 | 2.1 | 0.2 | 1.0 |
| CGZ81_06210 | bshA | 2.0 | 0.7 | 0.0 | 1.0 | 1.0 | 0.0 | 0.6 | 1.3 | 1.0 | 1.0 |
| CGZ81_03170 | ecfT | 2.0 | 0.3 | 0.0 | 3.0 | 0.0 | 0.0 | 1.4 | 0.5 | 1.0 | 1.0 |
| CGZ81_06795 | LBA1016 | 0.5 | 0.3 | 0.3 | 0.0 | 1.0 | 0.0 | 0.4 | 2.1 | 0.5 | 1.0 |
| CGZ81_03380 | rplA | 1.5 | 0.3 | 8.0 | 4.0 | 0.0 | 0.0 | 2.4 | 0.5 | 0.0 | 1.0 |
| CGZ81_07935 | ribC | 1.0 | 0.3 | 0.7 | 2.0 | 0.0 | 0.3 | 1.8 | 0.5 | 0.7 | 1.0 |
| CGZ81_08945 | lacL | 1.2 | 0.0 | 0.0 | 1.0 | 0.0 | 0.0 | 0.9 | 1.0 | 1.0 | 1.0 |
| CGZ81_08385 | LBA1349 | 0.5 | 1.3 | 0.7 | 0.0 | 1.0 | 1.3 | 0.4 | 0.8 | 1.7 | 1.0 |
| CGZ81_06590 | cca | 1.0 | 0.3 | 0.0 | 1.5 | 0.0 | 0.0 | 1.4 | 0.5 | 1.0 | 1.0 |
| CGZ81_07475 | LBA1155 | 1.0 | 0.3 | 0.0 | 1.5 | 0.0 | 0.0 | 1.4 | 0.5 | 1.0 | 1.0 |
| CGZ81_00805 | LBA1823 | 1.5 | 1.3 | 1.3 | 0.5 | 1.0 | 2.3 | 0.4 | 0.8 | 1.6 | 1.0 |
| CGZ81_01070 | glpK | 3.0 | 0.3 | 0.3 | 2.5 | 0.3 | 0.3 | 0.8 | 1.0 | 1.0 | 0.9 |
| CGZ81_05065 | fabG | 0.0 | 0.7 | 0.3 | 0.0 | 0.0 | 0.7 | 1.0 | 0.3 | 1.5 | 0.9 |
| CGZ81_05050 | LBA0659 | 0.5 | 0.3 | 0.7 | 0.5 | 0.7 | 0.0 | 1.0 | 1.5 | 0.3 | 0.9 |
| CGZ81_04030 | lysR | 1.0 | 0.7 | 0.3 | 1.0 | 0.0 | 0.7 | 1.0 | 0.3 | 1.5 | 0.9 |
| CGZ81_06730 | LBA1002 | 1.0 | 1.7 | 0.0 | 0.0 | 0.7 | 0.3 | 0.2 | 0.5 | 2.1 | 0.9 |
| CGZ81_00435 | LBA1741 | 1.0 | 1.0 | 0.0 | 0.0 | 0.3 | 0.3 | 0.2 | 0.5 | 2.1 | 0.9 |
| CGZ81_07375 | LBA1131 | 2.0 | 1.0 | 0.3 | 1.0 | 1.3 | 0.3 | 0.6 | 1.3 | 1.0 | 0.9 |
| CGZ81_06040 | LBA0858 | 5.0 | 1.3 | 5.0 | 6.5 | 2.0 | 0.3 | 1.3 | 1.4 | 0.1 | 0.9 |
| CGZ81_03585 | scrA | 1.5 | 0.7 | 1.0 | 1.0 | 1.0 | 0.7 | 0.7 | 1.3 | 0.7 | 0.9 |
| CGZ81_02720 | pyrG | 2.5 | 0.7 | 0.0 | 1.0 | 1.0 | 0.0 | 0.5 | 1.3 | 1.0 | 0.9 |
| CGZ81_09620 | LBA1609 | 0.5 | 0.3 | 1.3 | 1.0 | 0.3 | 0.0 | 1.6 | 1.0 | 0.2 | 0.9 |
| CGZ81_01260 | lysA | 7.5 | 1.0 | 1.0 | 12.0 | 0.7 | 0.3 | 1.6 | 0.7 | 0.5 | 0.9 |
| CGZ81_08250 | fmt | 1.0 | 1.0 | 0.7 | 1.5 | 0.7 | 0.3 | 1.4 | 0.7 | 0.7 | 0.9 |
| CGZ81_07450 | LBA1151 | 0.5 | 1.3 | 0.3 | 0.0 | 2.0 | 0.3 | 0.4 | 1.4 | 1.0 | 0.9 |
| CGZ81_04335 | pacL | 2.0 | 1.3 | 1.0 | 1.0 | 1.7 | 1.0 | 0.6 | 1.2 | 1.0 | 0.9 |
| CGZ81_00440 | LBA1743 | 1.0 | 2.0 | 0.0 | 0.0 | 0.7 | 0.3 | 0.2 | 0.4 | 2.1 | 0.9 |
| CGZ81_04660 | LBA0583 | 1.0 | 0.3 | 0.3 | 0.0 | 0.0 | 1.0 | 0.2 | 0.5 | 2.1 | 0.9 |
| CGZ81_05805 | ftsA | 1.0 | 0.7 | 0.3 | 0.0 | 0.7 | 0.7 | 0.2 | 1.0 | 1.5 | 0.9 |
| CGZ81_06495 | yfkN | 1.0 | 0.3 | 0.3 | 0.0 | 0.3 | 0.7 | 0.2 | 1.0 | 1.5 | 0.9 |
| CGZ81_01325 | LBA1929 | 5.0 | 1.3 | 0.3 | 2.0 | 1.0 | 0.7 | 0.4 | 0.8 | 1.5 | 0.9 |
| CGZ81_08080 | LBA1284 | 1.5 | 0.0 | 0.3 | 0.0 | 0.3 | 0.0 | 0.2 | 2.1 | 0.5 | 0.9 |
| CGZ81_05700 | thiI | 1.5 | 0.0 | 0.3 | 0.0 | 0.3 | 0.0 | 0.2 | 2.1 | 0.5 | 0.9 |
| CGZ81_03660 | alaS | 4.0 | 2.0 | 4.0 | 3.5 | 1.3 | 4.7 | 0.9 | 0.7 | 1.2 | 0.9 |
| CGZ81_09370 | purL | 2.0 | 0.7 | 0.7 | 3.0 | 0.3 | 0.3 | 1.4 | 0.7 | 0.7 | 0.9 |
| CGZ81_04205 | LBA0494 | 1.5 | 1.0 | 0.0 | 1.5 | 0.7 | 0.0 | 1.0 | 0.7 | 1.0 | 0.9 |
| CGZ81_07400 | LBA1140 | 1.0 | 1.0 | 1.0 | 1.0 | 1.0 | 0.7 | 1.0 | 1.0 | 0.7 | 0.9 |
| CGZ81_02085 | LBA0101 | 0.5 | 0.3 | 0.7 | 0.0 | 1.0 | 0.0 | 0.4 | 2.1 | 0.3 | 0.9 |
| CGZ81_02110 | LBA0107 | 1.5 | 1.0 | 0.3 | 1.0 | 0.3 | 0.7 | 0.7 | 0.5 | 1.5 | 0.9 |
| CGZ81_04890 | hmdH | 1.5 | 0.7 | 1.0 | 1.0 | 0.7 | 1.0 | 0.7 | 1.0 | 1.0 | 0.9 |
| CGZ81_00750 | LBA1812 | 1.5 | 0.0 | 0.0 | 1.0 | 0.0 | 0.0 | 0.7 | 1.0 | 1.0 | 0.9 |
| CGZ81_09285 | rpmI | 1.5 | 0.0 | 0.0 | 1.0 | 0.0 | 0.0 | 0.7 | 1.0 | 1.0 | 0.9 |
| CGZ81_03565 | LBA0397 | 2.5 | 1.0 | 0.0 | 1.0 | 1.3 | 0.0 | 0.5 | 1.3 | 1.0 | 0.9 |
| CGZ81_00105 | LBA1677 | 0.5 | 1.0 | 0.0 | 0.0 | 0.0 | 0.3 | 0.4 | 0.2 | 2.1 | 0.9 |
| CGZ81_09275 | LBA1535 | 4.0 | 0.3 | 0.3 | 0.5 | 0.7 | 0.3 | 0.2 | 1.5 | 1.0 | 0.9 |
| CGZ81_06805 | LBA1018 | 2.0 | 2.0 | 0.0 | 1.0 | 2.3 | 0.0 | 0.6 | 1.1 | 1.0 | 0.9 |
| CGZ81_03560 | LBA1362 | 0.0 | 1.3 | 0.3 | 0.0 | 0.0 | 0.7 | 1.0 | 0.2 | 1.5 | 0.9 |
| CGZ81_02330 | LBA0151 | 4.0 | 2.3 | 3.3 | 5.0 | 3.3 | 0.0 | 1.2 | 1.4 | 0.1 | 0.9 |
| CGZ81_07645 | LBA1191 | 1.5 | 0.0 | 0.3 | 0.0 | 0.0 | 0.7 | 0.2 | 1.0 | 1.5 | 0.9 |
| CGZ81_08235 | LBA1317 | 2.0 | 1.0 | 0.3 | 2.5 | 1.0 | 0.0 | 1.2 | 1.0 | 0.5 | 0.9 |
| CGZ81_01620 | LBA0014 | 19.0 | 16.7 | 9.0 | 26.0 | 15.3 | 3.3 | 1.4 | 0.9 | 0.4 | 0.9 |
| CGZ81_01850 | LBA0058 | 2.5 | 3.0 | 0.7 | 3.0 | 1.3 | 0.7 | 1.2 | 0.5 | 1.0 | 0.9 |
| CGZ81_00875 | LBA1839 | 4.5 | 2.3 | 2.0 | 4.5 | 3.0 | 0.7 | 1.0 | 1.3 | 0.4 | 0.9 |
| CGZ81_09865 | LBA1652 | 1.5 | 1.3 | 0.3 | 2.0 | 0.3 | 0.3 | 1.3 | 0.4 | 1.0 | 0.9 |
| CGZ81_04125 | LBA0478 | 0.0 | 1.7 | 0.0 | 0.0 | 1.0 | 0.0 | 1.0 | 0.7 | 1.0 | 0.9 |
| CGZ81_08820 | LBA1437 | 1.0 | 1.7 | 0.3 | 1.0 | 1.0 | 0.3 | 1.0 | 0.7 | 1.0 | 0.9 |
| CGZ81_08725 | LBA1418 | 2.0 | 0.7 | 0.0 | 2.0 | 0.3 | 0.0 | 1.0 | 0.7 | 1.0 | 0.9 |
| CGZ81_00960 | nisT | 2.5 | 1.7 | 0.0 | 2.0 | 1.3 | 0.0 | 0.8 | 0.8 | 1.0 | 0.9 |
| CGZ81_00415 | epsA | 4.0 | 0.3 | 0.3 | 2.5 | 0.3 | 0.3 | 0.7 | 1.0 | 1.0 | 0.9 |
| CGZ81_01535 | mnmG | 3.5 | 0.7 | 1.0 | 1.5 | 1.3 | 0.3 | 0.5 | 1.7 | 0.5 | 0.9 |
| CGZ81_09085 | LBA1496 | 2.0 | 2.7 | 0.3 | 0.5 | 2.0 | 0.7 | 0.3 | 0.8 | 1.5 | 0.9 |
| CGZ81_08455 | LBA1366 | 2.5 | 0.7 | 0.0 | 1.5 | 0.7 | 0.0 | 0.6 | 1.0 | 1.0 | 0.9 |
| CGZ81_09345 | purD | 5.5 | 1.0 | 3.0 | 6.0 | 0.7 | 2.3 | 1.1 | 0.7 | 0.8 | 0.9 |
| NA | epsJ | 0.0 | 2.3 | 0.0 | 0.0 | 1.3 | 0.0 | 1.0 | 0.6 | 1.0 | 0.9 |
| NA | apbE | 2.0 | 0.3 | 0.7 | 1.5 | 0.7 | 0.0 | 0.8 | 1.5 | 0.3 | 0.9 |
| CGZ81_06340 | citF | 4.0 | 5.7 | 0.3 | 2.5 | 2.3 | 0.7 | 0.7 | 0.4 | 1.5 | 0.9 |
| CGZ81_06335 | citE | 1.0 | 0.7 | 0.0 | 0.5 | 0.7 | 0.0 | 0.6 | 1.0 | 1.0 | 0.9 |
| CGZ81_06220 | LBA0893 | 1.0 | 0.0 | 0.0 | 0.5 | 0.0 | 0.0 | 0.6 | 1.0 | 1.0 | 0.9 |
| CGZ81_06555 | LBA0966 | 1.0 | 0.0 | 0.0 | 0.5 | 0.0 | 0.0 | 0.6 | 1.0 | 1.0 | 0.9 |
| CGZ81_01155 | LBA1895 | 1.0 | 0.3 | 0.7 | 0.5 | 0.3 | 0.7 | 0.6 | 1.0 | 1.0 | 0.9 |
| CGZ81_08685 | lysR | 1.0 | 0.3 | 0.0 | 0.5 | 0.3 | 0.0 | 0.6 | 1.0 | 1.0 | 0.9 |
| CGZ81_03575 | scrR | 1.0 | 0.0 | 0.3 | 0.5 | 0.0 | 0.3 | 0.6 | 1.0 | 1.0 | 0.9 |
| CGZ81_09020 | rbsC | 3.5 | 0.7 | 0.3 | 0.0 | 0.7 | 0.7 | 0.1 | 1.0 | 1.5 | 0.9 |
| CGZ81_08190 | plsX | 0.5 | 1.3 | 1.3 | 1.0 | 0.3 | 0.7 | 1.6 | 0.4 | 0.6 | 0.9 |
| CGZ81_01285 | dltD | 4.5 | 0.7 | 1.7 | 5.0 | 1.0 | 0.0 | 1.1 | 1.3 | 0.2 | 0.9 |
| CGZ81_00260 | LBA1707 | 1.5 | 1.0 | 1.7 | 2.0 | 0.3 | 1.3 | 1.3 | 0.5 | 0.8 | 0.9 |
| CGZ81_01730 | LBA0035 | 4.0 | 0.3 | 1.0 | 1.0 | 1.0 | 0.0 | 0.3 | 2.1 | 0.2 | 0.9 |
| CGZ81_04115 | LBA0476 | 1.5 | 0.7 | 1.0 | 1.5 | 1.0 | 0.0 | 1.0 | 1.3 | 0.2 | 0.9 |
| CGZ81_07205 | LBA1098 | 1.0 | 0.7 | 1.0 | 0.0 | 1.0 | 1.0 | 0.2 | 1.3 | 1.0 | 0.9 |
| CGZ81_00115 | LBA1679 | 0.5 | 0.3 | 0.3 | 1.0 | 0.0 | 0.0 | 1.6 | 0.5 | 0.5 | 0.9 |
| CGZ81_01855 | pbpX | 0.5 | 0.3 | 0.3 | 1.0 | 0.0 | 0.0 | 1.6 | 0.5 | 0.5 | 0.9 |
| CGZ81_00250 | LBA1705 | 4.0 | 2.0 | 1.3 | 4.0 | 1.0 | 1.3 | 1.0 | 0.6 | 1.0 | 0.9 |
| CGZ81_01220 | LBA1909 | 2.0 | 0.3 | 0.3 | 1.0 | 0.3 | 0.3 | 0.6 | 1.0 | 1.0 | 0.9 |
| CGZ81_03975 | rnr | 1.5 | 2.0 | 0.0 | 1.5 | 1.0 | 0.0 | 1.0 | 0.6 | 1.0 | 0.9 |
| CGZ81_01370 | LBA1939 | 2.5 | 1.7 | 1.0 | 1.5 | 1.0 | 1.3 | 0.6 | 0.7 | 1.3 | 0.9 |
| CGZ81_06150 | LBA0880 | 0.5 | 3.3 | 0.0 | 0.5 | 1.7 | 0.0 | 1.0 | 0.5 | 1.0 | 0.8 |
| CGZ81_05325 | LBA0717 | 1.8 | 0.0 | 0.7 | 1.5 | 0.0 | 0.3 | 0.9 | 1.0 | 0.7 | 0.8 |
| CGZ81_00780 | murE | 0.5 | 1.3 | 0.7 | 1.0 | 0.7 | 0.0 | 1.6 | 0.6 | 0.3 | 0.8 |
| CGZ81_07105 | LBA1077 | 1.0 | 1.3 | 0.0 | 0.0 | 0.0 | 0.3 | 0.2 | 0.2 | 2.1 | 0.8 |
| CGZ81_02885 | cshA | 1.0 | 0.7 | 0.3 | 1.5 | 0.3 | 0.0 | 1.4 | 0.7 | 0.5 | 0.8 |
| CGZ81_01965 | LBA1907 | 0.3 | 0.2 | 0.0 | 0.0 | 0.2 | 0.0 | 0.5 | 1.0 | 1.0 | 0.8 |
| CGZ81_08765 | LBA1426 | 7.0 | 17.7 | 9.3 | 6.0 | 24.3 | 2.3 | 0.9 | 1.4 | 0.3 | 0.8 |
| CGZ81_06515 | kpyK | 29.5 | 2.7 | 27.3 | 41.0 | 2.7 | 3.0 | 1.4 | 1.0 | 0.1 | 0.8 |
| CGZ81_08975 | LBA1473 | 3.0 | 0.7 | 0.7 | 2.5 | 0.7 | 0.3 | 0.8 | 1.0 | 0.7 | 0.8 |
| CGZ81_03090 | rpsH | 9.5 | 0.0 | 0.7 | 8.0 | 0.0 | 0.3 | 0.8 | 1.0 | 0.7 | 0.8 |
| CGZ81_09065 | glpK | 1.5 | 1.0 | 0.3 | 2.0 | 0.7 | 0.0 | 1.3 | 0.7 | 0.5 | 0.8 |
| CGZ81_09565 | fbaA | 9.5 | 0.3 | 1.3 | 10.5 | 0.3 | 0.3 | 1.1 | 1.0 | 0.4 | 0.8 |
| CGZ81_08720 | LBA1417 | 0.0 | 1.0 | 0.0 | 0.0 | 0.3 | 0.0 | 1.0 | 0.5 | 1.0 | 0.8 |
| CGZ81_08785 | LBA1430 | 0.0 | 1.0 | 0.0 | 0.0 | 0.3 | 0.0 | 1.0 | 0.5 | 1.0 | 0.8 |
| CGZ81_08925 | lacZ | 2.5 | 1.7 | 0.3 | 0.0 | 0.3 | 1.0 | 0.1 | 0.3 | 2.1 | 0.8 |
| CGZ81_01840 | exoA | 0.5 | 0.7 | 0.3 | 0.5 | 0.7 | 0.0 | 1.0 | 1.0 | 0.5 | 0.8 |
| CGZ81_06140 | LBA0878 | 1.5 | 1.0 | 0.3 | 1.5 | 1.0 | 0.0 | 1.0 | 1.0 | 0.5 | 0.8 |
| CGZ81_06390 | LBA0930 | 1.0 | 0.3 | 0.3 | 1.0 | 0.3 | 0.0 | 1.0 | 1.0 | 0.5 | 0.8 |
| CGZ81_07265 | LBA1111 | 1.0 | 0.3 | 0.0 | 1.0 | 0.0 | 0.0 | 1.0 | 0.5 | 1.0 | 0.8 |
| CGZ81_08435 | LBA1359 | 1.0 | 0.3 | 0.3 | 1.0 | 0.3 | 0.0 | 1.0 | 1.0 | 0.5 | 0.8 |
| CGZ81_04370 | LBA1676 | 1.5 | 0.3 | 1.0 | 1.5 | 0.0 | 1.0 | 1.0 | 0.5 | 1.0 | 0.8 |
| CGZ81_02400 | pepO | 1.5 | 2.3 | 0.3 | 1.0 | 1.7 | 0.3 | 0.7 | 0.7 | 1.0 | 0.8 |
| CGZ81_06470 | LBA0948 | 4.5 | 1.3 | 2.0 | 5.0 | 1.0 | 1.0 | 1.1 | 0.8 | 0.6 | 0.8 |
| CGZ81_01745 | LBA0038 | 1.0 | 0.3 | 0.7 | 0.5 | 0.7 | 0.0 | 0.6 | 1.5 | 0.3 | 0.8 |
| NA | atpD | 2.5 | 0.7 | 0.0 | 0.0 | 1.0 | 0.0 | 0.1 | 1.3 | 1.0 | 0.8 |
| CGZ81_01090 | cobQ | 1.5 | 0.0 | 0.0 | 0.5 | 0.0 | 0.0 | 0.4 | 1.0 | 1.0 | 0.8 |
| CGZ81_08905 | galT | 1.5 | 0.3 | 0.3 | 0.5 | 0.3 | 0.3 | 0.4 | 1.0 | 1.0 | 0.8 |
| CGZ81_08860 | LBA1446 | 1.5 | 0.7 | 0.3 | 0.5 | 0.7 | 0.3 | 0.4 | 1.0 | 1.0 | 0.8 |
| CGZ81_00795 | LBA1821 | 3.5 | 0.7 | 0.7 | 4.0 | 0.7 | 0.0 | 1.1 | 1.0 | 0.3 | 0.8 |
| CGZ81_07380 | LBA1132 | 1.5 | 2.7 | 0.0 | 1.5 | 1.0 | 0.0 | 1.0 | 0.4 | 1.0 | 0.8 |
| CGZ81_00830 | parB | 3.0 | 1.7 | 0.3 | 0.5 | 1.0 | 0.7 | 0.2 | 0.7 | 1.5 | 0.8 |
| CGZ81_08985 | LBA1475 | 0.7 | 0.1 | 0.1 | 0.7 | 0.0 | 0.0 | 1.0 | 0.7 | 0.7 | 0.8 |
| CGZ81_09320 | dnaB | 5.0 | 4.3 | 0.3 | 3.0 | 3.3 | 0.3 | 0.6 | 0.8 | 1.0 | 0.8 |
| CGZ81_03555 | frc | 0.5 | 1.0 | 0.7 | 0.5 | 0.7 | 0.3 | 1.0 | 0.7 | 0.7 | 0.8 |
| CGZ81_04045 | adhE | 2.0 | 5.0 | 1.3 | 1.5 | 4.0 | 1.0 | 0.8 | 0.8 | 0.8 | 0.8 |
| CGZ81_05640 | mreB | 0.0 | 1.3 | 0.0 | 0.0 | 0.3 | 0.0 | 1.0 | 0.4 | 1.0 | 0.8 |
| CGZ81_07700 | phoH | 0.0 | 1.3 | 0.0 | 0.0 | 0.3 | 0.0 | 1.0 | 0.4 | 1.0 | 0.8 |
| CGZ81_01395 | LBA1945 | 1.5 | 0.0 | 0.7 | 1.0 | 0.0 | 0.3 | 0.7 | 1.0 | 0.7 | 0.8 |
| CGZ81_05210 | whiA | 1.5 | 0.7 | 0.3 | 1.0 | 0.3 | 0.3 | 0.7 | 0.7 | 1.0 | 0.8 |
| CGZ81_01980 | LBA0078 | 0.5 | 0.7 | 0.0 | 0.0 | 0.7 | 0.0 | 0.4 | 1.0 | 1.0 | 0.8 |
| CGZ81_04670 | LBA0585 | 6.5 | 3.3 | 1.3 | 5.5 | 2.3 | 1.0 | 0.9 | 0.7 | 0.8 | 0.8 |
| CGZ81_03125 | adk | 1.0 | 2.3 | 0.0 | 1.0 | 0.7 | 0.0 | 1.0 | 0.4 | 1.0 | 0.8 |
| CGZ81_07540 | mvaK | 2.0 | 0.3 | 1.0 | 0.5 | 0.7 | 0.3 | 0.3 | 1.5 | 0.5 | 0.8 |
| CGZ81_06405 | relA | 1.0 | 1.3 | 1.0 | 0.5 | 1.3 | 0.7 | 0.6 | 1.0 | 0.7 | 0.8 |
| CGZ81_09775 | LBA1633 | 6.5 | 1.7 | 1.0 | 5.0 | 1.3 | 0.7 | 0.8 | 0.8 | 0.7 | 0.8 |
| NA | coaA | 1.0 | 1.3 | 0.3 | 0.0 | 0.7 | 0.7 | 0.2 | 0.6 | 1.5 | 0.8 |
| CGZ81_03970 | LBA0448 | 1.5 | 0.7 | 0.3 | 0.0 | 0.3 | 0.7 | 0.2 | 0.7 | 1.5 | 0.8 |
| CGZ81_00335 | LBA1719 | 2.0 | 0.0 | 0.0 | 0.5 | 0.0 | 0.0 | 0.3 | 1.0 | 1.0 | 0.8 |
| CGZ81_01965 | LBA0077 | 0.7 | 0.8 | 0.3 | 0.5 | 0.5 | 0.2 | 0.8 | 0.7 | 0.8 | 0.8 |
| CGZ81_03495 | LBA0383 | 2.0 | 1.3 | 0.0 | 0.0 | 1.7 | 0.0 | 0.1 | 1.2 | 1.0 | 0.8 |
| CGZ81_00420 | hflX | 2.0 | 1.0 | 0.3 | 0.5 | 1.7 | 0.0 | 0.3 | 1.5 | 0.5 | 0.8 |
| CGZ81_05320 | glmM | 0.5 | 0.3 | 1.0 | 1.0 | 0.0 | 0.0 | 1.6 | 0.5 | 0.2 | 0.8 |
| CGZ81_04885 | thil | 5.5 | 1.7 | 1.0 | 3.0 | 1.7 | 0.7 | 0.6 | 1.0 | 0.7 | 0.8 |
| CGZ81_01585 | gyrA | 1.5 | 0.7 | 0.0 | 1.5 | 0.0 | 0.0 | 1.0 | 0.3 | 1.0 | 0.8 |
| CGZ81_05105 | LBA0671 | 0.0 | 0.7 | 0.0 | 0.0 | 0.0 | 0.0 | 1.0 | 0.3 | 1.0 | 0.8 |
| CGZ81_00060 | LBA1667 | 1.0 | 0.7 | 0.0 | 1.0 | 0.0 | 0.0 | 1.0 | 0.3 | 1.0 | 0.8 |
| CGZ81_01540 | mnmE | 0.5 | 0.7 | 0.7 | 0.5 | 0.7 | 0.0 | 1.0 | 1.0 | 0.3 | 0.8 |
| CGZ81_09330 | coaE | 1.5 | 0.7 | 0.7 | 1.5 | 0.3 | 0.3 | 1.0 | 0.7 | 0.7 | 0.8 |
| CGZ81_00065 | efp1 | 0.0 | 0.7 | 0.7 | 0.0 | 0.3 | 0.3 | 1.0 | 0.7 | 0.7 | 0.8 |
| CGZ81_05345 | LBA0723 | 2.5 | 0.3 | 0.7 | 0.5 | 0.3 | 0.7 | 0.3 | 1.0 | 1.0 | 0.8 |
| CGZ81_01955 | LBA0074 | 1.5 | 0.7 | 0.3 | 0.5 | 0.0 | 0.7 | 0.4 | 0.3 | 1.5 | 0.8 |
| CGZ81_00020 | LBA1659 | 1.0 | 3.3 | 1.7 | 1.0 | 1.3 | 1.3 | 1.0 | 0.4 | 0.8 | 0.8 |
| CGZ81_00340 | LBA1720 | 0.6 | 0.1 | 0.0 | 0.1 | 0.0 | 0.0 | 0.5 | 0.8 | 1.0 | 0.8 |
| CGZ81_03700 | murI | 1.0 | 0.0 | 0.7 | 0.5 | 0.0 | 0.3 | 0.6 | 1.0 | 0.7 | 0.8 |
| CGZ81_09595 | pspC | 1.0 | 0.7 | 0.0 | 0.5 | 0.3 | 0.0 | 0.6 | 0.7 | 1.0 | 0.8 |
| CGZ81_07145 | LBA1086 | 0.0 | 2.3 | 0.0 | 0.0 | 0.3 | 0.0 | 1.0 | 0.2 | 1.0 | 0.7 |
| CGZ81_00400 | epsD | 1.0 | 0.0 | 0.0 | 0.0 | 0.0 | 0.0 | 0.2 | 1.0 | 1.0 | 0.7 |
| CGZ81_02545 | LBA0199 | 0.0 | 1.0 | 0.3 | 0.0 | 0.0 | 0.3 | 1.0 | 0.2 | 1.0 | 0.7 |
| CGZ81_04445 | LBA0543 | 1.0 | 0.0 | 0.0 | 0.0 | 0.0 | 0.0 | 0.2 | 1.0 | 1.0 | 0.7 |
| CGZ81_08215 | LBA1313 | 0.0 | 1.0 | 0.0 | 0.0 | 0.0 | 0.0 | 1.0 | 0.2 | 1.0 | 0.7 |
| CGZ81_08355 | LBA1343 | 1.0 | 0.0 | 0.0 | 0.0 | 0.0 | 0.0 | 0.2 | 1.0 | 1.0 | 0.7 |
| CGZ81_00290 | LBA1713 | 1.0 | 0.0 | 0.3 | 0.0 | 0.0 | 0.3 | 0.2 | 1.0 | 1.0 | 0.7 |
| CGZ81_09005 | licT | 1.0 | 0.0 | 0.0 | 0.0 | 0.0 | 0.0 | 0.2 | 1.0 | 1.0 | 0.7 |
| CGZ81_09480 | pheT | 1.0 | 0.3 | 0.0 | 0.0 | 0.3 | 0.0 | 0.2 | 1.0 | 1.0 | 0.7 |
| CGZ81_09430 | LBA1568 | 2.5 | 2.0 | 0.3 | 1.0 | 2.7 | 0.0 | 0.5 | 1.3 | 0.5 | 0.7 |
| CGZ81_06880 | LBA1033 | 2.5 | 1.3 | 0.3 | 0.0 | 0.7 | 0.7 | 0.1 | 0.6 | 1.5 | 0.7 |
| CGZ81_02870 | LBA0264 | 1.5 | 0.3 | 0.3 | 2.0 | 0.0 | 0.0 | 1.3 | 0.5 | 0.5 | 0.7 |
| CGZ81_06395 | prmA | 0.5 | 0.3 | 1.0 | 0.5 | 0.0 | 0.7 | 1.0 | 0.5 | 0.7 | 0.7 |
| CGZ81_08535 | pyrC | 3.5 | 1.7 | 0.7 | 0.5 | 1.0 | 1.0 | 0.2 | 0.7 | 1.3 | 0.7 |
| CGZ81_01040 | ackA | 1.5 | 0.0 | 0.3 | 1.0 | 0.0 | 0.0 | 0.7 | 1.0 | 0.5 | 0.7 |
| CGZ81_00395 | epsE | 1.5 | 0.0 | 0.3 | 1.0 | 0.0 | 0.0 | 0.7 | 1.0 | 0.5 | 0.7 |
| CGZ81_09335 | mutM | 1.5 | 0.3 | 0.3 | 1.0 | 0.3 | 0.0 | 0.7 | 1.0 | 0.5 | 0.7 |
| CGZ81_05175 | LBA0686 | 1.5 | 1.0 | 0.3 | 0.5 | 0.7 | 0.3 | 0.4 | 0.7 | 1.0 | 0.7 |
| CGZ81_01125 | purB | 2.0 | 1.3 | 1.3 | 2.0 | 0.7 | 0.7 | 1.0 | 0.6 | 0.6 | 0.7 |
| CGZ81_04120 | LBA0477 | 0.0 | 1.3 | 0.0 | 0.0 | 0.0 | 0.0 | 1.0 | 0.2 | 1.0 | 0.7 |
| CGZ81_06625 | LBA0980 | 0.0 | 0.3 | 1.3 | 0.0 | 0.3 | 0.0 | 1.0 | 1.0 | 0.2 | 0.7 |
| CGZ81_07920 | hrcA | 1.0 | 2.3 | 0.7 | 0.0 | 4.0 | 0.0 | 0.2 | 1.6 | 0.3 | 0.7 |
| CGZ81_05615 | atpA | 1.5 | 0.0 | 0.0 | 0.0 | 0.0 | 0.0 | 0.2 | 1.0 | 1.0 | 0.7 |
| CGZ81_07415 | LBA1144 | 1.5 | 0.0 | 0.0 | 0.0 | 0.0 | 0.0 | 0.2 | 1.0 | 1.0 | 0.7 |
| CGZ81_07505 | nth | 1.5 | 0.0 | 0.0 | 0.0 | 0.0 | 0.0 | 0.2 | 1.0 | 1.0 | 0.7 |
| CGZ81_03075 | rplX | 1.5 | 0.3 | 0.0 | 0.0 | 0.3 | 0.0 | 0.2 | 1.0 | 1.0 | 0.7 |
| CGZ81_08015 | rpsB | 4.0 | 0.3 | 5.7 | 4.5 | 0.3 | 0.0 | 1.1 | 1.0 | 0.1 | 0.7 |
| CGZ81_03425 | rplJ | 1.5 | 0.7 | 4.0 | 2.0 | 0.3 | 0.7 | 1.3 | 0.7 | 0.2 | 0.7 |
| CGZ81_08105 | rpsP | 1.0 | 0.0 | 1.7 | 1.0 | 0.0 | 0.0 | 1.0 | 1.0 | 0.2 | 0.7 |
| NA | rnj | 2.0 | 0.3 | 1.3 | 0.5 | 0.3 | 1.0 | 0.3 | 1.0 | 0.8 | 0.7 |
| CGZ81_09410 | LBA1564 | 0.0 | 1.0 | 0.7 | 0.0 | 0.3 | 0.3 | 1.0 | 0.5 | 0.7 | 0.7 |
| CGZ81_02560 | oppD | 3.0 | 1.0 | 1.3 | 2.0 | 0.0 | 1.7 | 0.7 | 0.2 | 1.2 | 0.7 |
| CGZ81_06290 | fumC | 0.5 | 0.3 | 0.7 | 0.5 | 0.0 | 0.3 | 1.0 | 0.5 | 0.7 | 0.7 |
| CGZ81_00515 | LBA1759 | 3.0 | 3.7 | 0.7 | 1.5 | 2.0 | 0.7 | 0.5 | 0.6 | 1.0 | 0.7 |
| CGZ81_01560 | dnaA | 3.5 | 1.7 | 1.3 | 2.5 | 1.7 | 0.3 | 0.7 | 1.0 | 0.4 | 0.7 |
| NA | ftsQ | 0.5 | 1.0 | 0.0 | 0.0 | 0.7 | 0.0 | 0.4 | 0.7 | 1.0 | 0.7 |
| CGZ81_03015 | rpsJ | 1.0 | 1.0 | 2.3 | 2.0 | 0.0 | 0.0 | 1.8 | 0.2 | 0.1 | 0.7 |
| CGZ81_08255 | priA | 1.0 | 1.0 | 1.0 | 0.5 | 0.7 | 0.7 | 0.6 | 0.7 | 0.7 | 0.7 |
| CGZ81_01790 | LBA0047 | 1.5 | 0.7 | 0.0 | 0.5 | 0.3 | 0.0 | 0.4 | 0.7 | 1.0 | 0.7 |
| CGZ81_01995 | yycI | 1.5 | 0.7 | 0.0 | 0.5 | 0.3 | 0.0 | 0.4 | 0.7 | 1.0 | 0.7 |
| CGZ81_09195 | pheT | 2.5 | 1.7 | 1.3 | 1.0 | 1.3 | 1.0 | 0.5 | 0.8 | 0.8 | 0.7 |
| CGZ81_06935 | LBA1046 | 1.0 | 0.3 | 0.0 | 0.5 | 0.0 | 0.0 | 0.6 | 0.5 | 1.0 | 0.7 |
| CGZ81_08020 | LBA1271 | 1.0 | 0.3 | 0.0 | 0.5 | 0.0 | 0.0 | 0.6 | 0.5 | 1.0 | 0.7 |
| CGZ81_00850 | LBA1833 | 1.0 | 0.0 | 0.3 | 0.5 | 0.0 | 0.0 | 0.6 | 1.0 | 0.5 | 0.7 |
| CGZ81_05565 | LBA0765 | 3.0 | 0.7 | 0.3 | 0.5 | 0.0 | 0.7 | 0.2 | 0.3 | 1.5 | 0.7 |
| CGZ81_07670 | dnaG | 1.5 | 1.0 | 0.7 | 0.0 | 1.3 | 0.3 | 0.2 | 1.3 | 0.7 | 0.7 |
| CGZ81_02695 | LBA0228 | 2.5 | 0.7 | 1.3 | 2.0 | 0.3 | 0.7 | 0.8 | 0.7 | 0.6 | 0.7 |
| CGZ81_01960 | LBA0075 | 1.0 | 0.3 | 0.7 | 0.0 | 0.7 | 0.0 | 0.2 | 1.5 | 0.3 | 0.7 |
| CGZ81_06430 | LBA0939 | 1.0 | 1.3 | 0.7 | 0.5 | 1.0 | 0.3 | 0.6 | 0.8 | 0.7 | 0.7 |
| CGZ81_00685 | plnG | 2.0 | 1.7 | 1.0 | 2.0 | 0.3 | 0.7 | 1.0 | 0.3 | 0.7 | 0.7 |
| CGZ81_00085 | LBA1672 | 0.0 | 0.3 | 1.3 | 0.0 | 0.0 | 0.7 | 1.0 | 0.5 | 0.6 | 0.7 |
| CGZ81_03550 | LBA0394 | 3.5 | 1.3 | 0.0 | 1.5 | 0.7 | 0.0 | 0.5 | 0.6 | 1.0 | 0.7 |
| CGZ81_07425 | LBA1146 | 2.0 | 0.3 | 0.3 | 1.0 | 0.3 | 0.0 | 0.6 | 1.0 | 0.5 | 0.7 |
| CGZ81_08915 | LBA1460 | 2.5 | 2.0 | 0.3 | 1.0 | 1.0 | 0.3 | 0.5 | 0.6 | 1.0 | 0.7 |
| CGZ81_06195 | eno | 37.5 | 10.0 | 29.3 | 43.0 | 8.0 | 2.0 | 1.1 | 0.8 | 0.1 | 0.7 |
| CGZ81_03720 | LBA0429 | 1.0 | 1.3 | 0.0 | 0.0 | 1.0 | 0.0 | 0.2 | 0.8 | 1.0 | 0.7 |
| CGZ81_08045 | LBA1277 | 1.0 | 1.3 | 0.3 | 0.0 | 1.0 | 0.3 | 0.2 | 0.8 | 1.0 | 0.7 |
| CGZ81_04235 | msmR | 1.5 | 1.0 | 1.3 | 1.5 | 0.0 | 1.0 | 1.0 | 0.2 | 0.8 | 0.7 |
| CGZ81_01170 | trxR | 3.5 | 1.7 | 0.3 | 1.0 | 0.0 | 0.7 | 0.3 | 0.2 | 1.5 | 0.7 |
| CGZ81_09185 | LBA1516 | 1.0 | 3.3 | 1.0 | 0.5 | 3.0 | 0.3 | 0.6 | 0.9 | 0.5 | 0.7 |
| CGZ81_03315 | cysS | 1.0 | 1.3 | 0.0 | 0.5 | 0.3 | 0.0 | 0.6 | 0.4 | 1.0 | 0.7 |
| CGZ81_08865 | LBA1447 | 1.5 | 1.7 | 1.0 | 2.0 | 0.7 | 0.0 | 1.3 | 0.5 | 0.2 | 0.7 |
| CGZ81_08010 | tsf | 8.0 | 0.3 | 16.7 | 7.5 | 0.3 | 0.7 | 0.9 | 1.0 | 0.1 | 0.7 |
| CGZ81_02015 | LBA1862 | 3.5 | 1.3 | 1.3 | 1.0 | 1.0 | 1.1 | 0.3 | 0.8 | 0.9 | 0.7 |
| CGZ81_08155 | oppA | 204.5 | 79.3 | 122.0 | 148.3 | 81.5 | 29.3 | 0.7 | 1.0 | 0.2 | 0.7 |
| CGZ81_07515 | LBA1162 | 0.0 | 2.3 | 1.0 | 0.0 | 1.0 | 0.3 | 1.0 | 0.5 | 0.5 | 0.7 |
| CGZ81_09830 | LBA1645 | 2.5 | 1.0 | 1.7 | 2.5 | 0.3 | 0.7 | 1.0 | 0.5 | 0.5 | 0.7 |
| NA | htrA | 3.0 | 0.7 | 2.0 | 2.0 | 0.7 | 0.3 | 0.7 | 1.0 | 0.3 | 0.7 |
| CGZ81_05880 | recD2 | 5.0 | 2.0 | 0.7 | 0.5 | 0.0 | 1.3 | 0.2 | 0.1 | 1.7 | 0.7 |
| CGZ81_08830 | LBA1439 | 4.0 | 3.7 | 2.3 | 3.0 | 1.5 | 1.7 | 0.8 | 0.5 | 0.7 | 0.7 |
| CGZ81_04700 | gntR | 0.5 | 1.0 | 0.3 | 0.5 | 0.3 | 0.0 | 1.0 | 0.5 | 0.5 | 0.7 |
| CGZ81_08800 | LBA1433 | 4.0 | 0.7 | 0.3 | 1.0 | 0.3 | 0.3 | 0.3 | 0.7 | 1.0 | 0.7 |
| NA | rpsK | 3.5 | 0.0 | 1.0 | 0.5 | 0.0 | 0.7 | 0.2 | 1.0 | 0.7 | 0.7 |
| CGZ81_01055 | LBA1876 | 1.5 | 1.0 | 0.7 | 1.0 | 0.0 | 0.7 | 0.7 | 0.2 | 1.0 | 0.7 |
| CGZ81_08690 | LBA1411 | 3.0 | 1.3 | 2.0 | 3.0 | 0.3 | 1.0 | 1.0 | 0.4 | 0.6 | 0.7 |
| CGZ81_04010 | manL | 2.5 | 3.3 | 1.7 | 2.0 | 2.0 | 0.7 | 0.8 | 0.6 | 0.5 | 0.6 |
| NA | rpsC | 1.5 | 1.0 | 0.3 | 1.0 | 0.7 | 0.0 | 0.7 | 0.7 | 0.5 | 0.6 |
| CGZ81_04855 | LBA0620 | 1.4 | 0.2 | 0.0 | 0.4 | 0.0 | 0.0 | 0.4 | 0.6 | 1.0 | 0.6 |
| CGZ81_04315 | tagA | 1.0 | 1.0 | 0.7 | 0.5 | 1.0 | 0.0 | 0.6 | 1.0 | 0.3 | 0.6 |
| CGZ81_07690 | era | 1.5 | 0.3 | 0.3 | 0.5 | 0.0 | 0.3 | 0.4 | 0.5 | 1.0 | 0.6 |
| CGZ81_03520 | LBA0388 | 1.5 | 0.3 | 0.3 | 0.5 | 0.3 | 0.0 | 0.4 | 1.0 | 0.5 | 0.6 |
| CGZ81_07500 | LBA1159 | 4.0 | 2.3 | 2.7 | 3.5 | 0.7 | 1.7 | 0.9 | 0.4 | 0.7 | 0.6 |
| CGZ81_07245 | LBA1107 | 1.0 | 1.3 | 1.0 | 0.5 | 1.0 | 0.3 | 0.6 | 0.8 | 0.5 | 0.6 |
| CGZ81_01580 | gyrB | 1.5 | 1.0 | 0.3 | 0.0 | 1.3 | 0.0 | 0.2 | 1.3 | 0.5 | 0.6 |
| CGZ81_00370 | LBA1728 | 2.5 | 2.7 | 0.3 | 1.5 | 2.0 | 0.0 | 0.6 | 0.8 | 0.5 | 0.6 |
| CGZ81_08265 | gmk | 1.0 | 0.7 | 0.0 | 0.0 | 0.3 | 0.0 | 0.2 | 0.7 | 1.0 | 0.6 |
| CGZ81_08230 | rsgA | 1.0 | 0.7 | 0.3 | 0.0 | 0.3 | 0.3 | 0.2 | 0.7 | 1.0 | 0.6 |
| CGZ81_00430 | LBA1740 | 7.0 | 2.3 | 2.0 | 2.5 | 1.0 | 2.0 | 0.4 | 0.5 | 1.0 | 0.6 |
| CGZ81_05245 | pgk | 5.5 | 0.7 | 4.3 | 5.5 | 0.3 | 0.7 | 1.0 | 0.7 | 0.2 | 0.6 |
| CGZ81_00810 | ychF | 0.5 | 0.3 | 2.3 | 0.5 | 0.0 | 0.7 | 1.0 | 0.5 | 0.4 | 0.6 |
| CGZ81_00480 | LBA1751 | 0.0 | 1.3 | 0.7 | 0.0 | 0.0 | 0.3 | 1.0 | 0.2 | 0.7 | 0.6 |
| CGZ81_04615 | LBA0573 | 1.5 | 1.3 | 0.0 | 0.5 | 0.3 | 0.0 | 0.4 | 0.4 | 1.0 | 0.6 |
| CGZ81_08370 | oppA | 1.5 | 1.3 | 0.7 | 0.5 | 0.3 | 0.7 | 0.4 | 0.4 | 1.0 | 0.6 |
| CGZ81_05915 | ftsW | 1.0 | 1.3 | 0.3 | 0.0 | 0.7 | 0.3 | 0.2 | 0.6 | 1.0 | 0.6 |
| CGZ81_05980 | tig | 5.5 | 0.7 | 12.7 | 6.0 | 0.3 | 0.7 | 1.1 | 0.7 | 0.1 | 0.6 |
| CGZ81_03035 | rplB | 2.0 | 0.7 | 0.3 | 0.0 | 0.3 | 0.3 | 0.1 | 0.7 | 1.0 | 0.6 |
| CGZ81_02395 | LBA0164 | 1.0 | 0.7 | 0.3 | 1.0 | 0.0 | 0.0 | 1.0 | 0.3 | 0.5 | 0.6 |
| CGZ81_04300 | LBA0515 | 0.0 | 0.3 | 0.7 | 0.0 | 0.0 | 0.0 | 1.0 | 0.5 | 0.3 | 0.6 |
| CGZ81_00550 | lctP | 0.5 | 0.3 | 0.7 | 0.5 | 0.0 | 0.0 | 1.0 | 0.5 | 0.3 | 0.6 |
| CGZ81_03115 | rplO | 3.0 | 0.3 | 0.7 | 3.0 | 0.0 | 0.0 | 1.0 | 0.5 | 0.3 | 0.6 |
| CGZ81_05790 | murD | 2.5 | 1.0 | 0.0 | 0.5 | 0.3 | 0.0 | 0.3 | 0.5 | 1.0 | 0.6 |
| CGZ81_06010 | dapH | 0.5 | 0.3 | 1.3 | 0.0 | 0.3 | 0.3 | 0.4 | 1.0 | 0.4 | 0.6 |
| CGZ81_03375 | rplK | 2.5 | 0.3 | 0.3 | 0.5 | 0.3 | 0.0 | 0.3 | 1.0 | 0.5 | 0.6 |
| CGZ81_06815 | LBA1020 | 4.5 | 3.7 | 1.7 | 3.0 | 2.0 | 0.7 | 0.7 | 0.6 | 0.5 | 0.6 |
| CGZ81_03210 | ftsK | 1.5 | 1.3 | 0.0 | 0.0 | 0.7 | 0.0 | 0.2 | 0.6 | 1.0 | 0.6 |
| CGZ81_07535 | rexB | 3.5 | 5.7 | 0.7 | 2.0 | 2.7 | 0.3 | 0.6 | 0.5 | 0.7 | 0.6 |
| CGZ81_00595 | fruK | 2.5 | 0.3 | 3.0 | 1.0 | 0.3 | 0.7 | 0.5 | 1.0 | 0.3 | 0.6 |
| CGZ81_06270 | LBA0903 | 9.0 | 1.3 | 2.3 | 2.5 | 1.7 | 0.3 | 0.3 | 1.2 | 0.2 | 0.6 |
| CGZ81_03030 | rplW | 2.0 | 0.3 | 1.0 | 1.5 | 0.0 | 0.3 | 0.8 | 0.5 | 0.5 | 0.6 |
| CGZ81_06325 | citC | 3.0 | 0.7 | 0.3 | 0.5 | 0.7 | 0.0 | 0.2 | 1.0 | 0.5 | 0.6 |
| CGZ81_01815 | LBA0052 | 1.0 | 0.3 | 0.3 | 0.0 | 0.0 | 0.3 | 0.2 | 0.5 | 1.0 | 0.6 |
| CGZ81_06315 | LBA0912 | 1.0 | 0.0 | 0.3 | 0.0 | 0.0 | 0.0 | 0.2 | 1.0 | 0.5 | 0.6 |
| CGZ81_00025 | LBA1660 | 0.5 | 1.0 | 0.3 | 0.5 | 0.0 | 0.0 | 1.0 | 0.2 | 0.5 | 0.6 |
| CGZ81_04975 | mecA | 1.0 | 0.0 | 0.3 | 0.0 | 0.0 | 0.0 | 0.2 | 1.0 | 0.5 | 0.6 |
| CGZ81_05965 | rnj | 0.5 | 1.7 | 0.0 | 0.0 | 0.3 | 0.0 | 0.4 | 0.3 | 1.0 | 0.6 |
| CGZ81_02470 | LBA0184 | 2.5 | 1.0 | 0.0 | 1.0 | 0.0 | 0.0 | 0.5 | 0.2 | 1.0 | 0.6 |
| CGZ81_00175 | LBA1691 | 0.5 | 0.0 | 0.7 | 0.0 | 0.0 | 0.0 | 0.4 | 1.0 | 0.3 | 0.6 |
| CGZ81_01575 | recF | 0.5 | 0.7 | 0.7 | 0.0 | 0.7 | 0.0 | 0.4 | 1.0 | 0.3 | 0.6 |
| CGZ81_05730 | LBA0796 | 0.5 | 0.7 | 0.3 | 0.0 | 0.0 | 0.3 | 0.4 | 0.3 | 1.0 | 0.6 |
| CGZ81_08245 | LBA1319 | 0.5 | 0.7 | 0.0 | 0.0 | 0.0 | 0.0 | 0.4 | 0.3 | 1.0 | 0.6 |
| CGZ81_09415 | LBA1565 | 0.5 | 0.7 | 0.0 | 0.0 | 0.0 | 0.0 | 0.4 | 0.3 | 1.0 | 0.6 |
| CGZ81_06135 | LBA0877 | 2.0 | 0.7 | 1.3 | 1.5 | 0.0 | 0.7 | 0.8 | 0.3 | 0.6 | 0.6 |
| CGZ81_05465 | LBA0747 | 2.5 | 1.0 | 0.3 | 1.0 | 0.7 | 0.0 | 0.5 | 0.7 | 0.5 | 0.6 |
| CGZ81_07975 | polC | 2.0 | 1.7 | 1.0 | 0.5 | 1.3 | 0.3 | 0.3 | 0.8 | 0.5 | 0.6 |
| CGZ81_08590 | LBA1392 | 8.0 | 4.7 | 2.3 | 6.0 | 3.0 | 0.3 | 0.8 | 0.7 | 0.2 | 0.6 |
| CGZ81_08970 | rbsR | 2.0 | 0.7 | 0.3 | 0.5 | 0.0 | 0.3 | 0.3 | 0.3 | 1.0 | 0.6 |
| CGZ81_05560 | LBA0764 | 1.5 | 7.7 | 0.7 | 0.0 | 6.3 | 0.3 | 0.2 | 0.8 | 0.7 | 0.6 |
| CGZ81_08595 | LBA1393 | 1.5 | 1.0 | 1.0 | 0.0 | 0.7 | 0.7 | 0.2 | 0.7 | 0.7 | 0.6 |
| CGZ81_08145 | rnc | 1.5 | 0.3 | 0.3 | 0.0 | 0.0 | 0.3 | 0.2 | 0.5 | 1.0 | 0.5 |
| CGZ81_01610 | rplI | 1.5 | 0.3 | 0.0 | 0.0 | 0.0 | 0.0 | 0.2 | 0.5 | 1.0 | 0.5 |
| CGZ81_00125 | LBA1681 | 2.5 | 3.0 | 1.3 | 1.5 | 0.3 | 1.0 | 0.6 | 0.2 | 0.8 | 0.5 |
| CGZ81_07835 | lysR | 1.0 | 0.7 | 0.7 | 1.0 | 0.0 | 0.0 | 1.0 | 0.3 | 0.3 | 0.5 |
| CGZ81_08310 | LBA1334 | 0.5 | 2.3 | 0.0 | 0.0 | 0.3 | 0.0 | 0.4 | 0.2 | 1.0 | 0.5 |
| CGZ81_06855 | LBA1027 | 2.0 | 0.3 | 0.0 | 0.0 | 0.0 | 0.0 | 0.1 | 0.5 | 1.0 | 0.5 |
| CGZ81_05945 | LBA0839 | 2.0 | 0.0 | 0.3 | 0.0 | 0.0 | 0.0 | 0.1 | 1.0 | 0.5 | 0.5 |
| CGZ81_02445 | LBA0176 | 2.5 | 1.7 | 0.3 | 1.0 | 1.0 | 0.0 | 0.5 | 0.7 | 0.5 | 0.5 |
| CGZ81_00530 | lytR | 2.5 | 2.0 | 0.7 | 1.0 | 0.0 | 0.7 | 0.5 | 0.1 | 1.0 | 0.5 |
| CGZ81_08060 | lexA | 2.5 | 0.3 | 0.3 | 1.5 | 0.0 | 0.0 | 0.6 | 0.5 | 0.5 | 0.5 |
| CGZ81_08135 | ftsY | 2.0 | 2.3 | 0.7 | 1.0 | 0.7 | 0.3 | 0.6 | 0.4 | 0.7 | 0.5 |
| CGZ81_00925 | pepN | 2.0 | 0.3 | 1.0 | 0.5 | 0.3 | 0.0 | 0.3 | 1.0 | 0.2 | 0.5 |
| CGZ81_03505 | rsmI | 1.0 | 1.0 | 0.3 | 0.5 | 0.3 | 0.0 | 0.6 | 0.5 | 0.5 | 0.5 |
| CGZ81_06750 | LBA1006 | 3.5 | 0.3 | 1.7 | 2.0 | 0.0 | 0.7 | 0.6 | 0.5 | 0.5 | 0.5 |
| CGZ81_09665 | LBA1619 | 3.5 | 0.3 | 0.0 | 0.0 | 0.0 | 0.0 | 0.1 | 0.5 | 1.0 | 0.5 |
| CGZ81_04610 | glnH | 2.0 | 2.3 | 0.3 | 1.0 | 1.0 | 0.0 | 0.6 | 0.5 | 0.5 | 0.5 |
| CGZ81_05575 | prfA | 2.0 | 0.7 | 0.7 | 1.0 | 0.0 | 0.3 | 0.6 | 0.3 | 0.7 | 0.5 |
| CGZ81_04160 | LBA0484 | 1.0 | 1.3 | 0.7 | 0.5 | 0.7 | 0.0 | 0.6 | 0.6 | 0.3 | 0.5 |
| CGZ81_06700 | LBA0996 | 4.0 | 1.0 | 2.3 | 2.0 | 0.3 | 1.0 | 0.5 | 0.5 | 0.5 | 0.5 |
| CGZ81_04805 | LBA0612 | 0.5 | 1.7 | 0.3 | 0.0 | 1.0 | 0.0 | 0.4 | 0.7 | 0.5 | 0.5 |
| CGZ81_07010 | LBA1060 | 0.5 | 0.7 | 0.3 | 0.0 | 0.3 | 0.0 | 0.4 | 0.7 | 0.5 | 0.5 |
| CGZ81_05240 | LBA0698 | 44.5 | 13.7 | 12.7 | 31.0 | 10.3 | 0.0 | 0.7 | 0.8 | 0.0 | 0.5 |
| CGZ81_02130 | LBA0111 | 1.5 | 0.7 | 0.0 | 0.0 | 0.0 | 0.0 | 0.2 | 0.3 | 1.0 | 0.5 |
| CGZ81_04085 | LBA0470 | 2.0 | 0.7 | 0.3 | 0.5 | 0.3 | 0.0 | 0.3 | 0.7 | 0.5 | 0.5 |
| CGZ81_02845 | LBA0259 | 3.0 | 1.0 | 0.0 | 0.5 | 0.0 | 0.0 | 0.2 | 0.2 | 1.0 | 0.5 |
| CGZ81_03535 | deoC | 1.0 | 1.0 | 0.0 | 0.0 | 0.0 | 0.0 | 0.2 | 0.2 | 1.0 | 0.5 |
| CGZ81_08160 | oppA | 3.0 | 1.0 | 0.7 | 0.7 | 0.3 | 0.3 | 0.3 | 0.5 | 0.7 | 0.5 |
| CGZ81_03640 | zwf | 1.5 | 1.0 | 1.0 | 0.5 | 0.3 | 0.3 | 0.4 | 0.5 | 0.5 | 0.5 |
| CGZ81_04140 | ftsK | 1.5 | 0.7 | 0.7 | 0.5 | 0.3 | 0.0 | 0.4 | 0.7 | 0.3 | 0.5 |
| CGZ81_04305 | gntR | 1.0 | 0.7 | 0.3 | 0.5 | 0.0 | 0.0 | 0.6 | 0.3 | 0.5 | 0.5 |
| CGZ81_09850 | LBA1649 | 1.0 | 0.3 | 0.7 | 0.5 | 0.0 | 0.0 | 0.6 | 0.5 | 0.3 | 0.5 |
| CGZ81_08345 | ilvE | 1.5 | 1.0 | 0.0 | 0.0 | 0.0 | 0.0 | 0.2 | 0.2 | 1.0 | 0.5 |
| CGZ81_00555 | LBA1769 | 3.0 | 0.3 | 1.7 | 0.5 | 0.3 | 0.0 | 0.2 | 1.0 | 0.2 | 0.5 |
| CGZ81_06630 | topA | 4.0 | 1.7 | 1.0 | 1.0 | 0.3 | 0.7 | 0.3 | 0.3 | 0.7 | 0.5 |
| CGZ81_05695 | nifS | 2.0 | 1.0 | 0.3 | 0.0 | 0.7 | 0.0 | 0.1 | 0.7 | 0.5 | 0.4 |
| CGZ81_03045 | rplV | 6.0 | 0.3 | 3.3 | 4.0 | 0.0 | 0.3 | 0.7 | 0.5 | 0.2 | 0.4 |
| CGZ81_02535 | oppA | 1.5 | 0.7 | 1.0 | 0.0 | 0.3 | 0.3 | 0.2 | 0.7 | 0.5 | 0.4 |
| CGZ81_03470 | LBA0378 | 6.5 | 3.0 | 0.3 | 4.5 | 0.0 | 0.0 | 0.7 | 0.1 | 0.5 | 0.4 |
| CGZ81_02880 | murF | 2.0 | 0.3 | 1.0 | 1.0 | 0.0 | 0.0 | 0.6 | 0.5 | 0.2 | 0.4 |
| CGZ81_05270 | LBA0704 | 2.5 | 1.0 | 1.0 | 0.5 | 0.0 | 0.7 | 0.3 | 0.2 | 0.7 | 0.4 |
| CGZ81_02525 | pepG | 3.0 | 0.3 | 0.3 | 0.5 | 0.0 | 0.0 | 0.2 | 0.5 | 0.5 | 0.4 |
| CGZ81_03190 | LBA0325 | 2.5 | 1.0 | 1.3 | 0.0 | 0.3 | 0.7 | 0.1 | 0.5 | 0.6 | 0.4 |
| CGZ81_04515 | LBA0552 | 0.5 | 0.3 | 0.7 | 0.0 | 0.0 | 0.0 | 0.4 | 0.5 | 0.3 | 0.4 |
| CGZ81_04600 | LBA0570 | 2.0 | 0.3 | 0.7 | 0.5 | 0.0 | 0.0 | 0.3 | 0.5 | 0.3 | 0.4 |
| CGZ81_04400 | LBA0534 | 0.5 | 0.3 | 1.0 | 0.0 | 0.0 | 0.0 | 0.4 | 0.5 | 0.2 | 0.4 |
| CGZ81_02655 | LBA0220 | 1.5 | 0.7 | 1.3 | 0.0 | 0.0 | 0.7 | 0.2 | 0.3 | 0.6 | 0.4 |
| CGZ81_03020 | rplC | 3.0 | 1.0 | 1.7 | 0.5 | 0.3 | 0.0 | 0.2 | 0.5 | 0.2 | 0.3 |
| CGZ81_09685 | metK | 0.5 | 1.0 | 1.0 | 0.0 | 0.0 | 0.0 | 0.4 | 0.2 | 0.2 | 0.3 |
| CGZ81_00930 | lysM | 1.5 | 2.7 | 3.0 | 0.0 | 1.3 | 0.0 | 0.2 | 0.6 | 0.1 | 0.3 |
| CGZ81_00015 | LBA1658 | 2.5 | 0.3 | 3.7 | 0.0 | 0.0 | 0.0 | 0.1 | 0.5 | 0.1 | 0.2 |
| CGZ81_03010 | fusA | 21.0 | 1.0 | 14.0 | 7.5 | 0.0 | 0.3 | 0.4 | 0.2 | 0.0 | 0.2 |
