# Supplementary material for: Lactobacillus acidophilus Membrane Vesicles as a Vehicle of Bacteriocin Delivery
Source: Front Microbiol. 2020 Apr 30;11:710. doi: 10.3389/fmicb.2020.00710 (PMC7203471; doi:10.3389/fmicb.2020.00710)
Supplement: Supplementary file 2 [file Table_2.DOCX]

Supplemental Information


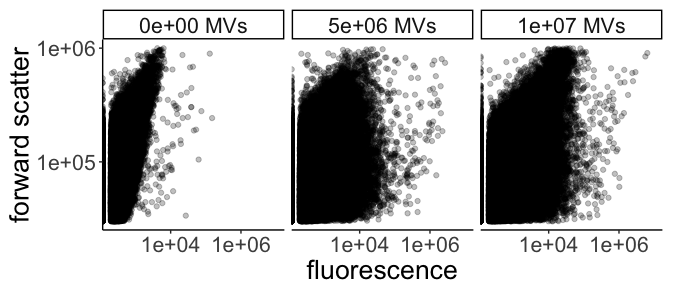


**Figure S1. Membrane fusion of MVs with *E. coli*.** Flow cytometry plots of forward scatter over FL1 fluorescence (533/30 nm) showing *E. coli* BL21(DE3) incubated with increasing amounts of DiO-labeled MVs purified from LabIP-treated *L. acidophilus* cultures.
